# Proceedings of the Eighteenth International Society of Sports Nutrition (ISSN) conference and expo

**DOI:** 10.1080/15502783.2022.2056381

**Published:** 2022-05-20

**Comparison of Skeletal Muscle Ultrastructural Changes Between Normal and Blood Flow Restricted Resistance Exercise: A Case Study**

Dylan T. Wilburn^a^, Steven B. Machek^a^, Bernd Zechmann^b^, Darryn S. Willoughby^c^, FISSN

^a^Exercise & Biochemical Nutrition Laboratory, Department of Health, Human Performance, & Recreation, Robbins College of Health and Human Sciences, Baylor University, Waco, TX, USA; ^b^Center for Microscopy and Imaging, Baylor University, Waco, Texas, USA; ^c^School of Exercise and Sport Science, University of Mary Hardin-Baylor, Belton, TX, USA

Corresponding author: Dylan_Wilburn1@baylor.edu

**Background**: Blood flow restriction training (BFR) partially restricts arterial blood flow while completely restricting venous return from the target limb(s), creating greater metabolic stress and cell swelling during exercise. Studies utilizing BFR have provided conflicting evidence as to whether BFR causes muscle damage and its acute effects on skeletal muscle are unknown. The purpose of this study was to compare the changes in skeletal muscle ultrastructure before and 30 min after normal and BFR resistance training using transmission electron microscopy (TEM).

**Methods**: One apparently healthy male with at least a year of resistance exercise experience participated in this study. The first visit consisted of unilateral angled leg press 1-RM testing on each leg. Foot positioning and knee angle ROM were standardized at each visit. For visit two, in a randomized crossover design, the participant completed six sets (30, 15, 15, 15, max-rep, max-rep) of BFR unilateral leg press at 30% 1-RM with 80% occlusion, followed by six sets (10, 10, 10, 10 max-rep max-rep) of normal unilateral leg press at 70% 1-RM on the contralateral leg. Vastus lateralis biopsies were collected before and 30-min after each exercise condition and subsequently fixed and imaged using TEM. For each sampling point, the lengths (Z-disc to Z-Disc) and widths (M-band) of 250 sarcomeres (1000 total) were measured from TEM-micrographs using CellSens Dimension and analyzed using separate ANCOVAs to account for baseline differences between conditions. Additionally, sarcoplasmic area was measured from 20 individual TEM-micrographs using CellSens Dimensions and analyzed using a 2 × 2 ANOVA with repeated measures. All statistical tests were conducted at a significance value of p < .05.

**Results**: ANCOVA indicated significant differences between lengths of sarcomeres after BFR training (1.769 ± 0.12 µm) when compared to normal exercise (1.64 ± 0.17 µm) (F = 71.58, p < .001, η^2^ = .126). ANCOVA indicated that there was not a significant difference in sarcomere widths post-exercise between BFR (0.90 ± 0.26 µm) and normal exercise (0.93 ± 0.27 µm) (F = 1.871, p = .172, η^2^ = .004). No significant interaction (F = 2.06, p = .168, η^2^ = .098) or group effect between BFR (25.98 ± 4.17%) and normal (27.39 ± 6.49%) resistance exercise was found for sarcoplasmic area (F = 1.547, p = .229, η^2^ = .075). However, there was a time effect, showing increases in sarcoplasmic area within the myofiber from pre-exercise (24.42 ± 5.13%) to post-exercise (28.95 ± 5.92%) (F = 15.426, p = .001, η^2^ = .448).

**Conclusion**: BFR training induced a unique alteration in myofibril ultrastructure that appeared wave-like and was accompanied by intracellular abnormalities appearing to be fluid pockets of sarcoplasm that disrupt surrounding myofibrils. Despite the appearance, no difference in sarcoplasmic area or widths of sarcomeres was present between BFR and normal exercise. BFR training may elicit a unique structural alteration in sarcomere organization that is likely caused by fluid pooling within the muscle during exercise.

**Characterizing Protein Metabolism and Muscle Characteristics Across the Menopause Transition**

Lacey M. Gould^a^, Hannah E. Cabre^a,b^, Amanda N. Gordon^a^, Andrew T. Hoyle^a^, Abbie E. Smith-Ryan^a,b^, Katie R. Hirsch^c^, Arny A. Ferrando^c^, FISSN

^a^Department of Exercise and Sport Science, University of North Carolina at Chapel Hill, Chapel Hill, NC, USA; ^b^Department of Allied Health Science, University of North Carolina at Chapel Hill, Chapel Hill, NC, USA; ^c^Center for Translational Research in Aging & Longevity, University of Arkansas for Medical Sciences, Little Rock, AR, USA

Corresponding author: lmgould@live.unc.edu

**Background**: The menopause transition yields significant metabolic and physiologic alterations. Understanding whole-body protein metabolism may help explain the natural changes (i.e. increased body fat and decreased lean mass [LM]) attributed to menopause. The purpose of this study was to assess protein metabolism across the menopause transition as well as to identify the relationship between muscle characteristics and LM.

**Methods**: Twenty-seven healthy females participated in this study (Mean ± Standard Deviation [SD]: Age: 49.3 ± 6.8 years, BMI: 23.9 ± 3.7 kg/m^2^), with three subgroups determined by menopause status: pre-menopause (PRE; n = 9), peri-menopause (PERI; n = 9), and post-menopause (POST; n = 9). To measure whole-body protein metabolism, participants ingested a 2.00 g dose of [^15^N]alanine. Isotopically labeled nitrogen from blood and urine samples was used to determine whole-body net balance (NB), protein synthesis (PS), protein breakdown (PB), and nitrogen flux. Whole-body LM was measured via dual-energy x-ray absorptiometry and muscle characteristics (muscle size [mCSA] and echo intensity [EI]) were measured via B-mode ultrasound of the dominant-leg vastus lateralis muscle. One-way ANOVAs with Bonferroni post-hoc pairwise comparisons were used to compare protein turnover measures, LM, and muscle characteristics across groups. Bivariate correlations were used to assess relationships between protein turnover measures and muscle characteristics.

**Results**: NB was significantly different across groups (p = 0.040); NB was greater in PRE (Mean ± SD: 1.03 ± 0.75 g/kg of body mass [BM]/day), compared to PERI (Mean ± SD: 0.53 ± 0.30 g/kg BM/day, p = 0.108) and POST (Mean ± SD: 0.48 ± 0.16 g/kg BM/day, p = 0.065). While not statistically significant (p > 0.05), EI was considerably lower in PRE (Mean ± SD: 105.52 ± 19.03 a.u.) compared to PERI (Mean ± SD: 121.30 ± 32.52 a.u., p = 0.532) and POST (Mean ± SD:

115.15 ± 17.85 a.u., p = 1.000). A nonsignificant (p > 0.05) but notable difference in mCSA was observed between PRE (Mean ± SD: 16.52 ± 2.62 cm^2^) and POST (Mean ± SD: 14.82 ± 4.37 cm^2^). EI was moderately inversely correlated to PS (R = −0.436, p = 0.024) as well as flux (R = −0.415, p = 0.031). No significant correlations were observed between LM, mCSA, and measures of protein turnover.

**Conclusions**: Menopause appears to have the greatest impact on whole-body net balance during the onset of menopause (i.e. from pre- to peri-menopause), with decreases sustained into post-menopause. Measures of protein metabolism appear to be most related to muscle quality (EI), with better muscle quality (i.e. lower EI) being associated with greater PS and flux. Protein intake may be particularly important during peri-menopause to stimulate protein turnover, promote a positive net balance, and the retention of LM and muscle quality.

**Acknowledgments**: This study was supported by the UNC Center for Women’s Health Research.

**Paraxanthine Provides Greater Improvement in Cognitive Function and Psychomotor Vigilance Prior to and Following Running Than Caffeine**

Dante Xing^a^, Choongsung Yoo^a^, Drew Gonzalez^a^, Tori Jenkins^a^, Kay Nottingham^a^, Broderick Dickenson^a^, Megan Leonard^a^, Joungbo Ko^a^, Megan Humphries^a^, Ryan Sowinski^a^, Christopher J. Rasmussen^a^, Richard B. Kreider^a^, Mark Faries^a,b^, Wesley Kephart^c^, Martin Purpura^d^, Ralf Jäger^d^, Shawn D. Wells^e^, FISSN

^a^Exercise & Sport Nutrition Lab, Human Clinical Research Facility, Texas A&M University, College Station, TX, USA; ^b^Texas A&M AgriLife Extension, Texas A&M University, College Station, TX, USA; ^c^Department of Health Science, University of Wisconsin – Whitewater, Whitewater, WI, USA; ^d^Increnovo LLC, Milwaukee, WI, USA; ^e^Ingenious Ingredients L.P., 2560 King Arthur BLVD Suite 124-74, Lewisville, TX, USA

**Background**: Paraxanthine (PX,1,7-dimethylxanthine) is a natural dietary component and the main metabolite of caffeine in humans. Acute ingestion of PX improves some measures of cognition, executive function, and vigilance. The purpose of this study was to compare the effects of caffeine (CA), PX, and the combined ingestion of PX and CA prior to endurance exercise on pre- or post-exercise cognition, executive function, and/or vigilance in trained runners.

**Methods**: In a double-blind, placebo controlled, crossover, and counterbalanced manner, 13 trained runners (27.1 ± 5 years, 68.6 ± 9 kg, 22.2 ± 2.7 kg/m^2^, 15.7 ± 5% fat, 53.7 ± 11 ml/kg/min VO_2peak_) were randomly assigned to consume 400 mg of placebo (PL); 200 mg of PL + 200 mg of CA; 200 mg of PL+ 200 mg of PX (ENFINITY™, Ingenious Ingredients); or, 200 mg CA + 200 mg of PX (CA+PX) with a 7-day washout between treatments. Participants donated fasting blood samples and completed pre-supplementation (PRE) side effects questionnaires (SE); the Berg Card Sorting task test (BCST) that assesses long thought, including reasoning, learning, executive control, and attention shifting; and, the Psychomotor Vigilance Task Test (PVTT) that assesses sustained attention reaction times through responses to visual stimuli. Participants then rested for 60-min, repeated tests (PRE-EX), performed a 10-km run on a treadmill (48.4 ± 6.7 min) and then repeated tests (POST-EX). Data were analyzed using General Linear Model (GLM) univariate analyses with repeated measures using weight as a covariate and mean and percent changes from baseline with 95% confidence intervals.

**Results**: GLM analysis revealed no significant treatment x time interaction effects in variables assessed using weight as a covariate although moderate effect sizes (ES) and significant pairwise differences were observed among several variables. BCST analysis revealed moderate interaction ES in Error Rate (η_p_^2^ = 0.07) with a significant difference (*p* = 0.03) observed in between PX and CA+PX PRE-RUN values as well as Perseverative Errors following PAR rules (η_p_^2^ = 0.07) with participants in the PX treatment observing a 12% reduction (*p* = 0.03) in errors from PRE-EX to POST-EX while error rate increased by 22% with CA (*p* = 0.03). Analysis of PVTT data revealed medium ES in Trial 2 (η_p_^2^ = 0.09), Trial 10 (η_p_^2^ = 0.06), Trial 20 (η_p_^2^ = 0.06), and Mean (η_p_^2^ = 0.09) Vigilance Reaction Times. Pairwise comparison revealed that PRE-RUN Mean Reaction Time was significantly faster (*p* = 0.02) in the PX treatment compared to PL and that Trial 2 POST-RUN 2-Letter Length Reaction time in the PX treatment was significantly faster than PL (*p* = 0.03). Moreover, POST-EX vigilance during trial 20 in the PX treatment tended to be different than PL (p = 0.07) and CA. In terms of safety, one participant withdrew after ingesting the CA treatment while others reported minimal side effects with no significant treatment x time effects observed in the frequency or severity of headaches, dizziness, racing heart rate, palpitations, shortness of breath, nervousness, blurred vision, or clinical chemistry panels.

**Conclusions**: Results suggest that PX supplementation is safe and improves prefrontal cortex function, attenuates attention, and mitigates cognitive fatigue prior to and following exercise. PX showed greater improvements than CA independently, while adding CA to PX did not provide any additional benefit.

**Acknowledgments**: This study was funded as a fee for service project to the Human Clinical Research Facility research by Ingenious Ingredients, LP (ING2). RJ, SDW and MP are co-owners of ING2 and have been named as inventors for the use of PX by ING2.

**A Randomized, Controlled, Double-Blind, Two-Part, Two-Period Crossover Study to Evaluate the Pharmacokinetics of Caffeine and Its Metabolites Versus d9-caffeine and Its Metabolites in Healthy Male and Female Subjects**

Bradford C. Sippy^a^, Paul M. Tarantino PhD^a^, Mary M. Sherman PhD^a^

^a^Lennham Pharmaceuticals, Inc. Concord, MA, USA

Corresponding author: brad.sippy@lennham.com

**Background**: Substitution of deuterium for hydrogen may alter the pharmacokinetics of molecules. This study was conducted to compare the single-dose pharmacokinetics of caffeine versus d9-caffeine, and their corresponding metabolites, at two dose levels in healthy adult volunteers and to characterize the exposure to d9-caffeine in slower versus rapid metabolizers of caffeine.

**Methods**: This was a randomized, controlled, double-blind, two-part, two-period crossover study. Unlabeled caffeine or d9-caffeine at two dose levels, 50 mg and 250 mg of caffeine and matching molar equivalent of d9-caffeine, was administered in a 6 oz. solution of water with blood samples collected before and up to 48 hours after administration. Plasma levels for caffeine, d9-caffeine and their respective primary metabolites were analyzed using a validated liquid chromatography with tandem mass spectrometry method.

**Results**: d9-Caffeine and caffeine were both rapidly absorbed with a similar Tmax. In the 50 mg comparison, d9-caffeine produced a 28% higher Cmax and a fourfold greater Area Under the Curve (AUC0-48 h) than a similar dose of caffeine. The three active metabolites of d9-caffeine demonstrated a lower Cmax and lower AUC0-48 h relative to their undeuterated counterparts. Slower metabolizers of caffeine demonstrated a lower relative increase in exposure to d9-caffeine compared to rapid metabolizers of caffeine. Results were consistent in the low- and high-dose comparisons. Both products were well tolerated. No adverse events for insomnia were reported during the study.

**Conclusions**: d9-caffeine exhibited a higher Cmax and substantially higher AUC0-48 h than caffeine, with lower exposure to the major metabolites of caffeine. Slower metabolizers of caffeine do not appear to be at risk of excessive exposure to d9-caffeine. d9-caffeine is potentially a safe and well-tolerated alternative to caffeine with sustained exposure from a single dose and avoids the dramatic drop in plasma concentration associated with caffeine.

**Acknowledgments**: B. Sippy is the founder of Lennham Pharmaceuticals and owns stock in the company. P Tarantino and M Sherman are consultants to Lennham Pharmaceuticals. Study conducted by Bio-Kinetic Clinical Applications, LLC, Springfield, MO, and analytical services provided Bioanalytical Services, Newark, DE, both subsidiaries of QPS LLC. This research was supported by Lennham Pharmaceuticals, Inc. Concord, MA, USA

**The Effects of 28 Days of Supplementation with Phosphocreatine Disodium Salts Plus Blueberry Extract Versus a Placebo on the Average Power Output During Maximal, Unilateral, Isokinetic Leg Extensions**

Robert W. Smith^a^, John Paul V. Anders^a^, Tyler J. Neltner^a^, Joshua L. Keller^b^, Terry J. Housh^a^, Richard J. Schmidt^a^, Glen O. Johnson^a,^ Joseph F. Daugherty^c^, Michael S. Tempesta^c^, Alekha K. Dash^d^, Daniel J. Munt^d^, FISSN

^a^Department of Nutrition and Human Sciences, University of Nebraska-Lincoln, Lincoln, NE, USA; ^b^Department of Health, Kinesiology and Sport, University of South Alabama, Mobile, AL, USA; ^c^Phenolics LLC, Omaha, NE, USA; ^d^Department of Pharmacy Sciences, School of Pharmacy and Health Professions, Omaha, NE, USA

Corresponding author: bsmith80@huskers.unl.edu

**Background**: Creatine monohydrate supplementation is known to improve muscle strength, power, and endurance. Few studies, however, have examined the effects of alternative creatine formulations on exercise performance. During exercise, the production of reactive oxygen species within the muscle has been associated with muscle fatigue. Studies have shown that supplementation with antioxidants may reduce the detrimental effects of reactive oxygen species and promote faster recovery. Polyphenols function as antioxidants and polyphenol supplementation may improve exercise performance. Therefore, the purpose of this study was to examine the effects of 28 days of supplementation with phosphocreatine disodium salts plus blueberry extract (PCDSB) versus a placebo (PLA) on the average power output during maximal, unilateral, isokinetic leg extensions.

**Methods**: Twenty-one adult men (mean ± SD: age = 20.9 ± 2.4 yrs; height = 180.9 ± 6.3 cm; body mass = 85.4 ± 12.8 kg) volunteered and were randomly assigned to consume either PCDSB (n = 11) or PLA (n = 10) for 28 days. The supplements were designed to be similar in volume, color, and taste. The PCDSB included 5.0 g of phosphocreatine disodium salts plus 200 mg of blueberry extract and the PLA included microcrystalline cellulose. Before and after the 28 days of supplementation, the subjects performed three maximal, unilateral (left leg), isokinetic leg extensions at 180°·s^−1^ and the average power output was defined as the mean power output from the three repetitions. The average power output data were analyzed with a 2(Time: Pre-Supplementation vs. Post-Supplementation) x 2(Group: PCDSB vs. PLA) mixed factorial ANOVA. Follow-up pairwise comparisons and post-hoc comparisons were used when necessary.

**Results**: The results of the mixed factorial ANOVA indicated a significant (*p* = 0.048, *η^2^_р_* = 0.190) Time x Group interaction. Follow-up pairwise comparisons demonstrated that average power output increased (*p* = 0.001, *d* = 0.793) following supplementation with PCDSB (140.18 ± 32.08 vs. 170.12 ± 42.68 W), but the PLA did not (*p* = 0.279, *d* = 0.151; 141.94 ± 51.92 vs. 150.79 ± 64.30 W)

**Conclusions**: Supplementation for 28 days with PCDSB, but not the PLA, resulted in improved average power output during maximal, unilateral, isokinetic, leg extensions. Individuals with the goal of improving power output may benefit from supplementation with phosphocreatine disodium salts plus blueberry extract. Further studies, however, on the effects of supplementation with alternative creatine formulations are warranted.

**Acknowledgments**: There are no conflicts of interest.

**The Effects of Nutrition Intervention Through a Social Media Platform (Instagram) on Nutrition Knowledge and CVD Risk Factors Amongst Fire-Fighters**

Katie Emerson^a^, Douglas Kalman^a,b^, Michael Downing^c^, FISSN

^a^Nutrition Department. College of Osteopathic Medicine, Nova Southeastern University, Davie, FL, USA; ^b^Nutrasource Pharmaceutical and Nutraceutical Services, Scientific Affairs, Guelph, Ontario, Canada; ^c^Osteopathic Medicine Department. College of Osteopathic Medicine, Nova Southeastern University, Davie, FL, USA

Corresponding author: Katiesimons5@gmail.com

**Background**: Cardiovascular Disease (CVD) is the number one cause of on-duty death for firefighters. Risk factors such as hypertension, obesity, and cholesterol can be used as biomarkers in the prevention and intervention of CVD. Social media has become a popular platform for delivering health and wellness information. Although some studies have measured the feasibility of social media-based interventions on health behaviors, none have examined the efficacy of using social media to measure changes in nutrition knowledge and health in fire-fighters. Fire-fighters typically are physically active on a daily basis, however, appear to have greater risks of CVD. The aims of this study are to examine the utility of nutrition education through a social media platform (Instagram) for supporting and enhancing nutrition knowledge and in reducing CVD risk factors amongst firefighters.

**Methods**: This experimental study operated as a pilot program. The aim of this study was to determine if nutrition content delivered through Instagram (IG) would have any impacts on health and nutrition knowledge status of the firefighters. Fifty-three firefighters signed informed consent and entered the study. The validated Abridged Nutrition for Sport Knowledge Questionnaire (ANSKQ) was administered before and after the 6-week intervention period. A private locked IG account was created as a means of delivering daily evidence-based nutrition information from reputable organizations daily. Nutrition knowledge, anthropometric and vital data were collected pre-post intervention. Data analysis is presented with the mean, ± SD, and range, paired T-tests were used (two-tailed) for the data analysis.

**Results**: Pre-post ASNKQ results were evaluated by total, general (GNK) and sports nutrition knowledge (SNK) scores. The total mean score increased significantly from 46% ±13.27 to 52% ± 13.43 (p < 0.0017). When looking at the sub-categories, a 5% increase in GNK scores was observed with a pre-intervention mean score of 60% ± 15.35 to 65% ± 19.83 (p < 0.04409). The SNK scores also increased by 6% with a pre-intervention mean score of 39% ± 16.01 to 45% ±14.25 (p < 0.0108). By the end of the 6-week program, participants lost an average of 1.54 kg ± 2.29 (p < 0.00007). No significant changes were observed in blood pressure post-intervention.

**Conclusions**: This study suggests that nutrition education delivered via social media can have an impact on nutrition knowledge and health behaviors. This appears so by promoting greater nutrition knowledge, weight control guidance and positive lifestyle behavior changes. As this was a pilot study, more research is needed to validate this form of nutrition and health education. Evidence-based nutrition and health education delivered over social-media can be a beneficial tool for health enhancement of fire-fighters.

**Acknowledgments**: No conflicts or sponsorships to report

**Keywords**: Nutrition knowledge, Sports nutrition, Questionnaire, Social media, Instagram, Pilot study, Review, Efficacy, Cardiovascular Disease, Fire fighter, first responder, CVD risk factors

**A Combination of Teacrine, Dynamine, and Caffeine Increases Mental Performance Without Increasing Anxiety in E-Gamers**

Frankie Pizzo^a^, Daisy Valle, Mykola Marang^a^, Jaime Tartar^a^, Amanda Pultorak^b^, Olivia Longmore^b^, Talia Thompson^b^, Anna Santucci^b^, Haleigh Adams^b^, Jose Antonio^b^, FISSN

^a^College of Psychology, Nova Southeastern University, 3301 College Ave, Ft Lauderdale, FL, USA; ^b^College of Health Sciences, Nova Southeastern University, 3301 College Ave, Ft Lauderdale, FL, USA

Corresponding author: tartar@nova.edu

**Background**: Caffeine in the range of 32-300 mg improves measures of neurobehavioral performance. However, high doses of caffeine are associated with unwanted side-effects such as increased tension and symptoms of anxiety, nervousness, and jitteriness. Accordingly, the current study sought to determine if caffeine combined with a non-caffeine adjunctive supplement (Caffeine + Teacrine + Dynamine) could increase neurobehavioral performance without increasing unwanted side effects in e-gamers.

**Methods**: We carried out an assessment of the effects of an acute, single-dose treatment of Caffeine (125 mg) vs. Caffeine (125 mg) + TeaCrine (50 mg) + Dynamine (75 mg) vs. Matched Placebo. This study used a randomized double-blinded, crossover design. Each participant was tested under all three conditions 1 week apart. Participants consisted of n = 50 male amateur e-gamers who play a first-person video game for at least 10 hrs a week. Neurobehavioral performance was assessed through a series of cognitive testing instruments from the NIH toolbox and the PVT from Joggle Research. Neurophysiological arousal was captured through a single-channel EEG (Enchanted Wave, LLC) with the electrode positioned at the prefrontal-lobe location (Fp1). Biomarkers of arousal included Salivary Alpha Amylase (sAA) and cortisol (Salimetrics, Carlsbad, CA).

**Results**: Compared to baseline (pre-dose) performance, both Caffeine (p = 0.001) and Caffeine/TeaCrine/Dynamine (CTD) (p = 0.002) improved attention and inhibitory control as measured via the Flanker Task. Caffeine and CTD differentially affected delta activity during the cognitive tasks. CTD increased delta power relative to baseline (p = 0.05) while caffeine decreased delta power (p = 0.04). Only CTD increased theta activity during the attention task relative to baseline (p = 0.04). Caffeine increased self-reported anxiety relative to the placebo condition (p = 0.03). Compared to placebo, CTD also significantly increased cortisol levels post-dose before (p = 0.005) and after (p = 0.018) cognitive testing. Compared to caffeine, CTD also increased cortisol at post-dose, pre-cognition testing (p = 0.01).

**Conclusions**: Both caffeine and CTD resulted in improved performance on inhibitory attention and control.

However, only caffeine significantly increased anxiety post-dose compared to placebo. The EEG changes suggest that the CDT condition increased attention to internal processing (decreased delta) and increased cognitive control (increased theta). The increase in cortisol in the CTD group possibly reflects increased energy mobilization with this treatment.

**Acknowledgments**: This research was funded by Compound Solutions Inc. The funder played no role in data acquisition, data management, nor data interpretation.

**Bioavailability of Berberine and Dihydroberberine and Their Impact on Glycemia: A Randomized Controlled, Crossover Pilot Trial**

Jessica M Moon^a^, Richard A. Stecker^a^, Kayla M. Ratliff^a^, Chad M. Kerksick^a^, FISSN

^a^Exercise and Performance Nutrition Laboratory, School of Health Sciences, Lindenwood University, St. Charles, MO, USA

Corresponding author: ckerksick@lindenwood.edu

**Background**: Berberine (BBR) is a natural alkaloid used to improve glycemia, but displays poor bioavailability and increased rates of gastrointestinal distress at higher doses. Recently, dihydroberberine has been developed to combat these challenges. This study was designed to determine the bioavailability of a 500 mg dose of BBR (B500) and 100 mg and 200 mg doses of Dihydroberberine (D100 and D200).

**Methods**: In a randomized, double-blind, crossover fashion, five males (26 ± 2.6 yrs; 184.2 ± 11.6 cm; 91.8 ± 10.1 kg; 17.1 ± 3.5%BF) completed a four-dose supplementation protocol of placebo (PLA), B500, D100, and D200. Prior to their scheduled visit, participants ingested three separate doses of breakfast, lunch, and dinner. Participants fasted overnight (8–10 hours) and consumed their fourth dose with a standardized test meal (30 grams glucose, 3 slices white bread) after arrival. Venous blood samples were collected 0, 20, 40, 60, 90, and 120 minutes after ingestion and analyzed for BBR, glucose, and insulin. Peak concentration (C_Max_) and area under the curve (AUC) were calculated for all variables.

**Results**: Baseline BBR levels were different between groups (p = 0.006), with pairwise comparisons indicating that baseline levels of PLA and B500 were different than D100. BBR C_Max_ tended to be different (p = 0.06) between all conditions. Specifically, D100 (3.8 ± 1.4 ng/mL) was different than PLA (0.22 ± 0.18 ng/mL, p = 0.03) and B500 (0.4 ± 0.17 ng/mL, p = 0.03). Significant differences in BBR AUC were found between D100 (284.4 ± 115.9 ng/mL•120 min) and PLA (20.2 ± 16.2 ng/mL•120 min, p = 0.04) and B500 (42.3 ± 17.6 ng/mL•120 min, p = 0.04). Significant differences in BBR AUC were found between D100 (284.4 ± 115.9 ng/mL•120 min) and PLA (20.2 ± 16.2 ng/mL•120 min, p = 0.04) and B500 (42.3 ± 17.6 ng/mL•120 min, p = 0.04). No significant differences in the levels of glucose (p = 0.97) and insulin (p = 0.24) were observed across the study protocol.

**Conclusion**: These results provide preliminary evidence that four doses of D100 and D200 produce significantly higher concentrations of berberine within the bloodstream two hours after ingestion when compared to B500 or PLA. The lack of observed changes in glucose and insulin were likely due to the short duration of supplementation and insulin responsive nature of study participants. Follow-up efficacy studies on glucose and insulin changes should be completed to assess the impact of berberine and dihydroberberine supplementation in overweight, glucose intolerant populations.

**Funding**: This study was supported by an unrestricted grant from NNB Nutrition.

**Disclosure**: Dr. Kerksick serves as a paid advisor for NNB Nutrition. Dr. Kerksick was not involved in any part of the consenting, randomization, or data collection process. All statistical analysis was completed prior to unblinding. A conflict of interest management plan was filed and approved by the Lindenwood University IRB prior to commencing any data collection.

**Characterization of Cardiac and Hemodynamic Responses to Blood Flow Restriction in Women**

Abby R. Fleming^a^, Lee J. Winchester^a^, FISSN

^a^LAB INSERT, Department insert, University of Alabama, Tuscaloosa, AL, USA

Corresponding Author: arfleming2@crimson.ua.edu

**Background**: Blood Flow Restriction (BFR) is newly popularized because it is able to create the hypoxic environment seen while exercising at high intensities, but during low-intensities. Very few studies have investigated the cardiac and hemodynamic effects of BFR during rest. Research investigating the cardiovascular effects of BFR during exercise has conflicting results. The purpose of this study was to determine what physiological changes occur during two clinical BFR pressures (50% and 80% of limb occlusion pressure). Heart rate (HR), blood pressure (BP) tibial and brachial artery blood flow (BF), mean velocity (TAMV), and blood vessel diameter were analyzed.

**Figure 1.**
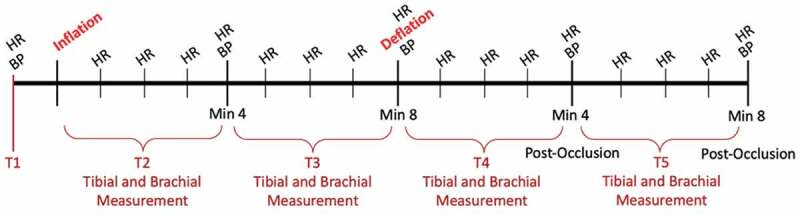
Layout of Methods.

**Methods**: Sixteen healthy women participated in this study (Table 1). Participants sat on a gurney chair with Delfi PTS cuffs on both thighs. Baseline (T1) measurements of HR, BP, BF in the brachial and posterior tibial arteries were evaluated after resting for 3 minutes. Both cuffs were inflated for 8 minutes, to either 50% or 80% of personal occlusion pressure. HR was taken every minute while BP was taken at the end of the first (T2) and last 4 minutes (T3) of inflation. Brachial and tibial artery measurements were taken at T2 and T3, at least 30 seconds after inflation. After deflation, participants remained seated for 8 minutes with no BFR, while all the measurements were re-collected at 0-4 (T4) and 4-8 (T5) minutes post-occlusion (Figure 1). Repeated measures ANOVAs were used with Bonferroni correction to determine significant changes. An alpha level of <0.05 was used to determine significance.

**Table 1.** Demographics of Participants

	Mean	SD
Age	23.1	4.9
Height	166.3cm	6.5cm
Weight	64.6kg	10.3kg
Body Fat	23.3%	7.0%

**Figure 2.**
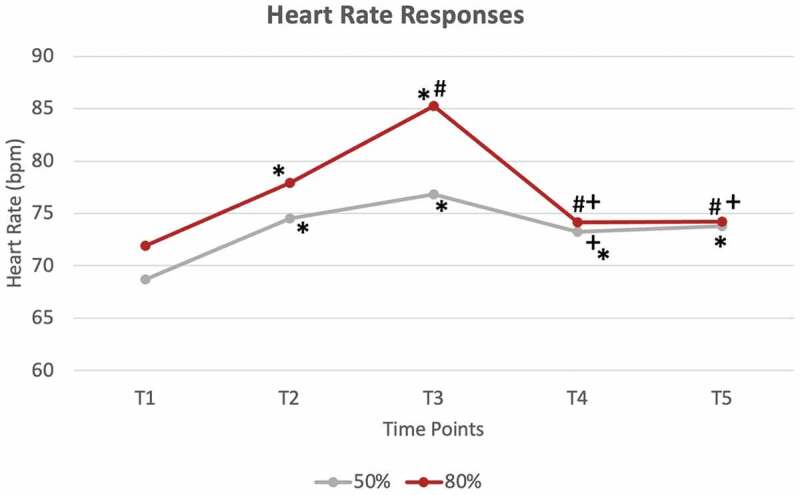
Average Heart Rate changes for both 50% occlusion pressure and 80% occlusion pressure at all time points.

**Figure 3.**
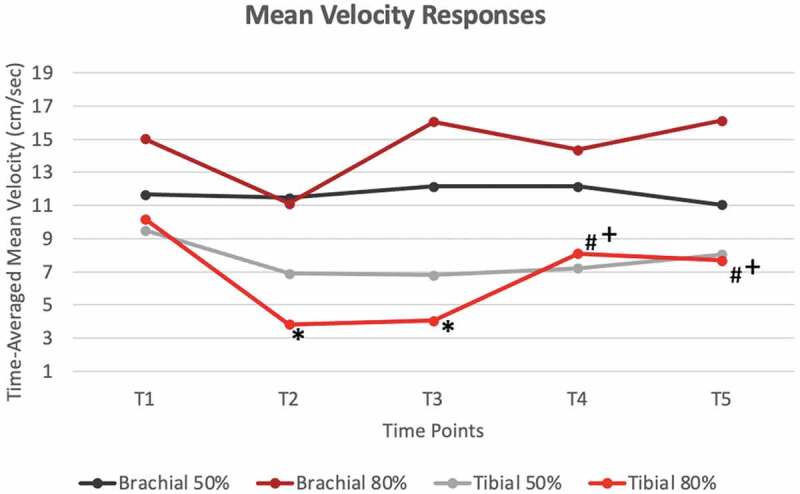
Average changes of Time-Averaged Mean Velocity in both Tibial and Brachial Arteries at both 50% occlusion pressure and 80% occlusion pressure for all time points.

**Figure 4.**
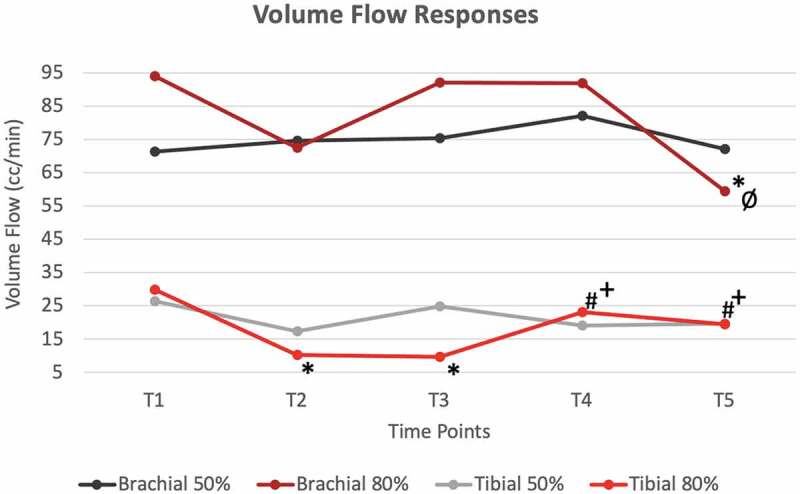
Average changes of Volume Flow in both Tibial and Brachial Arteries at both 50% occlusion pressure and 80% occlusion pressure for all time points.

**Results**: There were no significant differences for the 50% BFR pressure for any hemodynamic responses. However, HR at T1 was significantly lower than at every other time point, and HR was also higher for the T3 vs. T4 (Figure 2). Significant mean differences were observed for the 80% pressure for brachial artery BF between T1 and T5 and between T4 and T5 (Figure 4). In the tibial artery at 80% pressure, there were significant differences in TAMV between T1 vs. T2/T3, T2 vs. T4/T5 and T3 vs. T4/T5 (Figure 3). These same differences were observed with BF as well (Figure 4). Additionally, there were significant HR differences between T1 vs. T2/T3, T2 vs. T3, T4 vs. T5, T3 vs. T4/T5 (Figure 2). CONCLUSIONS: Results demonstrate that 80% of limb occlusion pressure showed significant differences in both cardiac and hemodynamic responses to BFR, while 50% pressure may only change cardiac responses.

* significantly different than T1(p<0.05)# significantly different than T2(p<0.05)+ significantly different than T3(p<0.05)

* significantly different than T1(p<0.05)# significantly different than T2(p<0.05)+ significantly different than T3(p<0.05)

* significantly different than T1(p<0.05)# significantly different than T2(p<0.05)+ significantly different than T3(p<0.05)ø significantly different than T4(p<0.05)

**Conclusions**: Results demonstrate that 80% of limb occlusion pressure showed significant differences in both cardiac and hemodynamic responses to BFR, while 50% pressure may only change cardiac responses.

**Baseline Anthropometric, Dietary intake, and Behavioral Variables Do Not Predict Study Completion or Fat Loss Success in Young, Resistance-trained Females Undergoing a Dietary Intervention**

Madelin R. Siedler^a,b^, Megan N. Humphries^b,c^, Priscila Lamadrid^b^, Gianna F. Mastrofini^b^, James Gegenheimer^b^, Adam H. Ibrahim^b^, Alexander M. Brooks^b^, Traci Smith^b^, Grant M. Tinsley^a^, & Bill I. Campbell^b^

^a^Energy Balance and Body Composition Laboratory, Texas Tech University, Lubbock, TX, USA; ^b^Performance and Physique Enhancement Laboratory, University of South Florida, Tampa, FL, USA; ^c^Muscle Biology Laboratory, Texas A&M University, College Station, TX, USA

Corresponding author: msiedler@ttu.edu

**Background**: Participant attrition and lack of adherence are commonly encountered in nutrition research. The identification of baseline variables that can predict potential attrition or adherence issues in participants may allow for targeted support of these participants throughout the intervention, which may potentially improve completion rates and outcomes. The purpose of this analysis was to assess whether baseline anthropometric (bodyweight, body mass index [BMI], body fat percentage [BF%]), dietary (energy, carbohydrate, protein, and fat intake), or behavioral variables (cognitive restraint, hunger, and disinhibition via the 51-item Three-Factor Eating Questionnaire [TFEQ]) predicted successful study completion or fat loss in young, resistance-trained, non-obese females.

**Methods**: Forty-six participants (mean ± SD age: 22.7 ± 4.4 years; height: 163.6 ± 6.7 cm; weight: 63.4 ± 8.9 kg; BF%: 25.2 ± 4.2) were included in this analysis. All participants underwent 6 total weeks of moderate (25%) energy restriction with 3 weekly supervised resistance training sessions. Thirty-five participants completed the intervention *per protocol* while 11 (23.9%) either withdrew from the study after randomization or did not adhere to the dietary intervention. Baseline values for anthropometric variables were compared between the two groups using an independent-samples *t*-test. Dietary intake and behavioral variables were compared using a Mann-Whitney U test for non-parametric data. Correlations using Pearson’s *r* or Spearman’s rho were used to assess associations between these variables at baseline and change in ultrasound fat mass in all participants for whom post-treatment data were collected (*n* = 36).

**Results**: Completers did not significantly differ from non-completers for any of the 10 variables (*p* ≥ 0.08), though differences in disinhibition scores trended toward significance (*p* = 0.08). Notably, baseline disinhibition scores were higher in non-completers (median = 8; interquartile range [IQR] = 6.5) than in completers (median = 5; IQR = 4.0), indicating that non-completers began the study with higher tendencies toward uncontrolled, opportunistic, and/or emotionally driven eating. Change in fat mass (mean change: −1.1 ± 1.1 kg) was not correlated with any of the 10 variables (*p* ≥ 0.23).

**Conclusions**: Results demonstrate that 80% of limb occlusion pressure showed significant differences in both cardiac and hemodynamic responses to BFR, while 50% pressure may only change cardiac responses.

**Bioavailability of Berberine and Dihydroberberine and their Impact on Glycemia: A Randomized Controlled, Crossover Pilot Trial**

Jessica M Moon^a^, Richard A. Stecker^a^, Kayla M. Ratliff^a^, Chad M. Kerksick^a^, FISSN

^a^Exercise and Performance Nutrition Laboratory, School of Health Sciences, Lindenwood University, St. Charles MO, 63,101, USA

Corresponding author: ckerksick@lindenwood.edu

**Background**: Berberine (BBR) is a natural alkaloid used to improve glycemia, but displays poor bioavailability and increased rates of gastrointestinal distress at higher doses. Recently, dihydroberberine has been developed to combat these challenges. This study was designed to determine the bioavailability of a 500 mg dose of BBR (B500) and 100 mg and 200 mg doses of Dihydroberberine (D100 and D200).

**Methods**: In a randomized, double-blind, crossover fashion, five males (26 ± 2.6 yrs; 184.2 ± 11.6 cm; 91.8 ± 10.1 kg; 17.1 ± 3.5 %BF) completed a four-dose supplementation protocol of placebo (PLA), B500, D100, and D200. Prior to their scheduled visit, participants ingested three separate doses of breakfast, lunch, and dinner. Participants fasted overnight (8–10 hours) and consumed their fourth dose with a standardized test meal (30 grams glucose, 3 slices white bread) after arrival. Venous blood samples were collected 0, 20, 40, 60, 90, and 120 minutes after ingestion and analyzed for BBR, glucose, and insulin. Peak concentration (C_Max_) and area under the curve (AUC) were calculated for all variables.

**Results**: Baseline BBR levels were different between groups (p = 0.006), with pairwise comparisons indicating that baseline levels of PLA and B500 were different than D100. BBR C_Max_ tended to be different (p = 0.06) between all conditions. Specifically, D100 (3.8 ± 1.4 ng/mL) was different than PLA (0.22 ± 0.18 ng/mL, p = 0.03) and B500 (0.4 ± 0.17 ng/mL, p = 0.03). Significant differences in BBR AUC were found between D100 (284.4 ± 115.9 ng/mL•120 min) and PLA (20.2 ± 16.2 ng/mL•120 min, p = 0.04) and B500 (42.3 ± 17.6 ng/mL•120 min, p = 0.04). Significant differences in BBR AUC were found between D100 (284.4 ± 115.9 ng/mL•120 min) and PLA (20.2 ± 16.2 ng/mL•120 min, p = 0.04) and B500 (42.3 ± 17.6 ng/mL•120 min, p = 0.04). No significant differences in the levels of glucose (p = 0.97) and insulin (p = 0.24) were observed across the study protocol.

**Conclusions**: These results provide preliminary evidence that four doses of D100 and D200 produce significantly higher concentrations of berberine within the bloodstream two hours after ingestion when compared to B500 or PLA. The lack of observed changes in glucose and insulin were likely due to the short duration of supplementation and insulin responsive nature of study participants. Follow-up efficacy studies on glucose and insulin changes should be completed to assess the impact of berberine and dihydroberberine supplementation in overweight, glucose intolerant populations.

**Funding**: This study was supported by an unrestricted grant from NNB Nutrition.

Disclosure: Dr. Kerksick serves as a paid advisor for NNB Nutrition. Dr. Kerksick was not involved in any part of the consenting, randomization, or data collection process. All statistical analysis was completed prior to unblinding. A conflict-of-interest management plan was filed and approved by the Lindenwood University IRB prior to commencing any data collection.

**The Effects of Nutrition Intervention Through a Social Media Platform (Instagram) on Nutrition Knowledge and CVD Risk Factors Amongst Fire-Fighters**

Katie Emerson^a^, Douglas Kalman^a,b^, Michael Downing^c^

^a^Nutrition Department, College of Osteopathic Medicine, Nova Southeastern University, Davie, FL, USA; ^b^Nutrasource Pharmaceutical and Nutraceutical Services, Scientific Affairs, Guelph, Ontario-Canada; ^c^Osteopathic Medicine Department. College of Osteopathic Medicine, Nova Southeastern University, Davie, FL, USA

Corresponding author: Katiesimons5@gmail.com

**Background**: Cardiovascular Disease (CVD) is the number one cause of on-duty death for firefighters. Risk factors such as hypertension, obesity, and cholesterol can be used as biomarkers in the prevention and intervention of CVD. Social media has become a popular platform for delivering health and wellness information. Although, some studies have measured the feasibility of social media-based interventions on health behaviors, none have examined the efficacy of using social media to measure changes in nutrition knowledge and health in fire-fighters. Fire-fighters typically are physically active on a daily basis, however, appear to have greater risks of CVD. The aims of this study are to examine the utility of nutrition education through a social media platform (Instagram) for supporting and enhancing nutrition knowledge and in reducing CVD risk factors amongst firefighters.

**Methods**: This experimental study operated as a pilot program. The aim of this study was to determine if nutrition content delivered through Instagram (IG) would have any impacts on health and nutrition knowledge status of the firefighters. Fifty-three firefighters signed informed consent and entered the study. The validated Abridged Nutrition for Sport Knowledge Questionnaire (ANSKQ) was administered before and after the 6-week intervention period. A private locked IG account was created as a means of delivering daily evidence-based nutrition information from reputable organizations daily. Nutrition knowledge, anthropometric and vital data were collected pre-post intervention. Data analysis is presented with the mean, ± SD, and range, paired T-tests were used (two-tailed) for the data analysis.

**Results**: Pre-post ASNKQ results were evaluated by total, general (GNK) and sports nutrition knowledge (SNK) scores. The total mean score increased significantly from 46% ±13.27 to 52% ± 13.43 (p < 0.0017). When looking at the sub-categories, a 5% increase in GNK scores was observed with a pre-intervention mean score of 60% ± 15.35 to 65% ± 19.83 (p < 0.04409). The SNK scores also increased by 6% with a pre-intervention mean score of 39% ± 16.01 to 45% ±14.25 (p < 0.0108). By the end of the 6-week program, participants lost an average of 1.54 kg ± 2.29 (p < 0.00007). No significant changes were observed in blood pressure post-intervention.

**Conclusions**: This study suggests that nutrition education delivered via social media can have an impact on nutrition knowledge and health behaviors. This appears so by promoting greater nutrition knowledge, weight control guidance and positive lifestyle behavior changes. As this was a pilot study, more research is needed to validate this form of nutrition and health education. Evidence-based nutrition and health education delivered over social-media can be a beneficial tool for health enhancement of fire-fighters.

**Acknowledgments**: No conflicts or sponsorships to report

**Keywords**: Nutrition knowledge, Sports nutrition, Questionnaire, Social media, Instagram, Pilot study, Review, Efficacy, Cardiovascular Disease, Fire fighter, first responder, CVD risk factors

**A Nutritional Intervention to Attenuate Upper Respiratory Tract Infections in Collegiate Distance Runners**

Emily Newberry^a^, Candice Thomas, PhD^a^

^a^University of Central Arkansas, Conway, AR, USA

Corresponding author: enewberry1@cub.uca.edu

**Background**: We aimed to determine if the consumption of berries can reduce URTI incidence in collegiate distance runners. Upper respiratory tract infection (URTI) incidence is elevated in endurance athletes due to immune suppression that accompanies performance of intense, exercise. Since berries have a high content of immune-enhancing compounds such as vitamin C, antioxidants, and polyphenols, we hypothesized that URTI incidence would be reduced in the study population after long-term consumption of berries due to an improved immune response, as quantified by increased levels of salivary Immunoglobulin A (SIgA) and lower salivary cortisol. SIgA is the main marker of mucosal immunity, while cortisol was measured to determine the participants’ overall stress response and how it was affected by berry consumption.

**Methods**: 14 trained collegiate distance runners completed a weekly survey detailing their running mileage, illness symptoms, and dietary intake throughout their cross-country and track seasons, then gave saliva samples to determine their levels of SIgA and cortisol. During the control phase (n = 14) (cross-country season), the athletes consumed their normal diet. During the intervention phase (n = 8) (track season) athletes were asked to add 2 cups of raw or frozen berries to their daily diet. After completion of the intervention phase, an ELISA of SIgA and cortisol was performed.

**Results**: In both control and intervention phases, higher weekly running mileage was positively correlated with illness symptoms and illness interference with activities of daily living (ADLs). The elevation and slope of the correlation between weekly running mileage, illness symptoms, and interference with ADLs was greater in the control phases than the intervention phase. Perceived illness was negatively correlated with weekly running mileage during the control phases and positively correlated during the intervention phase. SIgA levels were highest in the 40-60 mi group and greater during the control phase than during the intervention phase. Salivary cortisol levels were highest for the 60-80 mi group during the control phase, but lowest for the same group during the intervention phase.

**Conclusions**: While preliminary, our research suggests that berry consumption may impact markers of host immunity based on survey data and salivary cortisol levels. The berry intervention may have helped to lower cortisol, and thus the participants’ stress response to high-intensity training, suggesting that berry consumption may help strengthen the immune system to better prevent URTI. Our preliminary survey data also support this conclusion since illness symptoms and interference with ADLs appear to have increased less with higher weekly running mileage during the control phase than during the intervention phase. Supplementation for 28 days with PCDSB, but not the PLA, resulted in improved average power output during maximal, unilateral, isokinetic, leg extensions. Individuals with the goal of improving power output may benefit from supplementation with phosphocreatine disodium salts plus blueberry extract. Further studies, however, on the effects of supplementation with alternative creatine formulations are warranted.

**Acknowledgments**: There are no conflicts of interest.

**Teaching Nutrition Concepts Using Pop Culture: Why Zombies Crave Brains**

Greg E Popovich^a^, Kristy D Henson^b^

^a^School of Exercise Science & Athletic Training, West Virginia Wesleyan College, Buckhannon, WV, 26,201, USA; ^b^School of Science & Technology, Fairmont State University, Fairmont, WV, 26,554, USA

Corresponding author: popovich.g@wvwc.edu

**Background**: Recently, the allure of zombie culture has found its way into innovative teaching of higher education. In the current example, zombies are used to facilitate understanding of applied nutritional biochemistry. Our purpose is to evaluate the benefit of incorporating zombie lore into the delivery of a lecture on the inflammatory cascade as it relates to the essential fatty acids. We hypothesize that–given the popularity of zombies and dystopian/apocalyptic environments–intertwining nutritional concepts with these scenarios will enhance interest and attention, hence improving learning.

**Methods**: Two presentations on essential fatty acids were provided: one including zombie references and one without. The presentations were offered in two general education courses: nutrition and anatomy and physiology (n = 73). Half of the students received a normal lecture while half received the same information but as it relates to zombies. Learning was assessed using a five-question pretest and posttest. Students were dissuaded from taking notes to assure that the zombie group would receive ample exposure to zombie graphics and examples. In both instances, students were encouraged to not worry about the test but rather to enjoy the class.

**Results**: Findings were as follows: the pooled pretest average for both groups was 44.4%; the posttest average was 86.1%. The mean score increase pre- versus posttest in both groups was 97.8% non-zombie and 90.7% zombie. Pre and posttest results for each lecture were compared using a t-test showing significance: non-zombie p = 2.2^−12^, zombie p = 4.8^−8^. Zombie and non-zombie test scores were compared by ANOVA to determine significance between lectures (p = 0.998). Performance improvements between zombie and non-zombie groups were statistically indistinguishable. The majority of students (58.9%) indicated a moderate or higher level of interest in zombies; 82.4% had an interest in nutrition; 52% had previous nutritional knowledge; and 83.5% were underclassmen. Test scores and demographic data were compared using a PCA but did not suggest any meaningful correlations.

**Conclusions**: Dramatic improvements in short-term learning occurred in both groups. Performance improvement between the zombie and non-zombie groups was statistically indistinguishable. Inclusion of zombie references was neither a benefit nor a detriment to student learning. It is possible that exposure to the pretest primed students for the posttest, thereby enhancing learning. Although this investigation did not indicate that pop culture allusions improve short-term learning, it may suggest that an instructor has the freedom to include tangential information that the instructor finds enjoyable.

**The Effects of Phosphocreatine Disodium Salts Plus Blueberry Extract Supplementation on Muscular Strength**

John Paul V. Anders^a^, Tyler J. Neltner^a^, Robert W. Smith^a^, Joshua L. Keller^b^, Terry J. Housh^a^, F. Joseph Daugherty^c^, Michael S. Tempesta^c^, Alekha K. Dash^d^, Daniel J. Munt^d^, Richard J. Schmidt^a^, Glen O. Johnson^a^

^a^Department of Nutrition and Human Sciences, University of Nebraska-Lincoln, Lincoln, NE 68510, USA; ^b^Department of Health, Kinesiology and Sport, University of South Alabama, Mobile, AL 36688, USA; ^c^Phenolics LLC, Omaha, NE 68144, USA; ^d^Department of Pharmacy Sciences, School of Pharmacy and Health Professions, Omaha, NE 68178, USA

Corresponding author: janders@huskers.unl.edu

**Background**: The efficacy of creatine supplementation for improvements in exercise performance has been well established in the literature. Few studies, however, have examined the effects of phosphocreatine supplementation on exercise performance. Furthermore, polyphenols, a micronutrient found in blueberries, exhibit antioxidant and anti-inflammatory properties, however, little is known regarding the influence of polyphenol supplementation on muscular strength. Thus, the purpose of the present study was to compare the effects of 28 days of supplementation with phosphocreatine disodium salts plus blueberry extract (PCDSB), creatine monohydrate (CM), and placebo on measures of muscular strength.

**Methods**: Thirty-three men (Mean ± SD; age = 20.2 ± 1.1 years; weight = 85.5 ± 16.1 kg; height = 180.3 ± 5.5 cm) were randomly assigned to consume either PCDSB (5.0 g of phosphocreatine and disodium salts plus 200 mg of blueberry extract), CM (3.0 g of creatine monohydrate), or placebo (microcrystalline cellulose) for 28 days. Peak torque (PT) was defined as the average of three maximal, unilateral (left leg), isokinetic leg extensions at 180°·s^−1^ and was assessed before and after the 28 days of supplementation. The data were examined with a 3 (Group) by 2 (Time) mixed factorial ANOVA and planned pairwise comparisons. Individual responses for PT were assessed to examine the proportion of subjects that exceeded a minimal important difference (MID). The MID was calculated as the product of the pooled pre-supplementation standard deviation for PT and 0.5, which reflects a moderate effect size. The proportional differences between supplement groups were examined with chi-squared tests.

**Results**: The mixed factorial ANOVA demonstrated no significant interaction or main effect for Group, but a significant (*p* < 0.05) main effect for Time. The planned pairwise comparisons demonstrated significant (*p* < 0.05) improvements in PT for the PCDSB and CM groups from pre- (99.90 ± 22.47 N·m and 99.95 ± 22.50 N·m, respectively) to post-supplementation (119.22 ± 29.87 N·m and 111.97 ± 24.50 N·m, respectively), but no significant (*p* = 0.112) change for the placebo group. A significantly (*p* < 0.05) greater proportion of subjects in the PCDSB group exceeded the MID for PT compared to the placebo group, but there was no significant (*p* > 0.05) difference in the proportion of subjects that exceeded the MID between the CM and placebo group.

**Conclusions**: These findings indicated that for the group mean responses, 28 days of supplementation with both PCDSB and CM resulted in increases in PT. The PCDSB, however, may have an advantage over CM when compared to the placebo group for the proportion of individuals that respond favorably to supplementation with meaningful increases in muscular strength.

**An Evaluation of Botanical Blend Dietary Supplement on Cognitive and Physical Performance**

Jaime L. Tartar^a^, Jose Antonio^b^, Douglas Kalman^a,b,c^, Susan Joyce Hewlings^c,d^, Joshua Baisley^c^, Mykola Marang^a^, Sarah M. Flynn^e^ and Corey A. Peacock^b^

^a^College of Psychology, Nova Southeastern University, Ft Lauderdale, FL, 33,314, USA; ^b^College of Health Sciences, Nova Southeastern University, Ft Lauderdale, FL, 33,314, USA; ^c^Nutrasource Pharmaceutical and Nutraceutical Services Inc., 120 Research Lane, Suite 101, Guelph, ON, Canada; ^d^The Herbert H & Grace A. Dow College of Health Professions, Nutrition, Central Michigan University, Mt. Pleasant, MI 48859, USA; ^e^Glanbia Nutritionals, Glanbia Research and Development Center, Twin Falls, ID, USA

Corresponding author: tartar@nova.edu

**Background**: There is considerable interest in safe and effective nutritional supplements that can improve mental and physical performance. A large and growing body of research shows that non-caffeinated plant-based nutritional supplements can increase cognitive and physical performance. This study aimed to build on this work by investigating the possibility that a specific botanical blend comprised of *Bacopa monnieri* bacosides, *Kaempferia parviflora* methoxy flavones, pomegranate peel polyphenols, and Moringa oleifera leaf saponins) could improve cognitive and physical performance.

**Methods**: This was a randomized, double-blind, placebo-controlled 21-day parallel study. The study evaluated the effects of a botanical blend compared to green tea caffeine extract and a placebo on cognitive and physical performance in healthy, physically active adults. Testing occurred on Days 1, 7, 14, and 21. The botanical blend was composed of Bacopa monnieri extract, moringa saponins and polyphenols and Methoxyflavones derived from both pomegranate peel and black ginger extracts. Neurobehavioral measures included the Mood, Alertness, and Physical Sensation Scale (MAPSS) along with a standard cognitive test battery through the National Institutes of Health (NIH) Toolbox and a Psychomotor Vigilance Task (Joggle, LLC, USA). Salivary biomarkers included measures of salivary Alpha amylase (sAA) and cortisol which were quantified via EIA and ELISA, respectively, according to manufacturer instructions (Salimetrics, LLC, USA). Physical performance was assessed through a standard Time to Exhaustion Test with simultaneous heart rate recording.

**Results**: We found that relative to baseline and compared to the Caffeine and Placebo groups, the botanical blend increased alertness (p < 0.05) and improved cognitive performance on measures of psychomotor vigilance, attentional control, and dimensional change at various time points in the study (all p’s < 0.05). The botanical blend did not improve physical performance on a time to exhaustion (TTE) test.

**Conclusions**: The botanical blend increased alertness and multiple measures of cognitive performance. The effects were more robust with longer use and for attention measures. These improvements in alertness and cognitive performance were not accompanied by an increase in ‘jitters’, as measured by errors on the psychomotor vigilance task (PVT). The botanical blend did not improve physical performance on a time to exhaustion (TTE) test either acutely or after 21 days of supplementation.

**Acknowledgments**: This research was funded by Glanbia Nutritionals, Twin Fall ID. Sarah Flynn is an employee of Glanbia, the study funder. All other authors declare no conflicts of interest as related to this study. The funder played no role in data acquisition, data management, nor data interpretation.

**The Effects of a Training and Nutrition Program on Body Composition of American Football Players in Preparation for the NFL Combine**

Chris Horn^a^, Paulina Czartoryski^b^, Lia Leone^b^, Cassandra Evans^b^, Jake Geyer^b^, Brandon Marshall^a^, Lia Jiannine^b^, Corey Peacock^b^, Jose Antonio^b^

^a^House of Athlete, Weston, FL, USA; ^b^Exercise and Sport Science, NSU Florida, Davie, FL, USA

Corresponding author: Jose.Antonio@nova.edu

**Background**: The purpose of this investigation was to determine the effects of a training and nutrition program on body composition of American football players preparing for the NFL combine.

**Methods**: In this single-arm investigation, 11 collegiate football players (mean ± SD: age 21.7 ± 0.8 yr; height 188.6 ± 4.9 cm; weight; 100.1 ± 7.8 kg) who were in preparation for the NFL combine were provided with a nutrition and training regimen lasting ~8-12 weeks. Six days of the week consisted of athletic training corrections in the morning, followed by field and position work later in the day. Ninety-minute resistance training sessions were incorporated five days per week. Wednesdays consisted of solely chiropractic yoga and massage to emphasize recovery. All meals were preplanned and prepared by an onsite chef. Supplements were consumed routinely by each athlete pre and post workout. Body composition was assessed via dual-energy absorptiometry (DXA) pre and post training program.

**Results**: In this small cohort of highly trained American football players, we found a significant increase in weight (p = 0.03) (Pre 98.9 ± 8.5 vs Post 100.4 ± 7.4 kg) and fat-free mass (p = 0.02) (Pre 79.1 ± 4.3 vs Post 80.5 ± 4.1 kg) pre and post. We did not find any differences in fat-free mass.

**Conclusions**: After completing an 8-12-week training and nutrition program, the primary change seen in trained American football players was an increase in fat-free mass. Based on the current data, favorable changes in body composition can be made with proper implementation of exercise and nutrition.

**High-dose Curcumin Supplementation Induces a Decrease in Hemoglobin and Fatigue in a Resistance-Trained Male: a Case Report**

Jose Antonio PhD^a^

^a^Exercise and Sport Science, NSU Florida, Davie, Florida, USA

Corresponding author: Jose.Antonio@nova.edu

**Background**: This is a case report of resistance-trained male that had been supplementing with fairly high-dose curcumin for a period of ~1 year.

**Methods**: The subject is a former powerlifter and had been engaged in a regular resistance training program for approximately 31 years (weight – 104.5 kilograms, height – 182.9 centimeters, age 47 years). The subject trains 5 days a week with heavy resistance exercise, with 10 minutes of aerobic exercise used as a light warm-up before resistance exercise. Once per week, he does a 15-mile bike ride at a low intensity. The subject has a family history of arthritis but has always used supplements to such as fish oil to reduce joint inflammation while refraining from using NSAIDS. The subject consumed the following supplements on a daily basis: pre-workout formula containing 5 grams of creatine monohydrate, 6 grams of citrulline malate, 7.5 grams of essential amino acids, 2.5 grams of betaine anhydrous, and 2.4 grams of beta alanine. In addition, he consumed 40 grams of whey protein isolate and 500 mg of fish oil daily. In early January 2019, the subject started using curcumin (5 grams daily).

**Results**: Table 1 provides an overview of results. The subject’s hemoglobin and hematocrit were normal before starting curcumin. The subject noticed considerable reduction in joint pain and inflammation after 1 month of taking the product. In June, six months of consistently using curcumin, the subject started experiencing extreme fatigue during high-intensity resistance exercise. The subject commented that after high repetition squats he would ‘have to lay down between sets’. The subject then noticed that during his weekend bike rides on Saturdays, he started needing to take breaks during the ride to catch his breath. In November, upon a physician’s advice and his own personal ‘research’ on curcumin, the subject ceased supplementation of curcumin.

**Table 1.** Hemoglobin values

	**October 15, 2020**	**November 16, 2020**	**December 18, 2020**
Hemoglobin	11.8 g/dL	13.1 g/dL	14.6 g/dL
Hematocrit	40.9 %	44.0 %	47.5%

The desired range for hemoglobin is 13.2-17.1 g/dL

One month after ceasing curcumin supplementation, the subject’s stamina started to return and he could perform aerobic and resistance exercises without extreme fatigue.

**Conclusions**: Chronic high-dose curcumin may induce anemia thus resulting in extreme fatigue during exercise.

**Daily Supplementation of 5-Hydroxytryptophan (5-HTP) in Exercise-Trained Men and Women**

Jason Curtis PhD^a^, Paulina Czartoryski^a^, Cassandra Evans^a^, Autumn Elwell^a^, Anna Santucci^a^, Lia Leone^a^, Jake Geyer^a^, Veronica Mekhail^a^, Valentina Vita^a^, Amanda Pultorak^a^, Anya Ellerbroek^a^, Jackie Kaminski^a^, Juan Carlos Santana^b^, Lia Jiannine PhD^a^, Corey Peacock^a^, Jose Antonio PhD^a^

^a^Exercise and Sport Science, NSU Florida, Davie Florida, USA; ^b^Institute of Human Performance, Boca Raton Florida, USA

Corresponding author: Jose.Antonio@nova.edu

**Background**: The purpose of this investigation was to determine the effects of supplementing with 100 mg daily of 5-Hydroxytryptophan (5-HTP) on indices of body composition in exercise-trained men and women.

**Methods**: Sixty-one subjects were screened and recruited to participate in this investigation. Subjects were randomized into a treatment (n = 31, 12 males; 100 mg 5-HTP daily; CLEANMOOD**®**) or a placebo (n = 17, 6 males; maltodextrin). Thirteen subjects either did not complete the study or their data was not used due to lack of compliance. Body composition was assessed pre- and post-treatment after eight weeks via a multi-frequency bioelectrical impedance device (InBody**®** 270). Subjects were instructed not to change their training habits; moreover, they were instructed to track their diet ~2-3 per week using a mobile app (MyFitnessPal).

**Results**: The characteristics of the treatment and placebo groups were as follows (age: 5-HTP 3?10, placebo 29?11; height cm: 5-HTP 171.0?8.7, placebo 167.7?15.0; body mass kg: 72.9?13.4, placebo 69.1?11.9). There were no changes in food intake (i.e. total energy intake or grams of macronutrients) between groups. Lean body mass, total body water, and % body fat did not change significantly. Fat mass decreased significantly pre versus post in the 5-HTP group (## p = 0.0186) but did not change in the placebo group (p = 0.5812). Moreover, the change in fat mass was significantly different between the 5-HTP and placebo group (#p = 0.048) (Table 1).

**Table 1.** Fat mass

	5-HTP	Placebo
**Fat Mass pre**	15.3?6.0	12.4?4.5
**Fat Mass post**	14.6?5.7 ##	12.6?4.2
**Fat mass change**	−0.7?1.7 #	0.2?1.0

**Conclusions**: Based on the limited data from this investigation, daily supplementation with 100 mg of 5-HTP might affect body composition.

**Acknowledgments**: We would like to thank NURA for providing the support for this investigation.

**Indices of Mood and Sleep in Trained Men and Women**

Lia Jiannine PhD^a^, Paulina Czartoryski^a^, Jason Curtis PhD^a^, Cassandra Evans^a^, Jackie Kaminski^a^, Autumn Elwell^a^, Anna Santucci^a^, Lia Leone^a^, Jake Geyer^a^, Veronica Mekhail^a^, Valentina Vita^a^, Amanda Pultorak^a^, Anya Ellerbroek^a^, Juan Carlos Santana^b^, Jaimie Tartar PhD^c^, Jose Antonio PhD^a^

^a^Exercise and Sport Science, NSU Florida, Davie, Florida, USA; ^b^Institute of Human Performance, Boca Raton, Florida, USA; ^c^Psychology and Neuroscience, NSU Florida, David Florida, USA

Corresponding author: Jose.Antonio@nova.edu

**Background**: The aim of this investigation was to identify any sex differences in mood and sleep of trained men and women.

**Methods**: Exercise-trained men (n = 24) and women (n = 31) (mean ± SD: age 30 ± 10 years; height 169.4 ± 12.1 centimeters; weight; 72.2 ± 13.9 kilograms; % body fat 20.4 ± 8.1%; mean hours per week of aerobic training 5 ± 2; mean hours per week of resistance training 5 ± 4, mean total years of training 14 ± 10 years) completed questionnaires assessing mood (Profile of Mood States) and sleep (Pittsburgh Sleep Quality).

**Results**: Tables 1 and 2 provide an overview of results. There were no differences in men and women’s Profile of Moods States scores and Global Pittsburgh Sleep Quality Index scores.

**Table 1.** Profile of mood states scores

	Male	Female
Total Mood Disturbance Score	10.9 ± 17.6	8.5 ± 21.0
Anger	5.5 ± 4.1	4.5 ± 5.7
Confusion	5.5 ± 3.8	5.3 ± 3.7
Depression	3.7 ± 4.1	3.8 ± 5.7
Fatigue	5.7 ± 5.6	5.3 ± 4.9
Tension	6.8 ± 4.7	6.3 ± 4.5
Vigor	16.4 ± 6.0	16.7 ± 5.8

**Table 2.** Pittsburgh sleep quality index scores

	Male	Female
Subjective Sleep Quality	1.0 ± 0.8	0.8 ± 0.7
Sleep Latency	1.5 ± 0.9	1.2 ± 1.0
Sleep Duration	1.4 ± 0.8	1.4 ± 1.1
Habitual Sleep Efficiency	2.5 ± 1.1	2.52 ± 1.4
Sleep Disturbances	1.1 ± 0.4	1.1 ± 0.6
Use of Sleep Medication	0.3 ± 0.8	0.7 ± 1.0
Daytime Dysfunction	1.3 ± 0.9	0.9 ± 0.7
Global PSQI	8.7 ± 3.3	8.0 ± 3.7

**Conclusions**: We did not observe any differences between men and women in measures of mood and sleep.

**Poor Sleep quality in Trained Men and Women**

Paulina Czartoryski^a^, Cassandra Evans^a^, Jackie Kaminski^a^, Autumn Elwell^a^, Anna Santucci^a^, Lia Leone^a^, Jake Geyer^a^, Veronica Mekhail^a^, Valentina Vita^a^, Amanda Pultorak^a^, Anya Ellerbroek^a^, Juan Carlos Santana^b^, Lia Jiannine PhD^a^, Jason Curtis^a^, Corey Peacock^a^, Jaime Tartar PhD^c^, Jose Antonio PhD^a^

^a^Exercise and Sport Science, NSU Florida, Davie, Florida, USA; ^b^Institute of Human Performance, Boca Raton, Florida, USA; ^c^Department of Neuroscience, NSU Florida, David, Florida, USA

Corresponding author: Jose.Antonio@nova.edu

**Background**: The purpose of this study was to assess sleep quality in trained men and women.

**Methods**: Fifty-five trained men (n = 24) and women (n = 31) (mean ± SD): age 30 ± 10 years, aerobic exercise per week 5 ± 4 hours, resistance exercise per week 5 ± 2 hours, total years of training 14 ± 10) volunteered to fill out a questionnaire to assess sleep quality (i.e. Pittsburg Sleep Quality Index). The Pittsburg Sleep quality Index (PSQI) is a self-reported questionnaire that assesses sleep quality (i.e. it differentiates ‘poor’ from ‘good’ sleep quality be measuring seven components: subjective sleep quality, sleep latency (i.e. how long it takes to fall asleep), sleep duration, habitual sleep efficiency (i.e. percentage of time in bed that one is asleep), sleep disturbances, use of sleeping medication, and daytime dysfunction.

**Results**: Of the 55 subjects, only 2 men and 5 women exhibited scores of less than 5 (indicative of good sleep quality). The Global PSQI scores were (mean ± SD): men (8.7 ± 3.3), women (8.0 ± 3.6). The category with the worst scores for both men at (2.50 ± 1.14) and women (2.52 ± 1.36) was ‘habitual sleep efficiency’.

**Conclusions**: The majority (87%) of the trained men and women in this study exhibit poor sleep quality. Although men would rate their ‘subjective sleep quality’ at ‘fairly good’ (1.04 ± 0.75) and women fell in the ‘very good’ range (0.81 ± 0.70), their overall PSQI scores would indicate neither sex obtains good sleep quality.

**Sex Differences in the Psychomotor Vigilance Test (PVT): Trained Men vs. Women**

Veronica Mekhail^a^, Cassandra Evans^a^, Jackie Kaminski^a^, Paulina Czartoryski^a^, Jason Curtis PhD^a^, Lia Jiannine PhD^a^, Corey Peacock PhD^a^, Jaime Tartar PhD^a^, Jose Antonio PhD^a^

^a^Department of Exercise and Sport Science, NSU Florida, Davie, FL, USA; ^b^Department of Neuroscience, NSU Florida, Davie, FL, USA

Corresponding author: Jose.Antonio@nova.edu

**Background**: The aim of this investigation was to measure sustained attention via the psychomotor vigilance test (PVT) in exercise-trained individuals.

**Methods**: Thirty-one exercise-trained men (n = 13) and women (n = 18) (mean ± SD: age men 29 ± 6 and women 29 ± 8 years; height men 1.8 ± 0.8 and women 1.6 ± 0.6 meters; body mass men 85 ± 10 and women 63 ± 6 kg; body fat % men 19.1 ± 10 and women 26.6 ± 7) volunteered for this study. Subjects performed a 5-minute PVT test. The PVT is a sustained-attention task that measures the speed in which subjects respond to a visual stimulus.

**Results**: Trained men have fewer false starts than trained women (Table 1). There was no difference in reaction time (PVT). All data are presented as the mean ± SD.

**Table 1.** Psychomotor Vigilance Test

	Male	Female	p value
Reaction time msec	273 ± 64	288 ± 32	0.4001
False starts	1.2 ± 1.6*	4.1 ± 4.0	0.0024

**Conclusions**: We did not observe any sex differences in reaction time; however, trained men had fewer false starts than trained women.

**The Effects of an Energy Drink on Measures of Cognition**

Cassandra Evans^a^, Jackie Kaminski^a^, Paulina Czartoryski^a^, Olivia Longmore^a^, Jake Geyer^a^, Amanda Pultorak^a^, Veronica Mekhail^a^, Lia Jiannine PhD^a^, Erik Bustillo, Anya Ellerbroek, Juan Carlos Santana^b^, Jaime Tartar PhD^c^, Corey Peacock^a^, Jose Antonio PhD^a^

^a^Exercise and Sport Science, NSU Florida, Davie, Florida, USA; ^b^Institute of Human Performance, Boca Raton, Florida USA; ^c^Psychology and Neuroscience, NSU Florida, David Florida USA

Corresponding author: Jose.Antonio@nova.edu

**Background**: The purpose of this study was to assess the effects of an energy drink (Redline® Cognitive Candy) on measures of cognition.

**Methods**: Twelve exercise-trained men (n = 3) and women (n = 9) (mean ± SD: age 25 ± 5 years; height 1.66 ± 0.08 meters; weight; 69.4 ± 12.3 kilograms; % body fat 21.4 ± 5.1%; daily caffeine intake 194 ± 144 milligrams; mean hours per week of aerobic training 5.1 ± 2.9; mean hours per week of resistance training 3.3 ± 1.5) volunteered for this randomized, double-blind, placebo-controlled, crossover trial. In a counterbalanced order, they consumed either the energy drink (product: Redline® Cognitive Candy, Weston Florida) or a similar tasting placebo drink. In the second visit after a washout period of at least three days, they consumed the alternate drink. Forty-five minutes post-consumption, they performed the following tests that are part of the NIH Toolbox **®** of cognition measures: Flanker Inhibitory Control and Attention Test, Dimensional Change Card Sort Test, and Pattern Comparison Processing Speed Test. In addition, each subject underwent the Psychomotor Vigilance Task. Lastly, they performed a treadmill run to exhaustion.

**Results**: There were no differences between conditions regarding any of the cognitive tasks except for Pattern Comparison Processing Speed (i.e. energy drink was better than the placebo). There were no differences in the psychomotor vigilance task (PVT) although the number of false starts was less in the PVT group. Furthermore, there were no differences in the treadmill time to exhaustion (Table 1).

**Table 1.** Cognitive and Physical Performance

	Energy Drink	Placebo Drink	*p value*
Flanker Inhibitory Control and Attention Test	112 ± 13	119 ± 19	0.1852
Dimensional Change Card Sort Test	121 ± 12	122 ± 17	0.9302
Pattern Comparison Processing Speed Test	134 ± 8*	127 ± 13	0.0301
Psychomotor Vigilance Test (msec)	288 ± 20	285 ± 25	0.6625
False Starts on the PVT Test	2.2 ± 1.6*	4.3 ± 2.4	0.0254
Time to Exhaustion (minutes)	7.09 ± 3.04	7.36 ± 3.07	0.5367

Data are expressed as the mean ± SD. Legend: msec – millisecond; PVT – psychomotor vigilance test. *Denotes differences that are statistically significant.

**Conclusions**: The acute consumption of an energy drink improves Pattern Comparison Processing Speed as well as lowering the number of false starts in a PVT task. However, there was no effect on running performance.

**Acknowledgments**: We would like to thank VPX (Weston FL) for their sponsorship of the ISSN and this investigation.

**The Effects of Pyrroloquinoline Quinone Supplementation with Endurance Training on Markers of Mitochondrial Activity and Protein Carbonyl Content in Untrained Males**

Paul S. Hwang^a^, Steven B. Machek^b^, Thomas D. Cardaci^c^, Dylan T. Wilburn^b^, Caelin Kim^b^, Emiliya S. Suezaki^b^ & Darryn S. Willoughby^d^, FISSN

^a^Department of Kinesiology, Vanguard University, Costa Mesa, CA, USA; ^b^Exercise & Biochemical Nutrition Laboratory, Department of Health, Human Performance, & Recreation, Robbins College of Health and Human Sciences, Baylor University, Waco, TX, USA; ^c^Department of Exercise Science, Arnold School of Public Health, University of South Carolina, Columbia, SC, USA; ^d^School of Exercise and Sport Science, Mayborn College of Health Sciences, University of Mary Hardin-Baylor, Belton, TX, USA

Corresponding author: dwilloughby@umhb.edu

**Background**: Pyrroloquinoline quinone (PQQ) is a novel supplement involved in physiological processes such as redox modulation and cellular energy metabolism. Similarly, endurance training elicits intramuscular adaptations on mitochondrial density and the expression of endogenous antioxidant enzymes. Prior published data observed that PQQ combined with endurance training significantly elevated skeletal muscle PGC-1α protein content, suggesting adaptations indicative of mitochondrial biogenesis in humans. However, no data exists on the combined effects of PQQ supplementation with endurance training on markers of electron transport chain (ETC) activity and oxidative stress in humans. Therefore, this investigation examined the effects of PQQ supplementation in young untrained males performing a 6-week aerobic exercise training program (ET) on markers of mitochondrial activity and protein carbonyl (PC) content.

**Methods**: In a randomized, double-blind, placebo-controlled design, untrained [<3 hr/wk exercise for ≥1 year prior to starting the study] males aged between 18-35 (n = 23) were randomly assigned to ingest 20 mg/day of encapsulated PQQ (n = 12) or cellulose placebo (PLC; n = 11) while participating in a supervised 6-week ET program (5 d/wk). Participants completed 2 research visits (PRE/POST) where markers of mitochondrial activity and oxidative stress were assessed. Participants underwent a percutaneous muscle biopsy from the *vastus lateralis* using the fine needle aspiration technique. Prepared homogenized muscle samples were used to assess citrate synthase (CS), ETC Complex I and IV activity and PC content. Factorial 2 × 2 Supplement [PQQ/PLC] by Time [PRE/POST] ANOVA with repeated measures was conducted for all criterion variables at a significance of p < 0.05.

**Results**: There was no significant supplement by time interactions for all variables (p > 0.05). However, there was a main time effect for Complex I and CS activity (*p* = <0.001). Pairwise comparisons revealed a greater increase in POST irrespective of group. Due to a significant baseline difference between groups for PC content, ANCOVA analysis was utilized to adjust for PRE-differences. No main effect for group was reported.

**Conclusions**: Based on the results, PQQ supplementation combined with endurance training may not additively impact markers of mitochondrial activity as assessed by Complex I, IV and CS activity over PLC in untrained males. Although PQQ did not elicit attenuating effects on PC content, lack of investigation on endogenous antioxidant targets or other biomarkers for oxidative stress warrants further research to ascertain any antioxidant role in humans. Future research should also explore PQQ supplemental effects on other endurance training modalities to identify any ergogenic potential on mitochondrial activity.

**The Effects of Betaine Supplementation on Fluid Balance**

Brandon D. Willingham^a,b^, Liliana I. Rentería^b^, Casey E. Greenwalt^b^, Haylee G. Colannino^b,^ Tristan J. Ragland^b^, Michael J. Ormsbee^b^

^a^Department of Kinesiology, Coastal Carolina University, Conway, South Carolina, 29,526, USA; ^b^Nutrition and Integrative Physiology, Institute of Sports Sciences & Medicine, Florida State University, Tallahassee, Florida, 32,304, USA

Corresponding author: mormsbee@fsu.edu

**Background**: Severe dehydration in humans may result in diminished cellular function, cardiac strain, and reduced cooling capacity, contributing to decreased athletic performance. One such nutritional strategy which may positively impact hydration status is the consumption of betaine. Betaine is an intracellular osmolyte and amino acid derivative found naturally in wheat products, spinach, and shrimp. However, these foods are typically consumed in an insufficient dose to observe improvements in hydration. Therefore, the purpose of this study was to examine the effects of betaine supplementation on fluid balance in recreationally active young men.

**Methods**: In a double-blind crossover design, betaine (50 mg·kg^−1^, 2x/day) or a rice flour placebo (50 mg·kg^−1^, 2x/day) was consumed for 7 days in young, recreationally active men (N = 11, 29.1 ± 5.2 y, 184.0 ± 7.8 cm, 78.5 ± 9.4 kg, 13.3 ± 6.9% body fat). Participants consumed 6 ml·kg^−1^ water with every supplemental dose but were otherwise allowed to drink ad libitum during the loading period. On days 0 and 7 of supplementation, participants arrived at the laboratory in the morning, following an overnight fast (7-9 hrs) having abstained from caffeine (previous 12 hrs), as well as alcohol and exercise (previous 24 hrs). Hydration status was measured via bioelectrical impedance spectroscopy and blood analysis. Markers of hydration and fluid volume included intracellular fluid (ICF), extracellular fluid (ECF), total body water (TBW), plasma osmolality, and plasma concentrations of the following: sodium (Na^+^), potassium (K^+^), chloride (Cl^−^), albumin (ALB), and total protein (TPRO). Paired samples t-tests were used within and between groups to compare differences in these markers on day 0 and 7 of supplementation.

**Results**: No significant differences were found for any marker of hydration between or within groups. Therefore, data below are presented comparing pre- vs. post-supplementation for the betaine group. Seven days of betaine supplementation did not result in significantly greater ICF volume (pre: 26.21 ± 3.22 L, post: 26.62 ± 3.19 L; p = 0.385), ECF volume (pre: 19.60 ± 2.14 L, post: 19.47 ± 1.99 L; p = 0.493), or TBW (pre: 45.81 ± 5.18 L, post: 46.09 ± 5.09 L; p = 0.626). Likewise, plasma osmolality (p = 0.426), Na^+^ (p = 0.367), K^+^ (p = 0.572), and Cl^−^ (p = 0.087) were not significantly different. Lastly, no significant differences were found for the indirect measures of oncotic pressure, ALB (pre: 4.19 ± 0.21 g·dL^−1^, post: 4.23 ± 0.17 g·dL^−1^; p = 0.544) or TPRO (pre: 7.26 ± 0.53 g·dL^−1^, post: 7.50 ± 0.39 g·dL^−1^; p = 0.086).

**Conclusions**: In the present study, seven days of supplemental betaine failed to improve physiological markers of hydration status in young, recreationally active men in free-living environments without a significant fluid stressor.

**Acknowledgments**: This study was supported by a grant from NOW Foods, Inc.

**Racial Differences in Iron Status throughout the Season in Women Collegiate Soccer Athletes**

Bridget A. McFadden^a^, Harry P. Cintineo^a^, Alexa J. Chandler^a^, Brittany N. Bozzini^c^, Michelle A. Arent^b^, Thomas D. Cardaci^a^, Alan J. Walker^d^, Shawn M. Arent^a^

^a^Department of Exercise Science, University of South Carolina, Columbia, SC, USA; ^b^Department of Health Promotion, Education, and Behavior, University of South Carolina, Columbia, SC, USA; ^c^U.S. Army Research Institute of Environmental Medicine, Natick, MA, USA; ^d^Department of Exercise Science, Lebanon Valley College, Annville, PA, USA

Corresponding author: bm39@mailbox.sc.edu

**Background**: Iron is an essential trace element involved in a multitude of metabolic processes including oxygen transport and utilization, and therefore may significantly affect exercise capacity. Declines in iron have been shown throughout a season in female collegiate soccer athletes. The purpose of this study was to compare hematological status across races in women collegiate soccer athletes.

**Methods**: National Collegiate Athletic Association Division I female athletes (N = 28; M_age_ = 19 ± 1y) were monitored over the course of a soccer season and retrospectively divided into two groups based on race (Black [n = 8] and White/non-Hispanic [n = 19]). Blood draws were performed prior to preseason (S1) as well as weeks 4, 8, & 12 of the season (S2-S4). Athletes were instructed to arrive between 0700-0900 h, 18-24 hours post-game in a fasted, euhydrated state. Serum iron (Fe), percent saturation (%SAT), iron-binding capacity (IBC), ferritin (Fer), transferrin (TfR), and hemoglobin (Hb) were analyzed as indicators of iron status. Mixed effects models were conducted to assess changes over time through the season and examine race-by-time interactions. For each biomarker, area under the curve (AUC) was calculated, and T-tests were conducted to compare differences by race. For all analyses, significance was set at p ≤ 0.05. Effect sizes were determined using Cohen’s *d*.

**Results**: Athletes experienced declines in Fe (p = 0.001), %SAT (p = 0.02), Fer (p = 0.05), and Hb (p < 0.001) from baseline (S1). No changes in IBC and TfR were seen from baseline (p > 0.05). No race-by-time interactions were observed across the season, however significant racial differences in hematological status were found. AUC analysis revealed Black players exhibited significantly lower Fe (p = 0.01, *d* = −1.25), %SAT (p = 0.01; *d* = −1.1), and Hb (p = 0.01, *d* = −2.39) compared to White players. No differences were seen with IBC (*d* = 0.28), TfR (*d* = 0.32), and Fer (*d* = −0.47) (p > 0.05) throughout the season.

**Conclusions**: Female athletes experience significant declines in iron status across a collegiate soccer season which may impact performance. Both Black and White players exhibited similar patterns of decline in markers of hematological status. However, despite similar decreases, Black athletes experienced lower iron levels compared to White athletes throughout the entire season. These values approached clinical criteria for iron deficiency by the end of the season (S4). Further studies are needed to elucidate the mechanisms behind the differences of iron status by race, such as iron metabolism, genetic predisposition, or nutritional disparities, to help inform recommendations and personalized nutrition interventions in order to optimize performance in female athletes.

**Acknowledgments**: Funding provided by Quest Diagnostics.

**7 Days of L-Citrulline Supplementation Does Not Improve Functional Performance in Older Active Women**

Jeremy Townsend^a,^ Shameka Freeman^a^, Megan Jones^a^, Jaclyn Morimune^a^, Laurel Littlefield^a^, Ruth Henry^a^

^a^Exercise and Nutrition Graduate Program, Lipscomb University, Nashville, TN 37204, USA

Corresponding author: jrtownsend@lipscomb.edu

**Background**: L-citrulline is a non-essential amino acid which increases circulating nitric oxide (NO) levels following consumption through the NO-synthase pathway. NO then exerts a variety of biological processes including vasodilation, improved muscle contractility, and a reduction in fatigue during exercise. While a number of investigations have examined the efficacy of L-citrulline supplementation in a young population, more data is needed pertaining to older adults. Thus, the aim of this study was to test the hypothesis that 7 days of L-citrulline supplementation will improve functional performance and indices of cardiovascular health and in older active women.

**Methods**: Sixteen physically active women (66.9 ± 5.6 yrs, 1.65 ± 0.5 m, 71.7 ± 16.7 kg,) volunteered to participate in this randomized, double-blind, crossover study. All participants exercised at least 3 times per week and participated in fitness class consisting of full-body resistance training. Following enrollment, participants reported to the laboratory for a familiarization session with all experimental testing procedures and body composition testing. All subsequent testing visits occurred at a local community fitness center and consisted testing before and after 7 days of supplementation with L-citrulline (CIT) or placebo (PL). Upon arrival for these visits, resting blood pressure and heart rate was obtained with the participant in a seated position. Following blood pressure assessment, participants completed the following tests in order: handgrip strength, timed get-up-and-go (GUG), 30 second Sit-to-stand (STS) for repetitions, and a 6-minute walking test for distance. Immediately following the 6-minute walk test, post-exercise blood pressure and heart rate was recorded. Between treatments, a 2-week washout period was provided before participants crossed-over to the other condition. Data were analyzed via separate repeated measures analysis of variance.

**Results**: Results indicate there were no significant time by group interactions for handgrip strength (p = 0.593), GUG (p = 0.877), STS (p = 0.860), or 6 min walk test (p = 0.563) between CIT and PL treatments. There was a significant time by treatment interaction observed for resting diastolic blood pressure (p = 0.028) with lower values observed following 7 days of CIT supplementation compared to PL. There were no differences (p > 0.05) observed between treatments for any other measure of pre- or post-exercise heart rate and blood pressure.

**Conclusions**: These data indicate that 7 days of L-citrulline supplementation did not influence measures of functional performance but may lower resting diastolic blood pressure in older active women.

**Self-Reported Nutritional Habits of High-Intensity Functional Training Participants**

Regis C. Pearson^a,^ Nathan T. Jenkins^a^

^a^Department of Kinesiology, University of Georgia, Athens, GA, USA

Corresponding author: regis.pearson@uga.edu

**Background**: High-intensity functional training (HIFT) is characterized by the combination of high-intensity aerobic conditioning, resistance training, and bodyweight exercises. Despite reports of more than 4 million participants worldwide, there has been little systematic evaluation of nutritional habits among people who participate in HIFT. The purpose of this study was to evaluate HIFT participants’ nutritional habits using a survey research approach.

**Methods**: A total of 450 respondents with greater than six months of HIFT participation completed a survey assessing their demographics and nutritional goals. Participants then completed the Dietary Health Questionnaire-III. 443 respondents were used for relative macronutrient analysis due to not reporting body weight (*n* = 7). Multiple linear regressions were performed to detect associations between dependent (e.g. dietary intake) and independent (e.g. sex, BMI, age, exercise frequency, and nutritional goals) variables. Binary logistic regressions were performed to examine odds ratios between dependent (e.g. nutritional goals) and independent (e.g. sex, BMI, age, and exercise frequency) variables.

**Results**: BMI, age, exercise frequency, and a nutritional goal of lose fat mass, weight gain and weight loss were significant predictors of relative energy intake (*P* ≤ 0.031). BMI, age, exercise frequency, and a nutritional goal of weight loss and weight gain were significant predictors of relative carbohydrate intake (*P* ≤ 0.018). Sex, BMI, age, and exercise frequency were significant predictors of relative protein intake (*P* ≤ 0.032). Sex, BMI, and age were significant predictors of relative fat intake (*P* ≤ 0.011). Age and exercise frequency were significant predictors of relative alcohol intake (*P* ≤ 0.002). Males were less likely to select lose fat mass (29.9%) and weight loss (18.6%), and more likely to select gain muscle mass (65.9%) and weight gain (94.5%) as a nutritional goal compared to females. Respondents with higher BMI were more likely to select fat loss (53.0%) and weight loss (56.2%), and less likely to select support performance (48.5%), weight maintenance (48.5%), muscle gain (48.2%), and weight gain (42.9%) as a nutritional goal. Older respondents were less likely to select gain muscle mass (49.5%) and weight gain (47.9%) as nutritional goals. Respondents participating in more exercise sessions wk^−1^ were 47.8% less likely to select weight maintenance as a nutritional goal.

**Conclusions**: Self-reported eating behaviors appear to vary among sex, weight status, age, and exercise frequency of HIFT participants. Nutritional goals of HIFT participants are linked to dietary intake, specifically relative energy, carbohydrate, and micronutrient intake.

**Acknowledgments**: RCP is supported by a UGA Graduate School Georgia Research Education Award Traineeship.

**COI**: NTJ reports consultancies with CrossFit, Inc. and Renaissance Periodization, LLC separate from the submitted work.

**Characterization of Early and Late Menopause Transition on Body Composition, Cardiovascular Disease Risk, and Metabolic Flexibility**

Hannah E. Cabre^a,b^, Lacey M. Gould^a^, Amanda N. Gordon^a^, Andrew T. Hoyle^a^, A. Smith-Ryan^a,b^, FISSN

^a^Department of Exercise Physiology, University of North Carolina, Chapel Hill, NC 27599, USA; ^b^Human Movement Science Curriculum, Department of Allied Health Science, University of North Carolina at Chapel Hill, Chapel Hill, NC, 27,599, USA

**Background**: Cardiovascular disease (CVD) risk dramatically increases with menopause. Understanding cardiometabolic health and body composition changes throughout the menopause transition (MT) is essential for mitigating disease risk. The purpose of this study was to assess the differences between early and late MT on body composition, CVD risk factors, and metabolic flexibility.

**Methods**: Twenty-four healthy perimenopausal women (≥38 yrs and experiencing irregular menstrual cycles; Mean ± Standard Deviation[SD]: Age: 50.0 ± 3.4 yrs; BMI: 26.5 ± 5.4 kg/m^2^) were stratified as early MT (n = 13), defined as having increased variability in menstrual cycle length, and late MT (n = 11) defined by experiencing amenorrhea for ≥60 days before the final menstrual period. Body composition was assessed from a four-compartment model to estimate fat mass (FM), percent body fat (%BF), and fat-free mass (FFM). CVD risk was characterized by visceral fat (VAT) and cardiometabolic blood markers (total cholesterol [TC], glucose, and triglycerides). Metabolic flexibility was characterized by respiratory exchange ratio (RER) measured via indirect calorimetry during a progressive exercise bout at three intensities: low (LOW;<30% heart rate reserve [HRR]), moderate (MOD;30-50%HRR), and high (HIGH;50-75%HRR). Independent sample t-tests were used to compare body composition and CVD risk factors between early MT and late MT. One-way ANOVAs stratified by group with Bonferroni post-hoc comparisons were conducted by intensity (LOW, MOD, HIGH).

**Results**: While not statistically significant, %BF (Mean Difference [MD]±Standard Error [SE]: −3.5 ± 3.2 kg; p = 0.297) and FM (MD ± SE: −3.7 ± 4.4 kg; p = 0.409) were lower in early MT compared to late MT. FFM was similar between early MT and late MT (MD ± SE: 0.6 ± 2.7 kg; p = 0.840). For CVD risk factors, similarities were observed between groups regarding VAT, glucose, and triglycerides (p > 0.05). A notable but non significant difference was identified for TC (MD ± SE: −10.0 ± 15.1 mg/dL; p = 0.517). RER was lower but not significantly different in early MT (Mean ± SD: 0.54 ± 0.38 a.u.) compared to late MT (0.61 ± 0.31 a.u.; p = 0.646). RER was similar for MOD and HIGH intensities between early MT and late MT (p > 0.05).

**Conclusions**: The MT appears to cause an increase in FM and TC, and a decrease in FFM, which may have implications for CVD risk. The decrease in fat utilization during LOW intensity exercise may suggest late MT as a timeframe where metabolic inflexibility begins. Exercise and nutrition interventions that target body composition and metabolic flexibility in early MT may be instrumental in delaying increases in FM and supporting optimal fat metabolism during exercise.

**Acknowledgments**: This study was supported by the UNC Center for Women’s Health Research.

**The Accuracy of Five Resting Metabolic Rate Predictions in Men and Women Collegiate Athletes**

Jennifer B. Fields^a,b^, Margaret T. Jones^b,c,d^, Meghan K. Magee^b,c^, Andrew R. Jagim^e^

^a^Exercise Science and Athletic Training, Springfield College, Springfield, MA, USA; ^b^Patriot Performance Laboratory, Frank Pettrone Center for Sports Performance, George Mason University, Fairfax, VA, USA; ^c^Kinesiology, George Mason University, Manassas, VA, USA; ^d^Sport, Recreation, and Tourism Management, George Mason University, Fairfax, VA, USA; ^e^Sports Medicine Department, Mayo Clinic Health System, La Crosse, WI, USA

Corresponding author: jfields2@springfieldcollege.edu

**Background**: It is estimated that resting metabolic rate (RMR) accounts for ~60-70% of total daily energy expenditure for athletes. Therefore, accurate prediction of RMR is valuable for practitioners when determining energy requirements. The purpose of this study was to examine the accuracy of five commonly used RMR prediction equations in collegiate men and women athletes.

**Methods**: National Collegiate Athletic Association (NCAA) Division III men (n = 98; age: 20.1 ± 1.6 yrs; height: 181.6 ± 6.24 cm; weight: 92.66 ± 17.54 kg; fat-free mass (FFM): 77.15 ± 9.38 kg; fat percentage (BF%): 15.65 ± 8.80%) and women (n = 92; age: 19.45 ± 1.14 yrs; height: 168.04 ± 6.58 cm; weight: 65.16 ± 11.05 kg; FFM: 49.61 ± 6.36 kg; BF%: 22.71 ± 5.98%) participated. RMR was measured using indirect calorimetry and body composition was analyzed using air displacement plethysmography. RMR was estimated using five prediction equations (Table 1). A one-way analysis of variance with Bonferroni post hoc analyses was selected to determine mean differences between measured and predicted RMR. Linear regression analysis was used to assess the accuracy of each RMR prediction method (p < 0.05).

**Table 1.** Resting metabolic rate prediction equations

**Harris-Benedict** Men RMR: 66.47 + 13.75 x body mass (kg) + 5 x height (cm) – 6.76 x age (yrs) Women RMR: 655.1 + 9.56 x body mass (kg) + 1.85 x height (cm) – 4.68 x age (yrs)
**Mifflin St. Jeor** RMR: 9.99 x body mass (kg) + 6.25 x height (cm) – 4.92 x age (yrs) + 166 x sex (men 1; women 0) – 161
**Cunningham** RMR: 500 + 22 x FFM (kg)
**Nelson** RMR: FFM (kg) + 4.04 x fat mass (kg)
**De Lorenzo** RMR: −857 + 9 x body mass (kg) + 11.7 x height (cm).

**Results**: Mean RMR for men and women was 2595.33 ± 433.3 and 1708.1 ± 307.7 kcal, respectively. All five prediction equations significantly underestimated RMR in men (mean difference ± SD): Cunningham (387.5 ± 331.2 kcal, p < 0.001), Nelson (529.6 ± 299.7 kcal, p < 0.001), Mifflin St. Jeor (620.3 ± 305.2 kcal, p < 0.001), Harris Benedict (471 ± 309.0 kcal, p < 0.001), and De Lorenzo (485.5 ± 309.0 kcal, p < 0.001). In women, the Cunningham (116.0 ± 212.62 kcal, p < 0.001), Nelson (369 ± 189.6 kcal, p < 0.001), Mifflin St. Jeor (267.4 ± 201.5 kcal, p < 0.001), and Harris Benedict (215.3 ± 214.7 kcal, p < 0.001) equations underestimated RMR. There was no difference between the De Lorenzo equation (14.1 ± 201.2) and measured RMR (p = 0.55) for women. Linear regression showed the Harris Benedict equation was the best predictor of measured RMR in men (r^2^ = 0.616, Beta = 0.785, t = 12.03, p < 0.001) and women (r^2^ = 0.705, Beta = 0.840, t = 12.27, p < 0.001).

**Conclusions**: All RMR prediction equations underestimated RMR in men collegiate athletes. In addition, De Lorenzo was the only equation that did not underestimate RMR in women collegiate athletes. The Harris-Benedict equation was the most accurate predictor for men and women athletes; therefore, this study provides early evidence that Harris-Benedict might be the best equation when predicting RMR in Division III athletes.

**UC-II® Collagen Improved Knee Range of Motion (ROM) in Healthy Subjects: A Randomized, Double-Blind, Placebo-Controlled Clinical Study**

Vijaya Juturu^a^, Katharina Knaub^b^, Wilfried Alt^c^, Christiane Schön^b^, Shane Durkee^a^ and Zainulabedin Saiyed^a^

^a^Research & Development, Lonza CHI, Morristown, NJ 08902, USA; ^b^BioTeSys GmbH, Schelztorstraße 54-56, 73728 Esslingen, Baden-Württemberg, Germany; ^c^Institute of Sports Science and Kinesiology, University of Stuttgart, Allmandring 28, 70569 Stuttgart, Baden-Württemberg, Germany

Corresponding author: vijaya.juturu@lonza.com

**Background**: Joint-related stress models have already been used in the past to induce standardized load on physical structures, thus leading to changes on perceived stress on joint as accurate as possible in healthy individuals. Previous preclinical models and clinical studies support the safety and efficacy of UC-II® collagen in modulating joint discomfort in osteoarthritis and in healthy subjects. The purpose of this study was to assess the effect of UC-II® collagen on knee joint function and mobility as measured by range of motion (ROM) in healthy subjects who experience activity related joint discomfort.

**Methods**: This randomized, double-blind, placebo-controlled study was conducted in healthy subjects who had no prior history of osteoarthritis, inflammatory or metabolic joint diseases. Ninety six (n = 96, 20 – 55 years old) subjects who reported joint discomfort of at least 5 on an 11-point Likert scale while performing at least 30 steps during the Single leg step down test (SLSD) were randomized to receive either placebo (PLA, n = 48) or the 40 mg UC-II® collagen (providing ≥3% undenatured type II collagen (Collagen), n = 48) supplementation daily for 6 months. Joint flexibility was measured from the knee ROM flexion and extension using a goniometer.

**Results**: At the end of the study, a statistically significant increase in knee ROM flexion was observed in the collagen group versus the PLA group (3.23 degrees vs. 0.21 degrees, respectively; p = 0.025). Also, an increase of knee ROM extension by 2.21 degrees was observed in the collagen group and a slight increase by 1.27 degrees in the PLA group. Analysis of changes over time within each intervention group showed a significant increase only in the collagen group (p = 0.0061), whereas the change in PLA group was not significant (p = 0.2540). Subgroup analysis by age showed a significant increase of ROM flexion in subjects of age > 35 years after collagen intervention in comparisons to PLA (6.79 degrees in Collagen vs. 0.30 degrees in PLA; p = 0.0092). Overall, these results suggest undenatured type II collagen improves joint flexibility, extensibility and mobility.

**Conclusion**: Daily supplementation with 40 mg of UC-II® collagen improved the knee joint function in healthy subjects who experience activity-related joint discomfort.

**Trial registration**: German Clinical Trial Register DRKS00018792

**Acknowledgments/Sponsor: Lonza CHI Inc.**

**Fish Oil Supplementation Combined with a Resistance Training Program Enhances Lower-Body strength in Young Women.**

Jeffery L. Heileson^a^, Hanna M. Merrell^b^, Steven B. Machek^a^, Dylan T. Wilburn^a^, Jeffrey S. Forsse^b^, LesLee K. Funderburk^a,c^

^a^Department of Health, Human Performance, and Recreation, Baylor University, Waco, TX, 76798, USA; ^b^Baylor Laboratories for Exercise Science and Technology, Department of Health, Human Performance, and Recreation, Baylor University, Waco, TX, 76798, USA; ^c^Family and Consumer Sciences, Baylor University, Waco, TX, 76798, USA

Corresponding author: leslee_funderburk@baylor.edu

**Background**: The purpose of this study was to determine if fish oil (FO) supplementation enhances training adaptations, namely hypertrophy and strength, following a structured 10-week resistance exercise (RE) program.

**Methods**: Eight young, apparently healthy and recreationally trained women (age 28.4 [5.5], height 167.3 [8.8] cm, weight 69.6 [11.1] kg, body fat percentage 31.4% [3.8]) were enrolled and completed the study. All participants conducted RE program comprised three full-body sessions RE on nonconsecutive days for 10-weeks that consisted of the bench press, military press, wide grip lateral pulldown, seated cable row, back squat, leg press, and leg extension/curl. Participants conducted 4 sets of 10 repetitions (reps) for bench press and back squat and 3 sets of 10 reps for the remaining exercises. One session per week was under the supervision of a member of the research staff. The participants were divided into two groups: 1) ~4 g/d FO (EPA+DHA) or 2) ~4 g/d safflower oil (placebo [PL]). Body composition (body mass, lean body mass [LBM], and body fat percentage [%BF]) via dual-energy x-ray absorptiometry and upper-body and lower-body strength via 1RM bench press and 1RM back squat, respectively, was assessed at baseline and after 10-weeks. Repeated measures within-between ANOVA was used to determine group by time interactions. All between-group differences were analyzed using effect sizes (Cohen’s *d*). The effect magnitudes were classified as follows: *d* < 0.20, trivial; *d* = 0.20–0.49, small; *d* = 0.50–0.79, moderate; *d* ≥ 0.80, large. Statistical significance was set at *p* < 0.05.

**Results**: Lower-body strength (*p* < .001) and upper-body strength (*p* < .001) and LBM (*p* = .005) significantly increased over time and %BF (*p* = .007) significantly decreased over time in both groups. FO supplementation significantly increased lower-body strength (*p* = .024, *d* = 2.17) compared to PL. FO supplementation increased upper-body strength (*p* = .056, *d* = 1.78), LBM (*p* = .272, *d* = 0.99), and decreased %BF (*p* = .150, *d* = 1.35) similarly to PL.

**Conclusions**: In recreationally trained women, FO supplementation enhances lower-body strength in conjunction with a 10-week RE program. It appears that FO supplementation may augment training adaptations from RE as upper-body strength and LBM also improved, while %BF decreased. While these values did not significantly differ from PL, it is clear from the between-group effect sizes and notable gross differences that the effects may be clinically relevant.

**Acknowledgments**: None

**Optimizing B-mode ultrasonography research methodology: Does image depth influence echo intensity?**

Ryan M. Girts^a^, Kylie K. Harmon^a^, Matt S. Stock^a^

^a^Neuromuscular Plasticity Laboratory, School of Kinesiology and Physical Therapy, University of Central Florida, Orlando, FL, USA

Corresponding author: Ryan.Girts@ucf.edu

**Background**: B-mode ultrasonography is an attractive option for assessing skeletal muscle composition in a variety of populations. Skeletal muscle echo intensity is often referred to as an indicator of ‘muscle quality’ and has been shown to be correlated with several aspects of lower-body performance. One methodological issue that has yet to be carefully examined is whether image depth influences the interpretation of echo intensity values. The purpose of this study was to investigate whether ultrasound echo intensity values of the vastus lateralis muscle are affected by the depth at which images are captured.

**Methods**: Thirty-six healthy men and women participated in this study. Each participant rested in the supine position on a treatment table for 10 minutes prior to image acquisition. Ultrasound images of the vastus lateralis were captured in the sagittal plane. Images were taken at depths of 3, 3.5, 4, 4.5, 5,6, and 7 cm in random order. ImageJ software was used to quantify echo intensity (arbitrary units [A.U.]). Mean differences in echo intensity at each depth were evaluated with a one-way repeated measures analysis of variance (ANOVA).

**Results**: A significant main effect for depth was observed (*F* = 102.494, *p* < 0.001, *η_p_^2^ *= 0.745). Pairwise comparisons indicated that when depth = 3 cm echo intensity values were significantly lower compared to all other settings (*p*’s < 0.001, *d*’s ≥ 1.111). Additionally, echo intensity values were significantly greater than all other settings when depth = 7 cm (*p*’s < 0.001, *d*’s ≥ 1.683).

**Conclusions**: Depth settings can have an impact on echo intensity values of the vastus lateralis muscle. Echo intensity values appeared stable between depths of 4 and 6 cm but deviated when depth settings were unable to capture the entire muscle or included excess extraneous tissue.

**Changes in Bone and Collagen Turnover Markers, CTX and P1NP, in Response to Collagen Supplementation in Active Adults.**

Shiloah A. Kviatkovsky, Robert C. Hickner, Liliana I. Rentería, Hannah E. Cabre, Stephanie D. Gipson, Brett R. Hanna, Haylee G. Colannino, Kathryn E. O’Connor, Anna S. Hayward, Michael J. Ormsbee

^a^Department of Nutrition & Integrative Physiology; ^b^Institute of Sports Sciences and Medicine

Corresponding author: mormsbee@fsu.edu

**Background**: Collagen peptides (CP) are thought to exert positive effects on bone and connective tissue. Serum C-terminal telopeptide (CTX) and procollagen type-I N-terminal propeptide (P1NP) are measures of bone and collagen turnover and have been strongly correlated with changes in bone mineral density. Directionality is dependent on intervention and mechanisms of action, and conflicting changes have been observed in CP interventions. Additionally, no data exist in males. The purpose of this double-blind randomized placebo-controlled trial was to characterize the effects of daily consumption of CP (SOLUGEL®) over 6-months on markers of CTX and P1NP in middle-aged active men and women.

**Methods**: Participants (N = 61) were randomized into three groups: 20 g CP (n = 21; male = 11), 10 g CP (n = 19; male = 7) of CP, or placebo (n = 21; male = 12). Serum was collected at baseline, 3-months, and 6-months to assess changes over time in CTX and P1NP. Repeated measures (RM) ANOVAs were used to assess interactions between experimental groups, and CTX or P1NP at each time point. Simple RM ANOVAs were used for post hoc analyses of significant interactions, and changes in scores are reported as percent change from baseline to 6-months for 20 g, 10 g, and placebo, respectively.

**Results**: A RM ANOVA approached a significant interaction for CTX and collagen group (*p* = 0.84; −7.8%, +13.6%, and +2.2%, respectively). There was a significant interaction for CTX and collagen group in males (*p* < 0.05; −5.8%, +48%, +13.2%, respectively), but not in females. Post hoc analysis in males revealed a significant difference (p < 0.05) between baseline and 6 month CTX in the 10 g, but not 20 g or placebo groups. There was a significant interaction between P1NP and collagen group (*p* < 0.05; −2.1%, −12.7%, +7.6%, respectively). Post hoc analyses revealed a significant difference (p < 0.05) in P1NP values between baseline and 6-months in 10 g, and between baseline and 3-months in placebo, but no significant differences in the 20 g group across time points. There were no significant differences in P1NP when analyzed by sex.

**Conclusions**: Six months of daily collagen peptide supplementation of 10 g, but not 20 g or placebo, yielded significant increases in CTX in males but not females. Conversely, P1NP significantly decreased with 10 g of collagen peptides, but not 20 g or placebo. No sex differences were observed for P1NP. These findings suggest 10 g to be effective and superior to 20 g of collagen peptides or placebo in changing biomarkers of bone and collagen turnover after 6-months of supplementation in active middle-aged men and women.

**Acknowledgments**: PB Leiner, part of Tessenderlo Group, funded this study.

The ISSM graduate and undergraduate student interns.

**Collagen Peptide Supplementation Improves Measures of Activities of Daily Living and Pain in Active Adults**

Shiloah A. Kviatkovsky, Robert C. Hickner, Stephanie D. Gipson, Hannah E. Cabre, Brett R. Hanna, Haylee G. Colannino, Kathryn E. O’Connor, Anna S. Hayward, Michael J. Ormsbee

^a^Department of Nutrition and Integrative Physiology; ^b^Institute of Sports Sciences and Medicine

Corresponding author: mormsbee@fsu.edu

**Background**: Pain is a major limiter of physical activity (PA) and activities of daily living (ADLs) in aging populations, contributing to lower quality of life and increased disease risks. Pharmacological interventions for managing pain often have negative side effects. Collagen peptides (CP) have been shown to be efficacious for growth and repair of connective tissue and to mitigate pain, but there are no long-term studies in healthy middle-aged active populations. Therefore, the purpose of this double-blind, randomized control trial was to determine the effects of daily consumption of CP (SOLUGEL®) over 6 months on markers of pain and ADLs in middle-aged lifelong exercisers.

**Methods**: Participants (N = 61) were randomized into three groups: 20 g (n = 21; male = 11), 10 g (n = 19; male = 7), or placebo (n = 21; male = 12). The Knee Injury and Osteoarthritis Outcome Score (KOOS) was used to assess changes over time (baseline, 3 months, and 6 months) in Pain and ADL scores. A 50% frequency split for physical activity during the study was used to dichotomize participants into low (LF) or high frequency (HF) exerciser groups. Repeated measures (RM) ANOVAs were used to assess interactions between experimental groups, exercise frequency groups, and scores (Pain or ADLs) at each time point. Simple repeated measures ANOVAs were used for post hoc analyses of significant interactions, and changes in scores are reported as percent change from baseline to 6 months. Increasing values reflect improvements in Pain and ADLs.

**Results**: There was a significant interaction (*p* < 0.05) between CP dose, exercise, and pain. Post hoc analysis indicated a significant interaction in HF exercisers between CP dose and pain (*p* < 0.05), but not in LF. Pain scores improved in the HF exercisers in both CP groups (20 g and 10 g), whereas pain scores worsened in placebo (+2.6%, +4.2%, −6.8%, respectively). There was a significant interaction between CP dose and ADLs (*p* < 0.05), with 20 g and 10 g improving in score, but not placebo, regardless of exercise frequency (+0.3%, +4.1%, −1.5%, respectively).

**Conclusions**: Daily CP intake of 20 g and 10 g over the course of 6 months yielded significant improvements in pain scores exclusively in HF exercisers, whereas no significant changes were observed in LF exercisers across groups. ADL scores improved with 10 g and remained stable with 20 g, whereas the placebo scores worsened, independent of exercise frequency. These findings indicate CP supplementation over 6 months may have protective as well as beneficial effects on ADLs and improvements in pain for middle-aged HF exercisers.

**Acknowledgments**: PB Leiner, part of Tessenderlo Group, funded this study.

The ISSM graduate and undergraduate student interns.

**Dose-Ranging Study of Acute ATP Supplementation to Improve Athletic Performance.**

Ralf Jäger, FISSN^a^, Fabrício E. Rossi^b^, Martin Purpura^a^, John A. Rathmacher^c,d^, John C. Fuller Jr^e^

^a^Increnovo LLC, Milwaukee, WI, USA; ^b^Immunometabolism of Skeletal Muscle and Exercise Research Group, Department of Physical Education, Federal University of Piauí (UFPI), Teresina-PI, Brazil; ^c^MTI BioTech, Inc., 2711 S. Loop Dr., Suite 4400, Ames, IA 50010, USA.; ^d^Dept. of Animal Science, Iowa State University, Ames, IA 50011, USA.; ^e^TSI USA LLC, 135 W Main St, Suite B, Missoula, MT 59802, USA.

Corresponding author: ralf.jaeger@increnovo.com

**Background**: Chronic oral ATP supplementation benefits cardiovascular health, muscular performance, body composition, and recovery while attenuating muscle breakdown and fatigue [1]. A single 400 mg dose of oral ATP supplementation improved lower body resistance training performance and energy expenditure in recreational resistance trained males [2], however, the minimal effective dose is currently unknown.

**Methods**: Twenty recreationally trained men (age 28.6 ± 1.0 years, weight 81.2 ± 2.0 kg, height 175.2 ± 1.4 cm, 1RM 141.5 ± 5.0 kg) consumed a single dose of either 400 mg, 200 mg, or 100 mg ATP (PEAK ATP, TSI USA LLC, Missoula, MT, USA) or a placebo in a randomized, placebo-controlled crossover design, separated by a one week wash out between treatments. After warm-up, each participant completed four sets until movement failure at 80% of 1RM (1-s eccentric and 1-s concentric) and 2 minutes of rest between sets.

**Results**: In comparison to placebo, 400 mg ATP significantly increased the number of set 1 repetitions (+13%, p < 0.04), and numerical increases in total repetitions (+7%) and total weight lifted (+6%). 200 mg ATP numerically increased set 1 repetitions (+6%), while 100 mg ATP showed no improvements over placebo. Interestingly, perceived exertion was lowest in the 100 and 400 mg groups and greatest in the 200 mg group (p < 0.02).

**Conclusions**

In this study, the effective minimal dose for acute oral ATP supplementation during resistance exercise was determined to be 400 mg.

**References**: [1] R. Jäger, M. Purpura, J.A. Rathmacher, J.C. Fuller Jr., L.M. Pitchford, F.E. Rossi, C.M. Kerksick. Health and ergogenic potential of oral adenosine-5′-triphosphate (ATP) supplementation. *J Funct Foods* **2021**, 78:104,357.

[2] M.C. Freitas, J.M. Cholewa, J. Gerosa-Neto, D.C. Goncalves, E.C. Caperuto, F.S. Lira, F.E. Rossi. A Single Dose of Oral ATP Supplementation Improves Performance and Physiological Response During Lower Body Resistance Exercise in Recreational Resistance-Trained Males. *J Strength Cond Res* **2019**, 33(12), 3345-3352.

**Acknowledgments**: The study was sponsored by TSI USA LLC. JCF is employed by TSI, and RJ and MP are paid consultants to TSI. JAR is employed by MTI BioTech, Inc.

**The Effect of 2-weeks of Intense Caloric Restriction (Rapid Fat Loss) on Body Composition in Resistance-Trained Males and Females**

Brooks A, Quint J, Ibrahim A, Mastrofini G, Smith T, Korte S, Dubie C, Fay N, Gegenheimer J, Waddell B, Callahan K, SanFilippo G, Siedler MR, Humphries MN, Lamadrid P, Ford S, Mathas D, Campbell BI.

Performance and Physique Enhancement Laboratory, Exercise Science Program, University of South Florida, Tampa, FL, USA

Corresponding author: bcampbell@usf.edu

**Background**: Aggressive caloric deficits have the advantage of inducing rapid fat loss. Unfortunately, such diets also induce negative consequences, such as the loss of fat-free mass (FFM). Further, some evidence suggests that the loss of FFM is associated with the severity and duration of the caloric deficit. Strategies employed to prevent the loss of FFM during a caloric deficit include resistance exercise and a high-protein diet. The purpose of this study was to examine the effects of a short-term, aggressive caloric deficit with high protein intake on body composition in resistance-trained males and females.

**Methods**: 32 resistance-trained males (n = 9) and females (n = 23) participated in this study (age: 25.5 ± 7.7 years; height: 168 ± 9.2 cm; body mass: 68.8 ± 12.8 kg). Following a period of baseline caloric tracking (maintenance phase), subjects were prescribed a 2-week 37.5% caloric deficit (diet phase). Subjects were instructed to ingest 2.2 g protein/kg body weight and continue their habitual training routine while dieting. After the diet phase, a 2-week *ad libitum* phase was followed. Body composition (body weight [BW], body fat percentage [BF%], fat mass [FM], fat-free mass [FFM], total body water [TBW] and dry FFM [dFFM: FFM-TBW]) was assessed at baseline, after the 2-week diet phase, and after the 2-week *ad libitum* phase. Data were analyzed using a repeated-measures ANOVA.

**Results**: Significant decreases were observed during the diet phase for BW, BF%, FM, FFM, and TBW (Table 1). Relative to baseline, the decreases were maintained during the *ad libitum* phase for BM, BF%, and FM. When the FFM compartment was adjusted for the changes in TBW (dry FFM), no decreases were observed for this variable.

**Table 1.** Body composition changes over time. Data reported as mean ± standard deviation (Bonferroni adjusted pairwise comparisons) and change scores relative to baseline

	Baseline	Diet Phase	Ad Libitum Phase
Body Mass (kg)	68.8 ± 12.8	67.3 ± 12.3 (p < 0.001) -p1.5	68.0 ± 12.4 (p = 0.004) -p0.8
Body Fat %	21.1 ± 7.9	19.9 ± 7.9 (p < 0.001) -p1.2	19.8 ± 7.7 (p < 0.001) -p1.3
Fat Mass (kg)	14.3 ± 5.9	13.3 ± 5.9 (p < 0.001) -p1.0	13.3 ± 5.8 (p < 0.001) -p1.0
Fat-Free Mass (kg)	54.5 ± 12.8	54.0 ± 12.3 (p = 0.015) -p0.5	54.7 ± 12.6 (p = 1.00) +p0.2
Dry Fat-Free Mass (kg)	15.5 ± 4.0	15.4 ± 3.8 (p = 1.000) -p0.1	15.6 ± 3.9 (p = 0.662) +p0.1
Total Body Water (kg)	39.0 ± 9.1	38.6 ± 8.8 (p = 0.002) -p0.4	39.1 ± 8.9 (p = 1.000) +p0.1

**Conclusions**: A prescribed 2-week 37.5% caloric deficit resulted in significant decreases in body fat. FFM was also significantly decreased during the caloric deficit but returned to baseline levels within 2 weeks of *ad-libitum* eating. When changes in total body water were accounted for, the dry FFM compartment was maintained throughout the diet and *ad libitum* phases.

**The Effect of 2-weeks of Intense Caloric Restriction (Rapid Fat Loss) on Resting Metabolic Rate and Eating Behavior in Resistance-Trained Males and Females**

Quint J, Brooks A, Ibrahim A, Mastrofini G, Smith T, Gonzales A, Tarr E, Rogers J, Klahr D, Clarke S, Reyes J, Pane O, Miller H, Schoucair D, Urrita M, Espinal M, Campbell BI.

Performance and Physique Enhancement Laboratory, Exercise Science Program, University of South Florida, Tampa, FL, USA

Corresponding author: bcampbell@usf.edu

**Background**: One of the negative consequences of prolonged caloric restriction is a suppression of resting metabolic rate (RMR), making future fat loss more difficult. Further, some evidence suggests that the suppression of RMR is associated with the severity of the caloric deficit. Strategies employed to prevent the suppression of RMR during a caloric deficit include resistance exercise and a high protein diet. The purpose of this study was to examine the effects of an intensive diet on RMR and eating behaviors in resistance-trained males and females following a high protein diet.

**Methods**: 32 resistance-trained males (n = 9) and females (n = 23) participated in this study (25.5 ± 7.7 years; 168 ± 9.2 cm; 68.8 ± 12.8 kgs). Following a maintenance phase, subjects were prescribed a 2-week 37.5% caloric deficit (diet phase) that included high protein (2.2 g/kg) and a continuation of their habitual training routine. A 2-week *ad libitum* phase followed the diet phase. RMR and eating behavior (measured via a 51-item three-factor eating questionnaire which measures an individual’s level of hunger, disinhibition [the loss of control in food intake], and dietary restraint [degree of cognitive control] in daily food intake) were assessed at baseline, after the 2-week diet phase, and after the 2-week *ad libitum* phase. Data were analyzed using a repeated measures ANOVA.

**Results**: A significant decrease over time for RMR was observed during the 2-week caloric deficit, but was recovered after the *ad libitum* phase. Specifically, RMR decreased from 1,627 ± 299 to 1,536 ± 271 kcals (−5.5%; p < 0.001)) during the diet phase and was partially recovered to 1,584 ± 267 kcals (−2.6% relative to baseline; p = 0.09) after the *ad libitum* phase. There were no changes over time in hunger (p = 0.949) or dietary restraint (p = 0.264) during the intervention. There was a significant change over time for disinhibition (p < 0.001), with scores decreasing from 6.8 ± 3.7 to 5.6 ± 3.1 during the diet phase (p = 0.004) and remaining lowered after the ad libitum phase (5.7 ± 3.1; p = 0.004 relative to baseline scores).

**Conclusions**: In resistance-trained males and females, a two-week prescribed caloric deficit of ~37.5% resulted in a significant decline in resting metabolic rate. High protein intake and resistance training were not able to mitigate this decline. As such, to maintain resting metabolic rate during caloric restriction, a short-term intense caloric deficit is not recommended. Eating behaviors were mostly unaffected during the intervention, with the exception of decreased disinhibition scores during the diet phase and *ad libitum* phase.

**Selective Androgen Receptor Modulator and Growth Hormone Secretagogue Use Impacts Body Composition, Health-Related Blood Markers, And Muscle Androgenic Hormone and Receptor Content: A Case Study**

Thomas D. Cardaci^a,b^, Steven B. Machek^b^, Dylan T. Wilburn^b^, Darryn S. Willoughby^c^

^a^Department of Exercise Science, University of South Carolina, Columbia, SC, USA; ^b^Department of Health, Human Performance, and Recreation, Baylor University, Waco, TX, USA; ^c^School of Exercise and Sport Science, University of Mary Harden-Baylor, Belton, TX, USA

**Background**: Selective androgen receptor modulators and growth hormone secretagogues ostensibly exert potent anabolic effects without negative androgenic steroid-associated side-effects. However, limited data exist describing their effects on health- and androgen-related biomarkers. The purpose of this case study was to report body composition changes in addition to androgen- and health-related biomarkers of an individual using Ligandrol/LGD-4033 and Ibutamoren/MK-677 and cross-sectionally examine intramuscular androgenic hormone and receptor concentrations.

**Methods**: A 25-year-old resistance-trained male ingested Ligandrol/LGD-4033 (10 mg) and Ibutamoren/MK-677 (15 mg) daily for 5 weeks. Blood samples and body composition metrics were obtained pre-, post-, and 4-wk post-cycle. Lean body mass (LBM), fat mass (FM), and bone mineral density (BMD) were assessed via DEXA. A comprehensive blood analysis was performed by a CLIA-certified lab. *Vastus lateralis* muscle biopsies were obtained post-cycle to assess intramuscular testosterone, dihydrotestosterone, and androgen receptor (AR) content via ELISA.

**Results**: There were increases pre- to post-cycle in BM (Δ + 6.0%), LBM (Δ + 5.5%), FM (Δ + 10.5%), and TBW (Δ + 2.5%). A decrease from post- to 4-wk post-cycle in BM (Δ-5.7%), LBM (Δ-4.5%), and TBW (Δ-2.1%) was observed, while FM remained elevated. BMD decreased from pre- to post-cycle (Δ-2.1%) and returned to baseline 4-wk post-cycle. Increases were observed from pre- to post-cycle in total cholesterol (Δ + 14.8%), triglycerides (Δ + 39.2%), and low-density lipoprotein (LDL;Δ + 40.0%) and remained elevated 4-wk post-cycle. High-density lipoprotein (HDL) decreased from pre- to post-cycle (Δ-36.4%) and returned to near-baseline values 4-wk post-cycle. Increases from pre- to post-cycle in aspartate aminotransferase (Δ + 95.8%) and alanine aminotransferase (Δ + 205.0%) were found and returned to near-baseline values 4-wk post-cycle. Decreases in total testosterone (Δ-62.3%), free testosterone (Δ-85.7%), and sex hormone-binding globulin (Δ-79.6%) from pre- to-post cycle were observed but returned to near-baseline values 4-wk post-cycle. Follicle stimulating hormone (FSH) was below clinical reference values post- (1.2IU/L) and 4-wk post-cycle (1.3IU/L), while luteinizing hormone was within its clinical reference values. Compared to non-users, intramuscular AR content was lower (−44.6%), while intramuscular testosterone and dihydrotestosterone were greater (+47.8%;+34.4%).

**Conclusions**: These data demonstrate Ligandrol/LGD-4033 and Ibutamoren/MK-677 substantially increased BM and LBM commensurate with decreases in circulating androgen-related markers. Moreover, these agents negatively impacted BMD, blood lipids, and liver enzymes with minimal effects on renal and hematological markers. FSH and LDL were disrupted 4 weeks after cessation of use. Additionally, these data indicate intramuscular AR content is suppressed, while intramuscular androgens may be elevated with use. Further human research is warranted to better understand the health effects and intramuscular dynamics of these anabolic agents.

**Assessment of Sport Nutrition Knowledge in NCAA Division III College Athletes**

Dylan J. Klein^a^, Kaitlyn Eck^b^, Alexandria Caljean^b^, Amanda Murphy^a^, Brianna Pellegrino^a^, Alan Walker^c^, Joseph Pellegrino^d^, Daniel J. Freidenreich^e^

^a^Department of Health and Exercise Science, Rowan University, Glassboro, NJ 08028, USA; ^b^Department of Nutrition and Dietetics, Marywood University, Scranton, PA 18509, USA; ^c^Department of Exercise Science, Lebanon Valley College, Annville, PA 17003, USA; ^d^Department of Health and Human Performance, Scranton University, Scranton, PA 18510. USA; ^e^Department of Exercise and Sport Science, University of Wisconsin-La Crosse, La Crosse, WI 54601, USA

Corresponding author: kleind@rowan.edu

**Background**: A major component of optimizing sport performance is a well-chosen nutrition program. College athletes have been shown to exhibit poor energy and micronutrient intakes that can decrease performance and increase the risk of poor recovery and injury. One postulated mechanism for poor nutrition is a lack of adequate sport nutrition knowledge (SNK). To date, no studies have evaluated the state of SNK in NCAA Division III college athletes using an appropriate, validated assessment tool. Therefore, the purpose of this study was to assess the SNK of NCAA Division III college athletes using an appropriate, validated SNK instrument (SNKI).

**Methods**: The SNKI [1] consisted of 49 questions, broken into six sections: carbohydrate (11), protein (9), fat (7), hydration (7), micronutrients (7), and weight management (8). Scoring was as follows: +1 for each correct answer, −1 for each incorrect answer, and +0 for ‘Don’t know’. A ‘% correct’ score was also calculated by dividing the total number of correct responses by the total number of questions for the entire instrument. Sex differences were analyzed using independent *t*-tests. Significance was set at *p* < 0.05.

**Results**: Knowledge scores for the entire 49-item questionnaire (*n* = 189 females, *n* = 149 males) ranged from −38 to 49, with a mean score of 6.39 ± 8.86. For the carbohydrate section, scores ranged from −11 to 11, with a mean score of 2.48 ± 2.73. For the protein, fat, hydration, micronutrients, and weight managements sections, scores ranged from −9 to 9 (mean score of −0.77 ± 2.85), from −5 to 7 (mean score of 0.83 ± 1.81), from −5 to 7 (mean score of 1.18 ± 1.94), from −5 to 7 (mean score of 0.94 ± 2.02), and from −6 to 8 (mean score of 1.72 ± 2.49), respectively. A ‘% correct’ score for the entire survey was 18.01% ± 36.9%. There were no significant sex differences (*p* > 0.05) for total SNK, carbohydrate, protein, fat, micronutrients, or weight management categories. There was a significant sex difference (*p* = 0.016) for hydration knowledge, with females scoring higher than males (1.41 ± 1.84 *vs*. 0.89 ± 2.02, respectively). There was no significant sex difference for ‘% correct’ scores for the entire survey (17.52% ± 35.76% males, 18.51 ± 37.78% females; *p* = 0.340).

**Conclusions**: These results highlight that NCAA Division III college athletes exhibit a low level of overall SNK, with no differences between sexes. Females, however, did score higher than males for hydration-specific nutrition knowledge.

**References**: ^1^Karpinski CA, Dolins KR, Bachman J: Development and validation of a 49-item sports nutrition knowledge instrument (SNKI) for adult athletes. *Top Clin Nutr* 2019, 34:174-185.

**Caffeine, Methylliberine, and Theacrine Improves Vigilance Without Attenuating Marksmanship in a Tactical Population**

Harry P. Cintineo^a^, Marissa L. Bello^b^, Alexa J. Chandler^a^, Thomas D. Cardaci^a^, Bridget A. McFadden^a^, and Shawn M. Arent^a^

^a^Department of Exercise Science, University of South Carolina, Columbia, SC 29208, USA; ^b^Department of Kinesiology, Mississippi State University, Mississippi State, MS 39762, USA

**Background**: Vigilance and marksmanship are important aspects of tactical performance. Nutritional strategies, such as stimulant supplementation, have been shown to maintain vigilance and marksmanship during periods of sleep deprivation, and a recent systematic review concluded that moderate doses of caffeine, a methylxanthine, improve marksmanship RT without affecting accuracy. The purpose of this study was to assess the effects of tetramethylurates (methylliberine and theacrine) in addition to caffeine on both vigilance and marksmanship performance in a tactical population.

**Methods**: Tactically trained males (n = 48) participated in this double-blind, randomized, placebo-controlled trial and were assigned to: caffeine only (300 mg; CAF), a combination of caffeine (150 mg), methylliberine (100 mg), and theacrine (50 mg; CMT), or placebo (PLA). Following familiarization, participants underwent a session consisting of two rounds of testing after consuming the supplement. Each round consisted of 30-min leisurely reading, 30-min vigilance protocol as a go/no-go reaction task with arithmetic on a D2 reaction board (Dynavision International LLC, Cincinnati, OH), then two bouts of a whole-body dynamic movement task and a 16-target marksmanship task with a tactical reload using a simulator (Smokeless Range, Laser Ammo, Great Neck, NY) with a laser-modified airsoft pistol. Targets were scaled to 15 m for the first round and 30 m for the second round. Vigilance- and marksmanship-related outcomes were converted to Z-scores in order to calculate composite performance scores at each timepoint. Linear mixed-effects models were used to analyze differences between groups over time with a random intercept for subject ID. If a significant main effect or interaction was found, post-hoc tests with a Bonferroni adjustment were performed (α = 0.05).

**Results**: A Group-by-Time interaction was found for the vigilance composite score which consisted of RT, correct decisions, and correct math responses (P = 0.019). Post-hoc tests showed that vigilance Z-score did not change for CAF and CMT (P > 0.2) but decreased for PLA (P = 0.028). No Group effects or interactions were observed for marksmanship.

**Conclusions**: The results show that both CAF and CMT attenuate decrements in a vigilance task without negatively affecting marksmanship in a tactical population. Similar to previous research, the stimulants provided allowed participants to maintain RT and mental acuity during the vigilance task while the placebo group exhibited decrements. Despite a lower caffeine dose, the addition of tetramethylurates resulted in similar tactical performance outcomes as caffeine only, which is even more noteworthy given previous literature suggesting more favorable hemodynamic responses and reduced habituation with these compounds.

**Acknowledgments**: This study was funded by Compound Solutions, Inc.

**Trial Registration**: ClinicalTrials.gov (NCT03937687)

**A Combination of Caffeine, Methylliberine, and Theacrine Elicits Different Hemodynamic Responses than Caffeine Alone During Simulated Tactical Tasks**

Alexa J. Chandler^a^, Harry P. Cintineo^a^, Bridget A. McFadden^a^, Marissa L. Bello^b^, Thomas D. Cardaci^a^, Caroline S. Vincenty^a^, Shawn M. Arent^a^

^a^Department of Exercise Science, University of South Carolina, Columbia, SC 29208, USA; ^b^Department of Kinesiology, Mississippi State University, Mississippi State, MS 39762, USA

Corresponding author: sarent@mailbox.sc.edu

**Background**: Caffeine is an ergogenic aid used to enhance cognitive and physical performance. While caffeine, a methylxanthine, is known to increase heart rate (HR) and blood pressure (BP), initial evidence for tetramethylurates (i.e. theacrine, methylliberine) suggests minimal impacts on hemodynamics. When these compounds are taken with caffeine, they appear to act synergistically but the lower caffeine content may limit the hemodynamic responses seen with caffeine alone. The purpose of this double-blind randomized placebo-controlled trial was to determine hemodynamic responses to a supplement containing a combination of caffeine, theacrine, and methylliberine versus caffeine only and a placebo at rest and during tactical simulation tasks.

**Methods**: Tactically trained males (n = 48; age = 27.1 ± 9.8 y) were randomly assigned to consume caffeine (CAF; 300 mg), combination (CMT; 50 mg caffeine + 100 mg methylliberine + 50 mg theacrine) or a placebo (PLA). Systolic (SBP) and diastolic (DBP) BP were measured prior to consuming the supplement (T0) and again at six subsequent timepoints (T1-T6), which included: 30-min rest (T1), 30-min vigilance task (T2), and whole-body dynamic movement and marksmanship tasks (T3). All tasks were repeated starting with the 30-min rest (T4-T6). HR was continuously recorded and averages during each task were calculated (T1-T6). Linear mixed-effects models were used to analyze differences between groups over time. Post-hoc tests with Bonferroni adjustments were performed to determine specific differences (α = 0.05).

**Results**: There were significant group effects for BP (P < 0.03). Overall, CAF elicited higher SBP (+11.6 mmHg; P < 0.01) and CMT (+10.7 mmHg; P < 0.01) compared to PLA, but there were no differences between CAF and CMT (P = 1.0). DBP was higher with CAF (+7.5 mmHg; P < 0.03) than PLA, but there were no differences between any other groups (P > 0.28). Analyses also revealed time effects for BP with higher SBP at T3 and T6 and higher DBP at T2, T3, and T6 compared to T0 (P < 0.01). There was no significant group effect for HR but a significant time effect showed higher values at all subsequent time points compared to T0 (P < 0.01).

**Conclusions**: While both CAF and CMT exhibited increased SBP, only CAF showed higher DBP compared to PLA. This more favorable hemodynamic response with CMT may be attributed to the lower CAF dosage. However, CMT should be explored as an alternative ergogenic aid to CAF as prior research suggests the synergistic effects of these compounds elicit similar performance benefits to CAF alone. This may be particularly important for those sensitive to CAF or special populations such as those with hypertension.

**Acknowledgments**: This study was funded by Compound Solutions, Inc.

This study was registered on ClinicalTrials.gov (NCT03937687).

**Accounting for Total Body Water in Body Composition Assessment in Division I Collegiate Athletes: Validity & Application**

Taylor E.A. Morrison^a^, Hannah E. Cabre^a,b^, Amanda N. Gordon^a^, Abbie E. Smith-Ryan^a,b^, FISSN

^a^Applied Physiology Laboratory, The University of North Carolina, Chapel Hill, NC, 27,599, USA; ^b^Human Movement Science Curriculum, Department of Allied Health Science, University of North Carolina at Chapel Hill, Chapel Hill, NC, 27,599 USA

**Background**: Dual-energy x-ray absorptiometry (DXA) and bioimpedance analysis (BIA) are commonly used body composition techniques that rely on standard assumptions for fat-free body mass, which differs among athletes. Accounting for total body water (TBW) and bone mineral density from a DXA-derived four compartment (DXA-4C) model may be more accurate than DXA or BIA alone, while requiring little extra time. The purpose of this evaluation was to compare the agreement between DXA and BIA to a DXA-4C criterion method for body composition assessment in division I collegiate athletes.

**Methods**: A sample of 363 male (n = 197; cross-country and football) and female (n = 166; cross-country, field hockey, gymnastics, lacrosse, and soccer) NCAA division I collegiate athletes (Mean ± standard deviation [SD], Age: 19.3 ± 1.2 yrs; Body Mass: 84.3 ± 27.4 kg) were evaluated for body composition to determine fat mass (FM), fat-free mass (FFM), and body fat percentage (BF%). Body composition was measured via DXA and BIA separately, in comparison to a DXA-4C model using body volume from previously published coefficients, and TBW from BIA. Validity statistics including constant error (CE; DXA-4C – DXA/BIA), total error (TE), and standard error of the estimate (SEE) were completed to compare DXA and BIA to DXA-4C. Results were classified using Heyward and Wagner standards for evaluating prediction errors.

**Results**: For the total sample with outliers (± 2 SD) removed, DXA in comparison to DXA-4C model resulted in ideal to good TE and SEE for measures of FM (TE: 3.98 kg; SEE: 2.24 kg), FFM (TE: 3.44 kg; SEE: 0.57 kg), and BF% (TE: 3.32%; SEE: 2.24%). BIA resulted in ideal TE and SEE for measures of LM (TE: 1.72 kg; SEE: 1.51 kg), and ideal to very good for FM (TE: 3.04 kg; SEE: 2.21 kg) and BF% (TE: 3.11%; SEE: 2.92%). The greatest prediction errors were found for DXA FM estimates (TE: 4.40 kg) and BF% (TE: 4.35%) amongst females, and DXA LM estimates (TE: 4.03 kg) in males. Validity data is presented in Table 1.

**Table 1.** Validity Statistics for DXA-4C compared to DXA and BIA (DXA-4C-DXA/BIA)

	**Total Sample**			**Male**			**Female**		
**DXA**									
**Absolute Error**	**CE**	**TE**	**SEE**	**CE**	**TE**	**SEE**	**CE**	**TE**	**SEE**
**FM (kg)**	−2.81	3.98^d^	2.24^b^	−1.72	3.61^d^	2.25^a^	−4.12	4.40^e^	2.25^a^
**LM (kg)**	3.30	3.44^c^	0.57^a^	3.91	4.03^e^	0.60^a^	2.62	2.65^c^	0.62^a^
**BF%**	−1.96	3.32^c^	2.54^b^	−1.28	2.87^b^	2.62^b^	−3.92	4.35^e^	2.68^b^
**BIA**									
**FM**	−0.80	3.04^c^	2.21^a^	0.74	3.11^c^	2.21^a^	−2.66	2.96^b^	2.17^a^
**LM**	−0.78	1.72^a^	1.51^a^	−0.60	1.85^a^	1.58^a^	−0.93	1.60^a^	1.67^a^
**BF%**	−0.88	3.11^c^	2.92^b^	0.47	2.77^b^	2.95^b^	−3.23	3.63^d^	2.97^b^

Note: Estimates were derived as DXA-4C – DXA; FM, fat mass; LM, lean mass; %BF, percent body fat; CE, constant error; TE, total error; SEE, standard error of the estimate. Classifications according to Heyward and Wagner: ^a^, Ideal; ^b^, Excellent; ^c^, Very Good; ^d^, Good; ^e^, Fairly Good; ^f^, Fair; ^g^, Poor.

**Conclusions**: With the DXA-4C model as a criterion, BIA may provide superior validity in comparison to DXA in all body composition measures. DXA was found to be an accurate approach, but the addition of TBW significantly improved its validity, particularly in athletes. Use of a DXA-4C approach increases the validity for female athletes and for LM estimates in male athletes.

**The Potential Synergistic Effect of Combined Blood Flow Restriction Training and Betaine Supplementation on One-leg Press Performance and Exercise-associated Lactate Concentrations**

Steven B. Machek^a^, Jeffery L. Heileson^a^, Dillon R. Harris^a^, Dylan T. Wilburn^a^, Mitchell C. Cholewinski^a^, Christopher D. Hulsey^a^, LesLee K. Funderburk^b^, Tracey N. Sulak^c^, Jason M. Cholewa^d^, & Darryn S. Willoughby, FISSN^e^

^a^Department of Health, Human Performance, & Recreation, Robbins College of Health and Human Sciences, Baylor University, Waco, TX; ^b^Human Sciences and Design, Robbins College of Health and Human Sciences, Baylor University, Waco, TX; ^c^Educational Psychology Department, School of Education, Baylor University, Waco, TX; ^d^Exercise Physiology Department, University of Lynchburg, Lynchburg, VA; ^e^School of Exercise and Sport Science, University of Mary Hardin-Baylor, Belton, TX

Corresponding author: dwilloughby@umhb.edu

**Background**: Blood flow restriction (BFR) training is commonly employed to elicit increases in skeletal muscle hypertrophy, largely due to acute hypoxia-mediated metabolic stress and a subsequent extra-to-intracellular fluid shift (ultimately normalizing osmotic stress). Betaine supplementation may therefore potentially augment BFR training via osmolyte-associated effects on protein stabilization and/or through attenuating hypotonic scenarios. Therein, the purpose of this investigation was to compare the impacts of concurrent betaine supplementation on one-leg press performance and lactate concentrations, whilst employing both low-load BFR and standard high-load resistance exercise.

**Methods**: Eighteen apparently healthy males (25 ± 5y) were randomized in double-blind fashion to supplement 6 g/day of either betaine anhydrous or cellulose placebo for 14-days. Following one-repetition maximum assessments on day 12 of supplementation, all participants performed four standardized sets, as well as two sets to muscular failure of one-leg press exercise on both legs in a randomized, counter-balanced, and crossover design. Specifically, one leg performed standard high-load (HL; 70%1RM) exercise, whereas the contralateral limb underwent partially occluded BFR (LLO; 20%1RM) training at 80% arterial occlusion pressure. Each leg’s total repetitions completed was used to calculate total load-volume and toe-tip lactate concentrations were sampled before exercise (PRE), as well as immediately (POST0), 30-minutes (POST30M), and 3-hours (POST3H) post-exercise. All dependent variables were analyzed using factorial (two-and-three way) mixed model ANOVA performed at a significance level of p < .05.

**Results**: In all instances, there were no significant main effects for supplement (p > .05). Analysis revealed significant main effects for total repetitions (p > .001; η_p_^2^ = .729) and total load-volume completed (p < .001; η_p_^2^ = .875), whereby LLO accrued significantly more repetitions (p < .001) but less load-volume (p < .001) relative to HL. Furthermore, there were significant condition (p < .001; η_p_^2^ = .814) and time (p < .001; η_p_^2^ = .908) main effects for lactate changes from baseline, whereby time-collapsed HL had higher concentrations versus LLO (p < .001) and both POST0 and POST30M were significantly higher (both p < .001) than POST3H, ultimately with no differences between the former two time points (p = .272). Lastly, there was a significant condition x time interaction (p < .001; η_p_^2^ = .237), illustrating higher lactate concentrations for HL versus LLO at POST3H (p < .001).

**Conclusions**: Given the findings of this acute intervention, it appears betaine supplementation does not potentiate BFR *or* standard high-load resistance training with respect to one-leg press performance nor differences in lactate concentrations. However, these equivocal results may stand overturned by future research that aims to assess more mechanistic outcomes (cellular-level changes) and/or employs a combined BFR-betaine modality in a longitudinal model.

**Acknowledgments**: The authors would like to thank Dr. Mike DeBord from B3 Sciences and Jack Owoc from Vital Pharmaceuticals for generously donating the experimental cuffs and supplement, respectively, to assist in the completion of this investigation.

**Impacts of Varying Blood Flow Restriction Cuff Size and Material on Arterial, Venous, and Calf Muscle Pump-Mediated Blood Flow**

Dillon, R. Harris^a^, Steven B. Machek^a^, Jeffery L. Heileson^a^, Dylan T. Wilburn^a^, Jeffrey S. Forsse^a^ & Darryn S. Willoughby^b^, FISSN

^a^Department of Health, Human Performance, & Recreation, Robbins College of Health and Human Sciences, Baylor University, Waco, TX; ^b^School of Exercise and Sport Science, University of Mary Hardin-Baylor, Belton, TX

**Corresponding author**: Steven_machek2@baylor.edu

**Background**: Blood flow restriction (BFR) training is commonly employed to elicit hypertrophy and commensurate strength gains via full venous and partial arterial occlusion. However, if used haphazardly and/or without standardization (i.e. assessing arterial occlusion pressure [AOP]) on the proximal limb(s), this modality can range from ineffective to potentially dangerous. Therefore, the purpose of this study was *1)* determine the viability of multiple sizes of a novel multi-chambered pneumatic BFR cuff product to reliably attain AOP, and *2)* investigate any blood flow-associated differences between the aforementioned cuffs and a commonly used tourniquet-style cuff.

**Methods**: Twenty apparently healthy males (18-40y) adorned either a commonly employed wide rigid (WR) cuff, as well the largest (NE) and manufacturer-recommended sizes (NER) of a novel narrow elastic cuff in randomized, counter-balanced, and crossover fashion. After providing consent, subjects rested in a supine position for 10 minutes, both prior to and between cuff conditions, before ultrasound assessment of the participant’s right leg. AOP was determined and subsequently reduced to 80% for (both mean and peak) arterial, venous, and calf muscle pump-mediated blood flow (vs rest) comparison. Subjective discomfort between rest and cuff conditions was additionally assessed utilizing the Borg CR10+ scale. All dependent variables were analyzed via factorial one-way repeated measures ANOVA. Furthermore, data failing to meet normality assumptions were assessed via Friedman’s ANOVA. All analyses were performed at a significance level of p < .05.

**Results**: Analyses revealed a significant effect for cuff type on AOP (p < .001; η_p_^2^ = .907), whereby WR was significantly lower than both NE (p < .001) and NER (p < .001), of which the latter two did not differ (p = 1.000). Compared with rest, there were no statistically significant differences between cuffs for either arterial (mean p = .291; peak p = .115) nor calf muscle pump-mediated (mean p = .565; peak p = .368) blood flow. Unsurprisingly, no participants demonstrated venous blood flow at 80% AOP. Lastly, nonparametric analyses revealed a significant effect for subjective discomfort (p < .001; Kendall’s W = .575), whereby all cuff conditions displayed greater CR10+ values (p < .001 in all cases) versus rest without significant differences between cuffs.

**Conclusions**: These findings support the viability of a novel narrow elastic BFR cuff design. Specifically, these cuffs produced similar blood flow parameter changes, whilst also reliably reaching AOP amidst similar discomfort levels. This investigation is also the first to document calf muscle pump-mediated blood flow in general and amongst all cuff types, potentially suggesting this phenomenon is an important factor facilitating BFR-associated exercise duration and subsequent adaptation.

**Acknowledgments**: The authors would like to thank Dr. Mike DeBord and B3 Sciences for generously donating their cuff products to assist in the completion of this investigation.

**Decreases in Body Esteem Following Body Composition Testing are Dependent on Sex and Percent Body Fat**

Geoffrey M Hudson, Caitlyn R Hauff, Alyssa M. Zediker, Andrew P. Theodore

Department of Health, Kinesiology, and Sport, University of South Alabama, Mobile, AL, 36,688, USA

Corresponding author: GHudson@southalabama.edu

**Background**: Optical body fat scanners and bioelectrical impedance devices have made body composition testing more accessible and affordable which allows people to more easily track body fat (%BF) changes. It has been demonstrated, however, that body composition testing can negatively impact body esteem. These data highlight how exposure to body fat results from a DXA (dual x-ray absorptiometry) scan and the image from an optical body fat scanner can impact how different groups feel about their bodies.

**Methods**: Participants (n = 64; 31% male; age = 27.9 ± 10.4 years; BMI = 25.9 ± 5.0 kg-m^−2^; body fat = 32.4 ± 9.5%) completed the Body Esteem Scale (BES) [1] at three different time points: 1) baseline measurement, 2) after receiving numerical body fat results from a DXA scan, and 3) after viewing their 3D image from an optical body fat scanner. The BES includes three subscales (Appearance, Weight, Attributes) that include 15 positive items and 9 negative items. Participants’ data were subdivided by gender, normal/overweight by BMI, and into quartiles for %BF based on age-matched percentiles from DXA.

**Results**: ANOVA with repeated measures indicated significant changes for both the positive (p = .003) and negative items (p < .0001) as well as all three subscales (Appearance, Weight, Attributes; p = .001, .005, .025, respectively) after body composition testing. Scores decreased on 7 (of 14) positive items and increased on 5 (of 9) negative items. Females scored significantly lower than males on four negative items: ‘My looks upset me’, ‘I feel ashamed of how I look’, ‘Weighing myself depresses me’, ‘My weight makes me unhappy’. Overweight individuals and those in the highest quartile for %BF scored significantly lower on three items regarding weight satisfaction. Effect sizes (η_p_^2^) were largest for an increase in these items: ‘I feel ashamed of how I look’ (η_p_^2^ = .162), ‘My weight makes me unhappy’ (η_p_^2^ = .127), and ‘My looks upset me’ (η_p_^2^ = .146) and a decrease in: ‘I’m pretty happy about the way I look’ (η_p_^2^ = .109).

**Conclusions**: Receiving body composition results from a DXA and an optical body fat scanner negatively impacts body esteem and can influence some subgroups of people more than others. More research is needed to understand how individuals are affected by these data in order to provide best practice when delivering results about sensitive information, like body fat, as this could result in decreasing self-esteem and potential adoption of maladaptive weight control behaviors.

**Acknowledgments**: This project was funded by the University of South Alabama CEPS Research Development Grants Program.

**References**: ^1^Mendelson BK, Mendelson MJ, White DR: Body-esteem scale for adolescents and adults. *Journal of Personality Assessment* 2001, 76(1):90-106.

**Acute Paraxanthine Ingestion Improves Cognition, Short-Term Memory and Helps to Sustain Attention**

Megan Leonard^a^, Choongsung Yoo^a^, Dante Xing^a^, Tori Jenkins^a^, Kay Nottingham^a^, Broderick Dickenson^a^, Joungbo Ko^a^, Drew Gonzalez^a^, Mark Faries^a,b^, Wesley Kephart^c^, Martin Purpura^d^, Ralf Jäger, FISSN^d^, Shawn D. Wells, FISSN^e^, Ryan Sowinski^a^, Christopher J. Rasmussen^a^, Richard B. Kreider, FISSN^a^

^a^Exercise & Sport Nutrition Lab, Human Clinical Research Facility, Texas A&M University, College Station, TX 77843, USA; ^b^Texas A&M AgriLife Extension, Texas A&M University, College Station, TX 77843, USA; ^c^Department of Health Science, University of Wisconsin – Whitewater, Whitewater, WI 53190, USA; ^d^Increnovo LLC, Milwaukee, WI 53202, USA; ^e^Ingenious Ingredients L.P., 2560 King Arthur BLVD Suite 124-74, Lewisville, TX 75056, USA

**Background**: About 80% of ingested caffeine (CA) is converted to paraxanthine (PX) in the body. Some studies suggest PX may possess nootropic effects with less unfavorable effects on the central nervous system compared to caffeine. The purpose of this study was to examine the effects acute PX ingestion on markers of cognition, executive function and psychomotor vigilance.

**Methods**: In a randomized, double-blind, placebo-controlled, crossover, and counterbalanced manner, 12 healthy male and female subjects (24 ± 5 years, 170.0 ± 12 cm, 72.9 ± 19 kg, 24.8 ± 4 kg/m^2^) were randomly assigned to consume a placebo (PL) or 200 mg of PX (ENFINITY™, Ingenious Ingredients, L.P.). Participants completed stimulant sensitivity and side effect questionnaires and then performed the Berg-Card Sorting task test (BCST) that is an executive function test that assesses long thought, including reasoning, learning, executive control, and attention shifting; the Go/No-Go test (GNG) that assesses sustained attention and response control through reaction time and accuracy to visual stimuli; the Sternberg task test (STT) that assesses short-term/working memory involving cognitive control processes using reaction time and accuracy; and, the Psychomotor Vigilance Task Test (PVTT) that assesses sustained attention reaction times through responses to visual stimuli. Participants then ingested one capsule of PL or PX treatments with 8 ounces of water. Participants completed side effects and cognitive function tests after 1, 2, 3, 4, 5, and 6 hours of after ingestion of the supplement. After 7 days participants then repeated the experiment while consuming the alternative treatments. Data were analyzed by a General Linear Model (GLM) univariate analyses with repeated measures using weight as a covariate and by assessing mean and percent changes from baseline with 95% Confidence Intervals (CI’s) expressed as means (LL, UL).

**Results**: PX decreased BCST Errors (PX −4.7 [−0.2, −9.20], p = 0.04; PX −17.5% [−36.1, 1.0], p = 0.06) and Perseverative Errors (PX −2.2 [−4.2, −0.2], p = 0.03; PX −32.8% [−64.4, 1.2], p = 0.04) at hour 6. Analysis of GNG results revealed some evidence that PX ingestion better maintained Mean Accuracy and measures of response time over the 6-hour period suggesting better sustained attention (e.g. PX −25.1 [−52.2, 1.9], p = 0.07). There was also evidence that PX ingestion improved STT 2-letter length Absent and Present Reaction Time over time as well as improved 6-letter length Absent Reaction Time after 2 hours (PX −86.5 ms [−165, −7.2], p = 0.03; PX −9.0% [−18.1, 0.2], p = 0.05) suggesting that PXN enhanced the ability to store and retrieve random information from short-term memory of increasing complexity to a greater degree. Finally, a moderate treatment x time effect size (ηp^2^ = 0.08) was observed in PVTT Trial 2 Reaction Time with reaction time where PX sustained vigilance during Trial 2 after 2-hours (PX 840 ms [103, 1576], p = 0.03) and 4-hours (PX 1466 ms [579, 2353], p = 0.002) compared to PL. Additionally, as testing progressed, response time improved during the 20 trials and over the course of the 6-hour experiment in the PX treatment whereas it significantly increased in the PL group suggesting that PX helped sustain attention (i.e. maintained reaction times, prevention of mental fatigue).

**Conclusions**: Acute PX ingestion (200 mg) may affect some measures of short-term memory, reasoning, response time to cognitive challenges, and as well as help sustain attention.

**Acknowledgments**: This study was funded as a fee for service project to the Human Clinical Research Facility research by Ingenious Ingredients, LP (ING2). RJ, SDW and MP are co-owners of ING2 and have been named as inventors for the use of PX by ING2.

**Dose-Ranging Study of Paraxanthine Ingestion on Cognition, Executive Function, and Psychomotor Vigilance**

Broderick Dickerson^a^, Dante Xing^a^, Choongsung Yoo^a^, Tori Jenkins^a^, Kay Nottingham^a^, Drew Gonzalez^a^, Megan Leonard^a^, Joungbo Ko^a^, Mark Faries^a,b^, Wesley Kephart^c^, Martin Purpura^d^, Ralf Jäger, FISSN^d^, Shawn D. Wells, FISSN^e^, Ryan Sowinski^a^, Christopher J. Rasmussen^a^, Richard B. Kreider, FISSN^a^

^a^Exercise & Sport Nutrition Lab, Human Clinical Research Facility, Texas A&M University, College Station, TX 77843, USA; ^b^Texas A&M AgriLife Extension, Texas A&M University, College Station, TX 77843, USA; ^c^Department of Health Science, University of Wisconsin – Whitewater, Whitewater, WI 53190, USA; ^d^Increnovo LLC, Milwaukee, WI 53202, USA; ^e^Ingenious Ingredients L.P., 2560 King Arthur Blvd, Suite 124-74, Lewisville, TX 75056, USA

**Background**: Acute ingestion of 200 mg of paraxanthine (PX) affects measures of short-term memory, reasoning, response time to cognitive challenges, and as well as help sustain attention. The optimal and minimal effective dose of PX is currently unknown. The purpose of this study was to assess the efficacy and safety of different doses of PX on markers of cognition, executive function and psychomotor vigilance to one week of continued supplementation.

**Methods**: In a randomized, double-blind, placebo-controlled, crossover, and counterbalanced manner, 12 healthy male and female subjects (22.7 ± 4 years, 165 ± 7 cm, 66.5 ± 11 kg, 24.4 ± 3 kg/m^2^) were assigned to ingest 200 mg of a placebo (PLA), 50 mg of PX (ENFINITY™, Ingenious Ingredients, L.P.) + 150 mg PLA, 100 mg PX + 100 mg PLA, or 200 mg of PX. Participants completed stimulant sensitivity and side effect questionnaires and donated a fasting blood sample. Participants then performed the Berg-Card Sorting task test (BCST) that is an executive function test that assesses long thought, including reasoning, learning, executive control, and attention shifting; the Go/No-Go test (GNG) that assesses sustained attention and response control through reaction time and accuracy to visual stimuli; the Sternberg task test (STT) that assesses short-term/working memory involving cognitive control processes using reaction time and accuracy; and, the Psychomotor Vigilance Task Test (PVTT) that assesses sustained attention reaction times through responses to visual stimuli. Participants then ingested on capsule of PLA or PX treatments with 8 ounces of water. Participants completed side effects and cognitive function tests after 1, 2, 3, 4, 5, and 6 hours of after ingestion of the supplement. Participants continued ingesting one dose a day of the assigned supplement and then returned to the lab to donate a fasting blood sample. After a 7-day washout period, participants returned to the lab to repeat the experiment. Participants this protocol two additional times until all fore treatments were assessed. Data were analyzed by a General Linear Model (GLM) univariate analyses with repeated measures using weight as a covariate and assessing mean and percent changes from baseline with 95% Confidence Intervals (CI’s).

**Results**: STT 4-Letter Length Present Reaction Time tended to differ among groups (p-0.06). Assessment of mean changes from baseline with 95% CI’s revealed several significant differences among treatments in BCST Correct Responses, Preservative Errors (PEBL), and Preservative Errors (PAR Rules) providing some evidence that PX at varying doses enhanced thought, reasoning, learning, executive control, and attention shifting. There was also evidence of significant differences among treatments in GNG Tasks in Mean Accuracy and response time markers under various conditions assessed providing some evidence that PX influences help sustain attention. Likewise, there were significant differences among treatments at several timepoints of increasing complexity among STT variables assessed suggesting that PX enhanced the ability to store and retrieve random information from short-term memory of increasing complexity to a greater degree. Finally, there was evidence from the PVTT assessment that response time improved over the series of 20 trials assessed as well as over the course of the 6-hour experiment in the PX treatment suggesting that PX helped sustain attention. Benefit compared to PLA were seen with each dose studied but more consistent effects appeared to be at 100 m and 200 mg doses. No significant differences were observed in side effects or standard clinical chemistry panels.

**Conclusions**: Results provide some evidence that acute ingestion of 100 mg and 200 mg of PX may affect some measures of short-term memory, reasoning, response time to cognitive challenges, and help sustain attention and that 7-days of PX ingestion is not associated with any clinically significant side effect.

**Acknowledgments**: This study was funded as a fee for service project to the Human Clinical Research Facility research by Ingenious Ingredients, LP (ING2). RJ, SDW and MP are co-owners of ING2 and have been named as inventors for the use of PX by ING2.

**Changes in Body Composition, Muscle Thickness, and Strength Following 9 Weeks of High- or Low-Load Resistance Training**

Marissa L. Bello^1^, Derick A. Anglin^1^, Harold A. Joseph^2^, Zachary M. Gillen^1^, JohnEric W. Smith^1^

^a^Department of Kinesiology, Mississippi State University, Mississippi State, MS, 39,762, USA; ^b^Department of Applied Physiology and Kinesiology, University of Florida, Gainesville, FL, 32,611, USA

Corresponding author: mlb1221@msstate.edu

**Background**: Programming resistance training to optimize adaptations is a crucial part to achieve goals in not only athletes but the everyday lifter. Higher loads are hypothesized to improve strength more, whereas lower loads prioritize hypertrophy. However, most of the research has not used a true ‘strength’ range for training load. Therefore, the purpose of this study was to observe and compare changes in body composition, muscle thickness, and muscular strength following 9 weeks of resistance training at a high- or low-load.

**Methods**: Six recreationally trained male lifters were recruited for this study (M_age_ = 21.8 ± 3.8 yrs; M_weight_ = 81.0 ± 15.5 kg). Participants were split into training at either high- (85% 1-RM; n = 3) or low-load (30% 1-RM; n = 3) and completed 3 sessions per week of a whole-body workout (squat, deadlift, bench, row, biceps, triceps) for 9 weeks. Each session included three working sets of repetitions to failure. At baseline (T1), and every three weeks (T2-T4), body composition (%BF), and muscle thickness (biceps, triceps, chest, quadriceps, hamstring) were assessed. Predicted 1-RM was conducted pre- and post-training program. Participants were asked to make no dietary changes throughout the program and 3-day recalls were used at each timepoint. RM-ANOVAs with univariate follow-ups were conducted to analyze changes in muscle thickness, body composition, and strength. Significance was set at P < 0.05.

**Results**: There was a significant time effect for muscle thickness of the biceps, chest, hamstring, and quadriceps (P < 0.03), with no group differences. Further, there were no significant differences in %BF. There was a main time effect in all lifts performed. A time-by-group interaction was seen in squats (P = 0.009) and biceps (P = 0.019) and approached significance in triceps and deadlift (P = 0.061), with 85% 1-RM increasing more than the 30% group. There were no significant changes in dietary intake (P > 0.05).

**Conclusions**: Despite the higher loads in the 85% 1-RM group, only the predicted 1-RM for squats and biceps demonstrated a time-by-group interaction. Further, both groups demonstrated increases in all lifts apart from squats, demonstrating similar strength improvements over the course of the program. While all groups exhibited increases in muscle thickness, the lack of significance between groups indicates similar hypertrophy occurred regardless of load and volume differences.

**Impacts of Weight and Position on Treadmill Sprinting Speeds in Football Players**

Corey A. Peacock^a^, Nyles Rife^a^, Jose Antonio^a^

^a^Department of Health and Human Performance, Nova Southeastern University, Ft. Lauderdale FL, USA

Corresponding author: jose.antonio@nova.edu

**Background**: Strength and conditioning has become an essential, off-season element of preparation for professional football players. Incorporated in these strength and conditioning protocols includes weightlifting, plyometric training, agility drills, and sprinting. Current trends in sprint training have included the application of resistance utilizing a non-curved, non-motorized treadmills. Previous research has demonstrated correlations between non-curved, non-motorized treadmills and specific aspects of the 36.58 m dash (40-yard). There is a lack of research exploring the impacts of weight and position in regards to treadmill sprinting speeds. Therefore, we compared sprinting speeds during different settings of non-curved, non-motorized treadmills between different positions. We also assessed the relationship between weight and peak sprinting speeds.

**Methods**: Thirteen male, professional football players (26.9 ± 3.1 years, 183.2 ± 8.4 cm, 97.6 ± 12.2 kg), with previous training utilizing a non-curved, non-motorized treadmill were tested during off-season training. The football players were randomized and counterbalanced between four different bouts of sprinting on a non-curved, non-motorized treadmill (SHREDmill, Boca Raton, FL, USA). The sprint bouts included settings of a 15% incline and a resistance of eight (15R8), a 15% incline and a resistance of five (15R5), a 20% incline and a resistance of three (20R3), and a 20% incline with a resistance of 1 (20R1). Sprint speed (m/s) was documented for each of the four non-curved, non-motorized bouts.

**Results**: We found no significant differences in sprint speed between position groups (Skill vs. Big Skill) at 15R8 (P = .304), 15R5 (P = .118), 20R3 (P = .542), and 20R1 (P = .295). Correlation analysis demonstrated significant strong positive relationship between weight and 15R8 (r = 0.784, P = 0.002). Correlation analysis also demonstrated a strong positive relationship between weight and 15R5 (r = 0.805, P < 0.00). No other relationships existed (P ≥ 0.05).

**Conclusions**: Our data demonstrates non-significant differences between position groups and sprint bouts utilizing a non-curved, non-motorized treadmill in football players; however, there is a relationship between weight and two bouts of incline/resistance settings (15R8, 15R5). These findings may provide better application to non-curved, non-motorized resistance settings in relation to football player weight.

**Effects of Arginine Silicate and Inositol Ingestion on Cognitive and Executive Function in Gamers**

Drew Gonzalez^a^, Ryan Sowinski^a^, Dante Xing^a^, Choongsung Yoo^a^, Tori Jenkins^a^, Kay Nottingham^a^, Broderick Dickenson^a^, Megan Leonard^a^, Joungbo Ko^a^, Megan Humphries^a^, Mark Faries^a,b^, Wesley Kephart^c^, Christopher J. Rasmussen^a^, Richard B. Kreider, FISSN^a^

^a^Exercise & Sport Nutrition Lab, Texas A&M University, College Station, TX 77843, USA; ^b^Texas A&M AgriLife Extension, Texas A&M University, College Station, TX 77843, USA; ^c^Department of Health Science, University of Wisconsin – Whitewater, USA

**Background**: Gaming or esports requires quick reactions, executive function, memory, and fine motor skills. Prior reports indicated that ingestion of arginine silicate (ASI) [1] improved the ability to perform complex cognitive tests requiring mental flexibility, processing speed and executive functioning. In addition, ingestion of ASI with 100 mg inositol (I) improved cognitive function in gamers after playing video games for one hour [2]. This study examined whether ASI+I ingestion prior to and following a 1-hour gaming challenge had effects on cognitive function.

**Methods**: In a double-blind, randomized, placebo controlled, and crossover trial, 26 healthy male and female experienced gamers (23 ± 5 years, 171 ± 11 cm, 73.1 ± 21 kg, 21.1 ± 5 kg/m^2^) were randomly assigned to consume 1,500 mg of ASI plus 100 mg of I (nooLVL®, Nutrition 21) or 1,600 mg of a maltodextrin placebo control (PLA). Prior to testing participants recorded their diet for 4-days, refrained from consuming atypical amounts of stimulants as well as foods high in arginine and nitrates for 72-hours, and fasted for 8 hours prior to testing. At testing participants completed stimulant sensitivity and side effect questionnaires and performed cognitive function tests (i.e. Berg-Washington Card Sorting task test, Go No-Go test, Sternberg task test, Psychomotor Vigilance task test, Cambridge Brain Sciences Reasoning and Concentration test) and a Neurotracker light reaction test (Pre-SUPP). Participants then ingested one of the two the study treatments in a randomized manner. Fifteen minutes following ingestion, participants repeated tests (Pre-Game). Participants then played their favorite video game for 1-hour and repeated the battery of tests (Post-Game). Participants observed a 7-14-day washout period and then replicated their 4-day diet, pre-experiment controls, and the experiment while consuming the alternative treatment. Data were analyzed by General Linear Model (GLM) univariate analyses with repeated measures using weight as a covariate, paired t-tests (not adjusted to weight), and mean changes from baseline with 95% Confidence Intervals (CI).

**Results**: Pairwise comparison of the Sternberg test revealed statistically significant results of treatment vs. placebo. This test specifically measures mental reasoning, reaction time and short-term memory recall with the ascending difficulty and complexity of the test, important for gamers. The analysis showed that there was significantly improved Mean Present Reaction Time (ASI+I vs. Placebo; p < 0.05). In Post-Game assessments, 4-letter Absent Reaction Time (p < 0.05), 6-letter Present Reaction Time (p < 0.01), 6-letter Absent Reaction Time (p < 0.01), Mean Present Reaction Time (p < 0.02), and Mean Absent Reaction Time (p < 0.03) were improved with ASI+I vs. placebo, suggesting that participants were able to store and retrieve random information from short-term memory of increasing complexity to a greater degree. Additionally, there was a non-significant trend after 15-min in Pre-Game Sternberg 4-letter Present Reaction time in ASI+I vs. placebo (p < 0.07). There was also evidence from assessing mean changes with 95% CI’s that ASI+I ingestion better maintained changes in Go-No-Go Mean Accuracy and Reaction Time; Psychomotor Vigilance Task Reaction Time; and, Cambridge Post-Game Visuospatial Processing and Planning. However, GLM analysis of all three time points corrected for subject body weight revealed no significant treatment x time interactions in variables, additionally, no significant differences were observed in the Berg-Washington Card Sorting test, the light tracking assessments or the frequency or severity of stimulant sensitivity side effects.

**Conclusions**: Results provide evidence that ASI+I ingestion prior to playing video games may enhance some measures of short-term memory, reaction time, reasoning, and concentration in experienced gamers. Additional research should further examine the role of ASI+I on cognition, reasoning, and memory in gamers.

**Acknowledgments**

This study was funded as a fee for service project to the Human Clinical Research Facility at Texas A&M University by Nutrition 21, Inc. (Harrison, NY, USA).

**References**

^1^Kalman, D., et al., *Randomized Prospective Double-Blind Studies to Evaluate the Cognitive Effects of Inositol-Stabilized Arginine Silicate in Healthy Physically Active Adults*. Nutrients, 2016. **8**(11).

^2^Tartar, J.L., D. Kalman, and S. Hewlings, *A Prospective Study Evaluating the Effects of a Nutritional Supplement Intervention on Cognition, Mood States, and Mental Performance in Video Gamers*. Nutrients, 2019. **11**(10).

**Metabolic Impact of Pre-Exercise Macronutrient Feeding Prior to a 60-Minute Bout of Moderate-Intensity Treadmill Exercise in Recreationally Active Females**

Kayla M. Ratliff, Anthony M. Hagele, Jessica M. Moon, Johnathan L. Boring, Kylie E. Walden, Connor J. Gaige, Richard A. Stecker, Kyle L. Sunderland, Petey W. Mumford, Chad M. Kerksick

Exercise and Performance Nutrition Laboratory, College of Science, Technology, and Health, Lindenwood University, St. Charles MO, 63,101, USA

Corresponding author: ckerkisck@lindenwood.edu

**Background**: Exercising in a fasted state continues to be a popular exercise strategy due to its purported ability to instigate higher amounts of fat oxidation. Additionally, pre-exercise feeding has been largely discouraged due to the thoughts that nutrient intake will blunt the breakdown of fat. Previous research in males performing a 30-minute bout of treadmill exercise suggested that pre-exercise protein feeding may augment fat oxidation rates. To date, limited research has examined the impact pre-exercise feeding of protein or carbohydrate has on substrate oxidation in recreationally active females. The purpose of this study was to examine the impact of pre-exercise feeding of two different types of protein or carbohydrate on changes in substrate oxidation in females.

**Methods**: Fifteen healthy, recreationally active females (32 ± 10 years, 164.8 ± 5.6 cm, 63.5 ± 9.3 kg, 23.4 ± 3.2 kg/m^2^) completed four exercise testing sessions in a randomized, double-blind, placebo-controlled, crossover fashion after an overnight fast of 8 – 10 hours. During each visit, participants ingested isovolumetric solutions containing either 25 g of whey protein, 25 g of casein protein, 25 g of carbohydrate (CHO), or a flavored non-caloric control (PLA). Thirty minutes after ingestion, participants completed 60 minutes of treadmill exercise at 15% below their ventilatory threshold. Expired gases were collected in alternating intervals (5 minutes on, 5 minutes off) throughout the entire 60-minute exercise bout, with the average of the last 2-minutes of each measurement interval utilized for analysis. Total and rate of carbohydrate and fat oxidation were calculated using standard and thermal equivalents of oxygen using the non-protein respiratory quotient (NPRQ) table.

**Results**: A significant group x time interaction was observed for respiratory exchange ratio (p = 0.008) during the exercise bout, with CHO being higher than PLA at four time points. No significant group x time interaction was observed for CHO oxidation rate (p = 0.12) or fat oxidation rate (p = 0.20) during exercise. However, CHO resulted in significantly less total fat oxidation during the exercise bout when compared to PLA (p = 0.0XX). No differences in oxidation rate or amount of oxidized nutrients were found for either of the protein conditions.

**Conclusions**: Pre-exercise feeding with either type of protein or carbohydrate did not significantly alter CHO or fat oxidation rates when compared to fasting. However, pre-exercise carbohydrate feeding did reduce the total amount of fat oxidized during a 60-minute bout of moderate-intensity exercise in recreationally trained females.

**Influence of Caffeinated and Non-Caffeinated Pre-Workout Supplements on Maximal and Rapid Isometric Strength Characteristics**

Christian Rodriguez, Matthew T. Stratton, Madelin R. Siedler, Patrick S. Harty, Jake R. Boykin, Jacob J. Green, Dale S. Keith, Sarah J. White, Abegale D. Williams, Brielle DeHaven, Grant M. Tinsley

Energy Balance & Body Composition Laboratory, Department of Kinesiology & Sport Management, Texas Tech University, Lubbock, TX, USA

Corresponding author: grant.tinsley@ttu.edu

**Background**: Multi-Ingredient pre-workout supplements (MIPS), which are commonly utilized by active individuals seeking to improve exercise performance, typically contain a blend of ingredients purported to augment performance when ingested simultaneously. Caffeine is commonly believed to be the primary ingredient responsible for several of the acute ergogenic effects of MIPS, however, stimulant-free formulations are also gaining popularity. Currently, there is a dearth of evidence comparing the effects of caffeine-containing pre-workout formulations to otherwise-similar caffeine-free versions on measures of maximal muscular performance.

**Methods**: A cohort of 24 resistance-trained individuals, including males (n = 12; body mass: 82.7 ± 7.5 kg; fat-free mass index: 21.7 ± 1.8 kg/m^2^; body fat: 18.2 ± 5.8%) and females (n = 12; body mass: 63.0 ± 10.6 kg; fat-free mass index: 16.9 ± 0.8 kg/m^2^; body fat: 26.0 ± 6.7%), completed three visits in which they ingested either a caffeinated MIPS (C), an otherwise-similar non-caffeinated MIPS (NC), or placebo in a randomized, double-blind, counter-balanced fashion. 35 minutes post-supplement ingestion, a maximal isometric squat exercise testing protocol was administered on a mechanical squat device. Specifically, upon setting the participants’ knee position at 120 degrees of knee extension, two trial isometric efforts were performed, followed by two separate maximal isometric efforts. The effects of pre-workout supplementation and biological sex on peak isometric force (PF) and rate of force development over 200 ms (RFD_200_) were examined using linear mixed effects models, which included terms for period and carryover effects and a random intercept for participant.

**Results**: PF was higher with NC (*b*: 0.32 transformed units [95% CI: 0.05, 0.58]) and C (*b*: 0.36 transformed units [0.09, 0.62]) as compared to placebo. RFD_200_ did not significantly differ based on supplementation, although values were apparently greater in males during the NC (*b*: 660 N/s [−119, 1438]; *p* = 0.10 for NC*male interaction) and C (*b*: 598 N/s [−180, 1377]; *p* = 0.13 for C*male interaction) conditions. PF and RFD_200_ were higher in males as compared to females (*p* ≤ 0.01).

**Conclusions**: The results of this analysis suggest that acute ingestion of either a caffeinated or non-caffeinated pre-workout formulation improves maximal force production during an isometric squat test – but does not appreciably affect late RFD characteristics – when assessed as part of a testing battery in a laboratory environment. As such, trainees aiming to improve maximal force characteristics may benefit from consumption of either a caffeinated or non-caffeinated MIPS prior to their session. Further, these data corroborate the potential utility of a non-caffeinated MIPS for caffeine-sensitive individuals or those training later in the day.

**Acknowledgments**: This study was supported by a research grant from Legion Athletics, Inc. (A21-0096).

**Trial Registration**: ClinicalTrials.gov Identifier: NCT04712578

**Differential Effects of Caffeinated and Stimulant-Free Pre-Workout Supplements on Ratings of Perceived Energy, Focus, and Fatigue**

Jacob J. Green, Patrick S. Harty, Matthew T. Stratton, Madelin R. Siedler, Christian Rodriguez, Jake R. Boykin, Dale S. Keith, Sarah J. White, Brielle DeHaven, Abegale D. Williams, Grant M. Tinsley

Energy Balance & Body Composition Laboratory, Department of Kinesiology & Sport Management, Texas Tech University, Lubbock, TX, USA

Corresponding author: grant.tinsley@ttu.edu

**Background**: Pre-workout supplements are often used by active individuals to augment exercise performance, reduce perceived fatigue, and improve energy levels during prolonged training. However, little information to date is available regarding the effects of non-caffeinated pre-workout supplements on subjective measures of energy, focus, and fatigue compared to similar products which contain caffeine.

**Methods**: 24 resistance-trained males and females (Mean ± SD; 21.0 ± 2.0 years, 171.4 ± 8.7 cm, 72.8 ± 13.5 kg) participated in this investigation. In a randomized, counter-balanced, double-blind fashion, participants consumed either a stimulant-free pre-workout supplement (NC), a caffeine-containing version of the same formulation (C), or a non-caloric placebo. 35 minutes following ingestion, an exercise testing battery was completed, which included bilateral isometric/isokinetic squat performance as well as maximal bench press and leg press testing. Subjective ratings of energy, focus, and fatigue were collected throughout the testing protocol via visual analog scales (0-100). The effects of pre-workout supplementation, biological sex, and time on subjective energy, focus, and fatigue were examined using linear mixed effects models, which also included terms for period and carryover effects, a random intercept for participant, and an autocorrelation structure.

**Results**: For energy, condition×time (*p* = 0.007) and sex×time (*p* = 0.008) interactions were present. After supplement ingestion, energy ratings were higher in C (range of *b*: 14 [95% CI: −3, 32] to 20 [7, 33], *p* = 0.002 to 0.1), but not NC (range of *b: −6* [−22, 11] to 5 [−8, 18], *p* = 0.4 to 0.9), as compared to placebo, for the four post-ingestion assessments spread throughout the exercise testing battery. There were trends for greater decreases in energy for males at the final two time points in the testing battery (*b*: −16 [−33,1] and −17 [−34,0], *p* = 0.06), as compared to females. For fatigue, a sex×time interaction was present (p = 0.002). In males, fatigue was greater at the pre-bench press assessment (*b*: 25 [8, 42], *p* = 0.004), as compared to females. For focus, condition×time (*p* = 0.04) and sex×time (*p* = 0.005) interactions were present. There was a trend for increased focus in C (*b*: 11 [−1, 23], *p* = 0.08), but not NC (*b*: 2 [−10, 14], *p* = 0.78), as compared to placebo, at the first assessment after supplement ingestion. There was also a trend for decreased focus in males at the end of the testing battery (−16 [−34, 1], *p* = 0.07), as compared to females.

**Conclusions**: Caffeinated pre-workout supplements may provide additional benefits for the consumer relative to stimulant-free products, particularly with respect to perceived energy levels during strenuous resistance exercise.

**Trial Registration**: ClinicalTrials.gov Identifier: NCT04712578

**Acute Effects of Caffeinated and Non-Caffeinated Pre-Workout Supplement Consumption on Eccentric and Concentric Force Production During an Isokinetic Squat Exercise**

Jake R. Boykin, Matthew T. Stratton, Madelin R. Siedler, Patrick S. Harty, Christian Rodriguez, Jacob J. Green, Abegale D. Williams, Dale S. Keith, Sarah J. White, Brielle DeHaven, Grant M. Tinsley

Energy Balance & Body Composition Laboratory, Department of Kinesiology & Sport Management, Texas Tech University, Lubbock, TX, USA

Corresponding author: grant.tinsley@ttu.edu

**Background**: Multi-ingredient pre-workout supplements are a common choice for active individuals seeking to improve exercise performance and augment training adaptations over time. These products often contain a blend of ingredients such as caffeine, citrulline, betaine, and beta-alanine, with some products also offered in a caffeine-free version. To date, limited data exists regarding the effects of caffeine-free pre-workout supplements on measures of exercise performance relative to similar caffeine-containing formulations.

**Methods**: Resistance-trained males (n = 12; body mass: 82.7 ± 7.5 kg; fat-free mass index: 21.7 ± 1.8 kg/m^2^; body fat: 18.2 ± 5.8%) and females (n = 12; body mass: 63.0 ± 10.6 kg; fat-free mass index: 16.9 ± 0.8 kg/m^2^; body fat: 26.0 ± 6.7%) were recruited to participate in this investigation. Across three testing visits, participants consumed either a caffeine-free pre-workout supplement (NC), a caffeine-containing version of the same formulation (C), or a non-caloric placebo in a randomized, counter-balanced, double-blind fashion. 35 minutes following supplement ingestion, a maximal isokinetic squat exercise testing protocol was completed as part of a series of laboratory-based exercise performance tests. In short, participants performed one trial repetition on a mechanical squat device, followed by two maximal repetitions. Each repetition consisted of a four-second eccentric phase, a four-second concentric phase, and 1-second pauses at the top and bottom of the movement. All repetitions were completed between 150 degrees and 90 degrees of knee extension. The effects of pre-workout supplementation and biological sex on peak concentric force production (PF_CON_) and peak eccentric force production (PF_ECC_) were examined using linear mixed effects models, which included terms for period and carryover effects and a random intercept for participant.

**Results**: PF_CON_ did not differ significantly between placebo and C (*b*: 71 N [95% CI: −67, 210]) or NC (*b*: 13 N [−125, 152]) conditions. Similarly, PF_ECC_ did not differ significantly between placebo and C (*b*: 57 N [−60, 173]) or NC (*b*: 12 N [−105, 129]). PF_CON_ and PF_ECC_ were higher in males as compared to females (*p* < 0.001).

**Conclusions**: Based on these results, it appears that acute ingestion of either caffeinated or non-caffeinated pre-workout supplement formulations did not result in appreciable improvements in concentric or eccentric peak force production relative to placebo, when assessed as part of a testing battery in a laboratory setting. However, some of the ingredients in these supplements may only provide appreciable ergogenic benefits following chronic consumption. To fully establish the efficacy of such supplementation protocols, evaluation in the context of chronic consumption may be necessary.

**Trial Registration**: ClinicalTrials.gov Identifier: NCT04712578

**The Influence of Caffeinated and Non-Caffeinated Pre-Workout Supplements on Resistance Exercise Performance**

Matthew T. Stratton, Madelin R. Siedler, Patrick S. Harty, Christian Rodriguez, Jake R. Boykin, Jacob J. Green, Dale S. Keith, Sarah J. White, Brielle DeHaven, Abegale D. Williams, Grant M. Tinsley

Energy Balance & Body Composition Laboratory; Department of Kinesiology & Sport Management, Texas Tech University, Lubbock, TX, USA

Corresponding author: grant.tinsley@ttu.edu

**Background**: There is substantial consumer interest in the impact of consuming caffeinated multi-ingredient pre-workout supplements (MIPS). This is partially due to their prevalence in resistance training communities and research demonstrating the efficacy of caffeine intake for improving muscular performance. However, little attention has been paid to the potential efficacy of non-caffeinated MIPS, despite their growing popularity among those who are caffeine-sensitive or train later in the day.

**Methods**: Twenty-four resistance trained college-aged males (n = 12; body mass: 82.7 ± 7.5 kg; fat-free mass index: 21.7 ± 1.8 kg/m^2^; body fat: 18.2 ± 5.8%) and females (n = 12; body mass: 63.0 ± 10.6 kg; fat-free mass index: 16.9 ± 0.8 kg/m^2^; body fat: 26.0 ± 6.7%) completed three visits in which they ingested either a caffeinated MIPS (C), an identical non-caffeinated MIPS (NC), or placebo in a double-blind, randomized, crossover fashion. Upper and lower body maximal muscular strength (1-repetition maximum; 1RM) and endurance (repetitions to failure; RTF) were assessed via bench press and hip sled, respectively, as part of a series of exercise performance tests. The effects of supplementation and biological sex on 1RM and RTF were examined using linear mixed effects models, which included terms for period and carryover effects and a random intercept for participant.

**Results**: Bench press 1RM values did not differ significantly between placebo and C (*b*: 0.6 kg [95% CI: −0.9, 2.1]) or NC (*b*: 0.1 kg [−1.5, 1.6]) conditions. Similarly, bench press RTF did not differ significantly between placebo and C (*b*: 1.2 repetitions [−0.9, 3.3]) or NC (*b*: −1.4 repetitions [−3.5, 0.7]) conditions. Hip sled 1RM did not differ between placebo and C (*b*: 1.4 kg [−4.9, 7.7]), but was lower in the NC condition in males only (*b: −8*.8 kg [−16.6, −0.9]; *p* = 0.03 for NC*male interaction). Hip sled RTF did not differ from placebo for C (*b*: 3.4 repetitions [−1.3, 8.2]) or NC (*b*: 0.3 repetitions [−4.4, 5.0]). 1RM values were higher in males versus females (*p* < 0.001), but no sex differences were observed for RTF (*p* ≥ 0.26).

**Conclusions**: The ingestion of caffeinated or non-caffeinated MIPS did not provide additional benefit to hip sled or bench press performance, as compared to placebo, when these exercises were performed as part of a testing battery in a controlled laboratory setting. These data suggest trainees seeking to improve traditional resistance exercise performance should follow their preferred pre-exercise supplementation strategy but should not feel obligated to consume a caffeinated or non-caffeinated MIPS due to a definitively unique effect of the formulation.

**Acknowledgments**: This study was supported by a research grant from Legion Athletics, Inc. (A21-0096).

**Trial Registration**: ClinicalTrials.gov Identifier: NCT04712578

**Body Composition in Competitive Combat Athletes: a Comparison Between the InBody and DXA**

Jose Rojas^a,b^, Jose Antonio^a^

^a^Exercise and Sport Science, NSU Florida, Davie Florida, USA; ^b^Daru Strong Performance, Boca Raton, Florida, USA

Corresponding author: Jose.antonio@nova.edu

**Background**: The purpose of this study was to assess the differences in body composition assessments using InBody and DXA.

**Methods**: Competitive combat athletes (men n = 6, women n = 9; mean ± SD: age 29.1 ± 3.6; height 1.69 ± 0.08 meters; weight 73.3 ± 11.7 kilograms) were assessed for body composition via the InBody270 and DXA. Body fat percentage, lean body mass, and fat mass from InBody and DXA were compared.

**Results**: No significant differences in body composition were observed between the two instruments (Table 1). No significant differences in body composition were observed between the two instruments when the data was separated by sex (Table 2).

**Table 1.** Comparison between the InBody and DXA

	In Body	DXA
Body Fat Percentage	17.7 ± 4.7	18.0 ± 4.6
Body Fat (kg)	13.3 ± 12.3	13.5 ± 2.7
Lean Body Mass (kg)	63.9 ± 12.3	62.7 ± 12.1

**Table 2.** Body composition by method and sex

	InBody (male)	DXA (male)	InBody (female)	DXA (female)
Body Fat Percentage	14.9 ± 1.3	15.0 ± 1.4	21.9 ± 5.1	22.7 ± 3.6
Fat Mass (kg)	12.6 ± 2.0	12.7 ± 2.3	14.3 ± 3.8	14.7 ± 3.0
Lean Body Mass (kg)	71.9 ± 6.9	71.2 ± 6.5	50.9 ± 5.8	50.1 ± 4.2

**Table 1.** Body composition and RMR changes over time

	Baseline	1-Week Prior to Competition 1	1-Week Prior to Competition 2
Body Mass (kg)	152.1	134.7 (-Δ17.4)	136.3 (-Δ15.8)
Body Fat %	18.8	12.5 (-Δ6.3)	12.2 (-Δ6.6)
Fat Mass (kg)	28.6	16.8 (-Δ11.8)	16.6 (-Δ12)
Fat-Free Mass (kg)	123.5	117.9 (-Δ5.6)	119.7 (-Δ3.8)
RMR (kcal/d)	1725	1376 (-Δ349)	1540 (-Δ185)

Data are expressed as the mean ± SD. Legend: kg – kilogram.

Data are expressed as the mean ± SD. Legend: kg – kilogram

**Conclusions**: Each instrument assesses different compartments. InBody assesses total body water while DXA assesses bone mineral content and density. However, no differences were found between these methods regarding fat mass, percent body fat, or lean body mass. These findings suggest that both InBody and DXA are suitable for body composition testing in elite combat athletes.

**Effects of a Single Dose of BURN-XT™ on Resting Metabolic Rate, Substrate Oxidation, and Various Indices of Affect**

Michael La Monica^a^, Betsy Raub^a^, Jennifer Sandrock^a^, Hector L. Lopez^a^, Tim N. Ziegenfuss^a^

^a^The Center for Applied Health Sciences, Canfield, OH 44406, USA

Corresponding author: TZ@appliedhealthsciences.org

**Background**: Many consumers use dietary supplements in the hopes of increasing energy and burning more calories, which if sustained over time may help accelerate weight loss. The purpose of this clinical trial was to investigate the effects of an over-the-counter thermogenic supplement called Burn-XT™ (BXT), on metabolic rate, substrate oxidation, and various indices of affect that impact weight management.

**Methods**: Using a double-blind, placebo-controlled, cross-over design, 16 females and 10 males (29.3 ± 7.3 yr, 169.4 ± 8.6 cm, 75.5 ± 14.3 kg) underwent two testing sessions: placebo (PLA) and BXT. Seated metabolic rate and substrate oxidation (ParvoMedics TrueOne 2400), vital signs, and anchored Visual Analogue Scale (VAS) assessments of energy, mood, motivation, focus, fatigue, concentration, and appetite were made before supplementation and at 60-min intervals for three hours post-ingestion. Two-factor (2 x 4) factorial ANOVAs and paired sample t-tests (corrected for multiple comparisons) were used to analyze the data. Statistical significance was set *a priori* at p ≤ 0.05 and trends were declared at 0.051 ≤ p ≤ 0.10.

**Results**: Significant increases in metabolic rate (oxygen consumption) were noted at 60 minutes in BXT (+11.9 mL O_2_/min) vs. PLA (−2.5 mL O_2_/min), p = 0.004. Only BXT increased metabolic rate compared to baseline at 60 minutes (+ 11.9 mL O_2_/min, p = 0.02) and 120 minutes (+ 12.1 mL O_2_/min, p = 0.019). VAS detected significant improvements in energy, mood, focus, and concentration for BXT vs. PLA at 120 and 180 minutes (all p < 0.05). In all cases, within-group changes from baseline for these VAS parameters were significant (all p < 0.05) in BXT but not in PLA. No within or between-group differences in appetite, substrate oxidation, heart rate, or systolic blood pressure were noted. Small (~4 mm Hg), but statistically significant (p < 0.05) increases in diastolic blood pressure were noted in BXT at 60, 120, and 180 min vs. PLA; in all cases, values remained within normal clinical hemodynamic ranges.

**Conclusions**: A single dose of BXT safely increased metabolic rate, energy, mood, focus, and concentration. Given that these factors are known to favorably impact weight management, future studies should determine whether daily supplementation with BXT reduces body weight and improves body composition.

**Acknowledgments**: This study was funded by NutraHoldings, Inc. The authors declare no COI.

**Celiac Disease and Elite Athlete Six Month Report on Swimming: A case study**

Roberto Cannataro^a,b^, Andrea Malorgio^c^, Marta Malorgio^c^, Matteo Levi Micheli^d,e^, Erika Cione^a,b^, and Richard B Kreider FISSN^f^

^a^Galascreen Laboratories, University of Calabria Rende (CS), Italy; ^b^Department of Pharmacy, Health and Nutritional Sciences, University of Calabria, Rende (CS), Italy; ^c^University of Pisa Clinical and Experimental Department, Via Savi 10, 56,126 Pisa, Italy; ^d^Be Active, Cascina (PI), Italy; ^e^Department of Experimental and Clinical Medicine, University of Florence, Florence, Italy; ^f^Nutrition M. Marella Laboratory of Motor Sciences Applied to Medicine, Florence, Italy; ^g^Exercise and Sport Nutrition Laboratory, Human Clinical Research Facility, Texas A&M University, College Station Texas, USA

Corresponding author: rcannataro@nutrics.it

**Background**: Celiac disease is still probably underdiagnosed, but which counts around 1% of the population^1^. This report aims to demonstrate how even in this condition, if under careful supervision, results of absolute sports excellence can be obtained. Strangely apart from the generic indications from, for example, the American College Sports Medicine (ACSM) as well as the International Society of Sports Nutrition (ISSN), there are few cases in the literature of high-level athletes with celiac disease^2^. Some report cases in which an athlete is managed who did not know he was celiac, but not the period of a season or more^3,4^.

**Methods**: Herein we reported the case of a male 21 aged swimmer come to our laboratory in September 2020. He showed difficulty in increasing muscle mass and fatigue, especially at the end of training sessions. We performed bio-impedance and stratigraphy analysis on the abdomen each month. At the same time, clinical, biochemical analysis was performed in three points. Timing test and 1RM test were performed. From the perspective of nutrition, our intervention was to limit foods designed for celiacs as much as possible, preferring naturally gluten-free foods. Therefore, for carbohydrates, mainly rice, amaranth, potatoes, pumpkin and beetroot, and legumes. We have emphasized the regular intake of fruit and vegetables, strongly limiting glycemic peaks (not always easy given the necessary caloric amount to reach). Proteins were set around 2.2 gxKg of body weight. The sources of fats were, mainly, extra virgin olive oil and nuts, in addition to those related to protein sources (i.e.: salmon, eggs). Following the indications of the ISSN, we recommended daily omega 3 fish oil (2 g, 1,2 g of DHA + EPA), 1 g of vitamin C (in two doses of 500 mg), Vitamin D (2000UI he showed a deficiency in the first tests), magnesium (300 mg as pidolate). Every other month vitamin B12 (1000mcg), creatine (5 g), beta-alanine (4 g); whey protein isolate, and maltodextrin in the post-workout and when needed.

**Results**: Our intervention was aimed at an increase in muscle mass and a performance improvement. In March 2021, the subject obtained the seventh place in the absolute Italian championships (50 m breaststroke), in which the first classified obtained the best seasonal world performance. Overall, from the BIA analysis, it has improved a lot, + 1.5 Kg in weight but a 10% in PhA, which denotes a much better condition. The stratigraphy noted how the thickness of fat on the abdomen is unchanged; it is likely that an increase in muscle mass. His condition worsened in the training period due to an intestinal infection, which the subject reported encountering at times, but promptly recovered with an excellent condition for the Italian championships. His plasma values show an increase in hemoglobin and ferritin. The increase of this latter parameter was almost double in the last check, having a positive effect on performance. In the first check, a slight leukopenia is noted. We hypothesize that leukopenia was due to a subclinical inflammatory state, completely restored in the following two check control. The athlete does not show any seasonal respiratory syndrome nor contracts COVID-19; the overall performance recorded a decrease in times of 0.87 ‘, 3’ 23, and 2 “53 respectively in the 50, 100, and 200 m, while an increase of 18,75, 17,86 and 23,68% in the 1RM of bench press, deadlift and squat, respectively.

**Conclusions**: Our is one of the first detailed management reports of a celiac athlete; we want to emphasize that with careful and evidence-based supervision, results of absolute excellence can be obtained even in conditions of pathologies such as celiac disease. It would be fascinating to have even more accurate evaluations and an even longer time, which we are continuing to operate.

**Acknowledgments**: No conflict of interest or sponsorship to mention.

**References**

^1^Lebwohl B, Sanders DS, Green PHR. Celiac disease. Lancet. 6 January 2018;391(10,115):70-81. DOI: 10.1016/S0140-6736(17)31,796-8. Epub 28 July 2017^2^Mancini LA, Trojian T, Mancini AC. Celiac disease and the athlete. Curr Sports Med Rep. 2011 Mar-Apr;10(2):105-8. DOI: 10.1249/JSR.0b013e31820f2eab

^3^Leone JE, Gray KA, Massie JE, Rossi JM. Celiac disease symptoms in a female collegiate tennis player: a case report. J Athl Train. 2005 Oct-Dec;40(4):365-9. PMID: 16,404,460

^4^Eberman LE, Cleary MA. Celiac disease in an elite female collegiate volleyball athlete: a case report. J Athl Train. 2005 Oct-Dec;40(4):360-4. PMID: 16,404,459; PMCID: PMC1323299

**Case study: Changes in Body Composition and Resting Metabolic Rate in an Experienced Female Physique Athlete**

Longstrom J, Phillips K, Baxter H, Norton L, Mastrofini G, Broeckel J, Morrisseau B, and Campbell BI

Performance and Physique Enhancement Laboratory, Exercise Science Program, University of South Florida, Tampa, FL, USA

Corresponding author: bcampbell@usf.edu

**Background**: Physique athletes undergo strict diet and exercise regimens during contest preparation to achieve low body fat levels for competitive success. Prolonged energy restriction and weight loss make it challenging for experienced competitors to improve their aesthetic appearance between contests during a competitive season. The purpose of this case study was to evaluate changes in body composition and resting metabolic rate (RMR) of an experienced, natural female figure competitor throughout contest preparation and 1-week prior to two competitions, separated by four months.

**Methods**: Three laboratory assessments were conducted over 8 months. At each visit, body composition (fat mass [FM] and fat-free mass [FFM]) and resting metabolic rate (RMR) were assessed 1-week prior to each competition, before employing a carb load peaking strategy. All assessments were performed following an 8-hour overnight fast.

**Results**: Body mass decreased by 17.4 kg prior to the first competition and increased by 1.6 kg before the second competition. Body fat was reduced from 18.8% to 12.5% prior to the first competition and declined to 12.2% prior to the second competition. FM decreased from 28.6 kg to 16.8 kg before the first competition and declined to 16.6 kg before the second competition. FFM decreased from 123.5 kg to 117.9 kg before the first competition and increased to 119.7 kg before the second competition. RMR decreased from 1,725 kcal/d to 1,376 kcal/d prior to the first competition and increased to 1540 kcal/d prior to the second competition. Table 1 details results.

Data are raw values (change scores relative to baseline)

**Conclusions**: This study contributes to the currently sparse amount of data on physique athletes preparing for multiple competitions during a competitive season. Despite an increase in body weight between the two competitions, body fat was further reduced from contest 1 to contest 2 which also coincided with increases in FFM and RMR in an experienced female figure competitor.

**Efficacy of an Enzymatically Enhanced Spinach Supplement, Solarplast®, on Inflammation**

Matthew Lee^a^, Trisha A. VanDusseldorp^a^, Michaela Alesi^a^, Jackie Easter^a^, Alyssa R. Bailly^a^, Matthew T. Stratton^b^, Constantine Katsoudas^a^, Katie Tran^a^, Garrett M. Hester^a^

^a^Department of Exercise Science and Sport Management, Kennesaw State University, Kennesaw, GA, USA; ^b^Department of Kinesiology and Sport Management, Texas Tech University, Lubbock, TX, USA

Corresponding author: tvanduss@kennesaw.edu

**Introduction**: Solarplast® is a recently developed enzymatically enhanced supplement derived from spinach. Solarplast® contains antioxidants that are hypothesized to reduce inflammation. Though humans are generally capable of managing inflammation, these responses can become weakened with age or through environmental factors. The aim of this study was to investigate the efficacy of Solarplast® supplementation in reducing systemic inflammation.

**Methods**: Sixty-eight healthy individuals completed this study (Solarplast® 25.7 ± 9.7 yrs, 71.7 ± 17.4 kg; placebo 27.2 ± 13.4 yrs, 67.6 ± 13.2 kg). Participants were asked to arrive at the laboratory for a blood draw in a fasted state for both visits (Pre and Post) which were separated by 45 days. At the end of visit one, participants were randomly assigned to either supplementation group (Solarplast ® or placebo; 100 mg/day). Resulting serum and plasma from blood collection was aliquoted and stored in a − 80ᵒC freezer until analysis via enzyme-linked immunosorbent assay (ELISA). Markers of inflammation (TNF-alpha, IL-6, and IL-4) were measured in duplicate. Data were analyzed via a 2 (Solarplast ® vs placebo) x 2 (pre and post) analysis of variance. If significant interactions were detected, post-hoc paired sample t-tests were utilized to identify where differences occurred. Significance was set *a priori* at an alpha level of *p* ≤ 0.05.

**Results**: There was an interaction (p < 0.001) for TNF-alpha as it decreased in Solarplast® (p = 0.024) compared to placebo. A main effect for time was noted for IL-4 (p = 0.016) and IL-6 (p = 0.038) since both groups decreased.

**Conclusions**:. The reduction in TNF-alpha in healthy individuals who supplemented with Solarplast® for 45 days is interesting, as TNF-alpha is a well-known pro-inflammatory cytokine responsible for the modulation of the immune system. The contribution of molecular chaperone activity via daily Solarplast® supplementation may be responsible for the reduction; however, more research needs to be conducted. Further, future research on Solarplast® should be conducted in individuals who experience high basal levels of inflammation, such as clinical populations.

**Acknowledgments**: This research was funded by Deerland Enzymes and Probiotics via a grant received by Trisha A. VanDusseldorp (PI). The PI asserts Deerland Enzymes and Probiotics was not involved in data collection or analysis and had no influence on the outcomes of this research. We would also like to thank the participants who dedicated time to complete the study.

**Validation of a Vastus Lateralis Glycogen Depletion Protocol Using an Air-Braked Rowing Ergometer**

Neil A. Schwarz^a^, Brandon R. Funderburg^a^, Geoffrey M. Hudson^a^, Shelley L. Holden^a^

^a^University of South Alabama, Mobile, AL, USA

Corresponding author: neilschwarz@southalabama.edu

**Background**: The purpose of this study was to validate the effectiveness of a novel vastus lateralis glycogen depletion protocol using an air-braked rowing ergometer by comparing it with a previously established cycling protocol.

**Methods**: This study utilized a randomized, cross-over design. Seven participants (4 males, 3 females) with prior rowing experience aged 23.9 ± 1.6 years with a height and body mass of 169.6 ± 12.3 cm and 73.5 ± 17.1 kg, respectively, and a body fat percentage of 20.4 ± 4.0% completed the study. Participants completed an entry session and two testing sessions. During the entry session, informed consent was obtained followed by a DXA scan, a peak oxygen consumption test using a cycle ergometer, and a 500-m time trial on an air-braked rowing ergometer. Testing sessions consisted of an established cycle ergometer glycogen depletion protocol or a novel rowing ergometer glycogen depletion protocol. The cycle ergometer glycogen depletion protocol consisted of intermittent cycling beginning at a working intensity of 90% of Wmax obtained during their peak oxygen test. Participants cycled at 90% Wmax for two minutes followed by a recovery intensity of 50% Wmax for two minutes. When participants could no longer maintain the working intensity, it was reduced by 10% beginning with the next interval. Participants cycled until a working intensity of 60% Wmax could no longer be maintained. The rowing ergometer glycogen depletion protocol consisted of intermittent 500-m rows. To begin, participants were expected to row 500 meters at a minimum of 90% of their maximal 500-m row pace (calculated as 500-m row time divided by .90). Once the participant could not keep the pace for two consecutive rows, rowing pace was decreased by 5% until two rows at 70% could not be maintained or a maximum of forty 500-m rows were completed. Two minutes of rest were provided between rows at the 90% and 85% intensities. Rest time was reduced to one minute at intensities of 80% and below. Muscle biopsies were obtained immediately before and after each exercise protocol. To collect metabolic data, participants wore a face mask connected to a gas analyzer during each exercise protocol.

**Results**: For glycogen content, no significant main effect for condition (*p* = 0.883) or interaction between condition and time were observed (*p* = 0.52). A significant main effect for time was observed with exercise intervention resulting in a decrease of muscle glycogen content from 4.29 ± 1.46 nmol/mg to 2.60 ± 1.50 nmol/mg (*p* = 0.004). Cycling resulted in a significant decrease in glycogen content from 4.19 ± 1.42 nmol/mg to 2.59 ± 1.68 nmol/mg (*p* = 0.014). Rowing resulted in a similar significant decrease in glycogen content from 4.39 ± 1.62 nmol/mg to 2.61 ± 1.43 nmol/mg (*p* = 0.001). Duration of the rowing protocol was significantly longer than the cycling protocol (101.6 ± 38.5 min vs. 59.6 ± 14.1 min; *p* = 0.050). Exercise energy expenditure was significantly greater during the rowing protocol than the cycling protocol (1204 ± 462 kcal vs. 662 ± 257 kcal; *p* = 0.013).

**Conclusions**: The novel glycogen depletion protocol utilizing the rowing ergometer was capable of producing a similar magnitude of glycogen reduction in the vastus lateralis muscle when compared with a previously established cycling protocol. The rowing protocol, due to the utilization of greater amount of muscle mass, resulted in a significantly greater energy expenditure suggesting a larger overall total skeletal muscle glycogen reduction. However, the rowing protocol took significantly more time to complete. If localized glycogen depletion of the vastus lateralis muscle is the main goal, it may be more time efficient to use the cycling protocol. If greater overall glycogen depletion is necessary or useful, especially for exercise and nutrition-related studies, the rowing protocol may be a suitable choice as it is likely to have resulted in a greater overall consumption of glycogen stores beyond the vastus lateralis.

**Acknowledgments**: This study was supported through the Graduate Student Activities Enhancement Program sponsored by the Graduate School at the University of South Alabama.

**Efficacy and Safety of an Enzymatically Enhanced Spinach Supplement, Solarplast®, in Nonsmokers and Smokers**

Jennifer A. Kurtz^a,^ Trisha A. VanDusseldorp^b^, Jackie Easter^b^, Michaela G. Alesi^b^, Alyssa R. Bailly^b^, Matthew T. Stratton^c^, Constantine Katsoudas^b^, Katie Tran^b^, Matthew Lee^b^, Garrett M. Hester^b^

^a^Exercise Physiology Laboratory, Department of Kinesiology and Health, Georgia State University, Atlanta, GA, USA; ^b^Department of Kinesiology and Sport Management, Kennesaw State University, Kennesaw, GA, USA; ^c^Texas Tech University, Lubbock, TX, USA

Corresponding author: tvanduss@kennesaw.edu

**Background**: Spinach, or *Spinacia oleracea*, contains numerous active components, including flavonoids, which exhibit antioxidative and anti-inflammatory properties. Solarplast® is an enzymatically treated spinach extract, developed by Deerland Probiotics and Enzymes, which is derived from *Spinacia oleracea*. More specifically, Solarplast® contains photosynthetic complexes with high concentrations of ATP, NADPH, ADP, AMP, NADP, niacin, B12, adenine, and ribose. The unique mixture of molecules is thought to attack oxidants through chaperone activity. This study was designed to investigate the effects of 45 day Solarplast® supplementation on metabolic safety and oxidative stress, while secondary assessments included questionnaires associated with skin, physical, and mental health in smokers and nonsmokers.

**Methods**: A total of 68 healthy, nonsmoking individuals (25.7 ± 9.7 yrs.) and 16 smokers (28.1 ± 13.9 yrs.) completed the study. Smoking was defined as individuals who had smoked every day for the last year at the time of enrollment – termed ‘every-day smoker’. Participants were divided into nonsmokers and smokers and randomly assigned to consume either once daily Solarplast® (100 mg/day; 1 × 10^6^ light converting units [LCUs]) or placebo (100 mg/day maltodextrin) for 45 days. Single venipunctures at the antecubital space were used to collect blood samples to analyze participant’s metabolic panel and oxidative stress markers. The skin visual analog scale (SKN-VAS) questionnaire was used to assess participant’s perception of skin changes. The Anti-Aging QOL Common Questionnaire (AAQOL) was also collected to track an individual’s physical health and mental symptoms, and lifestyle-related questions, including sleep, drinking, smoking, and exercise habits.

**Results**: *Nonsmokers*: There were no significant changes in a comprehensive metabolic panel (p > 0.05). There were no significant changes found for ROS/RNS or GSH/GSSG ratio. A group x time interaction was noted for pain (p = 0.031), where significant decreases were found at post (p = 0.026). A group x time interaction (p = 0.002) was found for skin problems, where significant differences at post indicated decreases in skin problems for those who supplemented with Solarplast® (p = 0.010). A group x time interaction was also found for anxiety (p = 0.002) and irritability (p = 0.025), which both decreased significantly. *Smokers*: There were no significant changes in a comprehensive metabolic panel. There were no significant changes found for ROS/RNS or GSH/GSSG ratio. A group x time interaction effect was found for ROS/RNS (*p* = 0.046), with a significant decrease at post (*p* = 0.031) for Solarplast® supplementation; no changes in GSH/GSSH were detected. Depression was significantly decreased (p = 0.029) at the post for Solarplast® supplemented individuals.

**Conclusions**: Results of this investigation indicate 45 days of Solarplast® supplementation was safe, but ineffective at reducing markers of oxidative stress (ROS/RNS) or altering GSG/GSSH in young, healthy individuals. However, in smokers, oxidative stress was decreased following Solarplast® . This is likely due to the higher basal levels of oxidative stress experienced by smokers. Interestingly, mood alterations were experienced by Solarplast supplemented individuals in both groups. Antioxidants have previously been reported to improve mood; however, the mechanistic impact of Solarplast® on these outcomes needs to be further examined. Future research should consider longer supplementation in clinical individuals who experience higher levels of oxidative stress and inflammation, such as type 2 diabetic patients.

**Acknowledgments**: This research was funded by Deerland Enzymes and Probiotics.

**A Comparison Between Recommended Macronutrient Intake and Reported Intake of Collegiate Women’s Volleyball Players**

Shelley L. Holden, Neil A. Schwarz

Department of Health, Kinesiology and Sport, University of South Alabama, Mobile, AL, USA

**Background**: The purpose of this study was to compare the calculated International Society of Sports Nutrition (ISSN) recommendations for daily macronutrient and caloric intake to the actual intake of collegiate female volleyball players at two points in the competitive season (pre-season and mid-season).

**Methods**: Participants (*n* = 11) completed a 3-day diet log during the first three days of the pre-season and a 4-day diet log during mid-season competition. Participants were assessed for body mass during the pre-season which was used to calculate recommended intake values for the macronutrients (carbohydrates, proteins, fats) and energy (kilocalories). Dietary intake was self-reported using a commercially available food tracking program (*MyFitnessPal©, USA*). Daily average values were calculated for total energy, protein, carbohydrate, and fat intake during the pre-season and mid-season monitoring periods. These values were then compared to nutritional recommendations set forth by the ISSN for team sport athletes undergoing a similar level of training. This equated to recommended values of 50 kilocalories per kilogram of body weight per day (kg/d), 6.5 g/kg/d, and 1.8 g/kg/d for total energy, carbohydrates and proteins, respectively. A recommended fat intake equating to 30% of the recommended energy intake was used. Data (presented as mean ± standard deviation) were analyzed using separate repeated-measures ANOVA between ISSN recommendations, pre-season, and mid-season for fat, protein, carbohydrate, and energy intake. Significant main effects (denoted by *p* < 0.05) were investigated further using *t* tests with Bonferroni correction applied.

**Results**: The volleyball players had a mean body mass of 71.7 ± 8.3 kg and body mass index of 23.0 ± 1.7 kg/m^2^. The ISSN recommendations for energy, carbohydrate, fat, and protein were 3586 ± 415 kcal, 467 ± 54 grams, 120 ± 14 grams, and 129 ± 15 grams, respectively. A significant main effect was observed between ISSN recommendations, pre-season, and mid-season for energy intake (*p* < 0.001), carbohydrate intake (*p* < 0.001), fat intake (*p* = 0.01), and protein intake (*p* = 0.03). For energy intake, no difference (*p* = 1.00) was observed between pre-season (2214 ± 720 kcal) and mid-season (2303 ± 640 kcal). Both pre-season energy intake (*p* < 0.001) and mid-season energy intake (*p* < 0.001) were significantly lower than the ISSN recommendation. For carbohydrate intake, no difference (*p* = 1.00) was observed between pre-season (260 ± 91 grams) and mid-season (234 ± 73 grams). Both pre-season carbohydrate intake (*p* < 0.001) and mid-season carbohydrate intake (*p* < 0.001) were significantly lower than the ISSN recommendation. For fat intake, no difference (*p* = 0.714) was observed between pre-season (82 ± 32 grams) and mid-season (98 ± 31 grams). Pre-season fat intake was significantly lower than the ISSN recommendation (*p* = 0.003), whereas no difference was observed between mid-season fat intake (*p* < 0.162) and the ISSN recommendation. For protein intake, no difference (*p* = 0.209) was observed between pre-season (120 ± 38 grams) and mid-season (102 ± 22 grams). Pre-season protein intake was similar to the ISSN recommendation (*p* = 1.00). Mid-season protein intake was significantly lower than the ISSN recommendation (*p* < 0.001).

**Conclusions**: Based upon the results of the current study and previous studies, nutrition education and/or interventions may be necessary for female collegiate volleyball players. Education and/or interventions need to focus on making sure the athletes achieve the minimum energy intake while considering macronutrient distribution. Explaining the functions of each of the macronutrients as well as the importance of meeting caloric recommendations may help these athletes strive to meet the recommendations and improve recovery and performance.

**KETAD Trial: Effects of Exogenous Ketones as an Adjunct to Low-Calorie Diet on Metabolic Biomarkers, Fat Loss and Health**

Hector L. Lopez^a^, Betsy Raub^a^, Kyle Cesareo^a^, Jennifer Sandrock^a^, Chad Kerksick^b^, Tim N. Ziegenfuss^a^

^a^The Center for Applied Health Sciences, Canfield, OH 44406; ^b^Exercise & Performance Nutrition Laboratory, Lindenwood University, St. Charles, MO 63301, USA

Corresponding author: HL@appliedhealthsciences.org

**Background**: Inducing ketonemia through exogenous ketone administration is of interest as a potential adjunct to facilitate weight loss and improve health outcomes, however, minimal research has been completed examining the impact of exogenous ketones on these parameters. The purpose of this study was to quantify changes in weight loss, body composition, hunger, appetite, and health outcomes from ingesting different forms of exogenous ketone supplements.

**Methods**: Overweight and obese men (n = 44) and women (n = 60) between the ages of 18 – 46 years (34.6 ± 6.7 yr, 171.2 ± 10.3 cm, 92.6 ± 14.9 kg, 31.4 ± 2.9 kg/m^2^) were randomly assigned in a double-blind, placebo-controlled manner to supplement with one of three different commercially available exogenous ketone formulations: [Real Ketones^TM^ Quad Electrolyte BHB (RK-QBHB), Real Ketones^TM^ Quad Electrolyte BHB-MCT1 (QBHB-MCT1), Real Ketones^TM^ Quad Electrolyte D-weighted BHB-MCT2 (QBHB-MCT2)] or a carbohydrate placebo (PLA) for eight weeks. Participants were instructed to perform 30 minutes of walking at least 3 days per week and follow an energy-restricted diet (500 kcals below predicted needs) throughout the study. Body composition (DEXA), metabolic rate, clinical safety markers, and plasma [insulin] were assessed before and after eight weeks of supplementation. Additionally, changes in various indicators of affect (appetite, hunger, mood, cravings, mental clarity, fatigue) were assessed using visual analog scales 0, 60, 120 and 180 minutes post (acute) ingestion before and after eight weeks of supplementation. Statistical significance was set *a priori* at p ≤ 0.05 and trends were declared at 0.05 ≤ p ≤ 0.10.

**Results**: All groups lost significant amounts of body weight and body fat (p < 0.001). RK-QBHB lost significantly greater amounts of body weight (−3.05 ± 2.53 kg) and body fat (−1.11 ± 2.03%), and had greater reductions in hip circumference (−4.6 ± 5.4 cm) than PLA (−0.8 ± 4.0 cm, p = 0.007) and QBHB-MCT1 (−1.85 ± 5.5 cm, p = 0.046). No changes were observed in blood pressure, heart rate, adverse events, fasting insulin, or standard clinical safety markers.

**Conclusions**: Eight weeks of caloric restriction and walking exercise promotes decreases in body weight and body fat. Co-supplementation with exogenous ketones, in particular RK-QBHB, leads to greater benefits in some anthropometric parameters and may improve aspects of blood lipids, such as LDL cholesterol. In all groups, supplementation was well tolerated with no significant deleterious effects on blood pressure, heart rate, adverse events, insulin or other whole-blood and serum markers of clinical safety.

**Acknowledgments**: This study was funded by Axcess-Global Sciences, LLC. The authors declare no COI.

**Vitamin D Deficiency in Historic Industrial Athlete Skeletal Remains**

Kristy Henson^a,b^

^a^School of Archeology and Ancient History, University of Leicester, Leicester, LE17RH, UK.; ^b^College of Science and Technology, Fairmont State University, Fairmont, WV, 26,554. USA

Corresponding author: kdh9@leicester.ac.uk

**Background**: During the 1800 and early 1900s many people worked physically demanding occupations in industrializing America. Long hours underground or in polluted cities with limited access to resources may have increased laborers’ susceptibility to vitamin D deficiency. Vitamin D is activated by ultraviolet-B radiation through direct exposure to the skin. Other sources of vitamin D during this time included mushrooms and oily fish. Individuals who do not receive enough vitamin D lack adequate blood calcium levels, leading to metabolic deficiencies and health problems, like rickets and osteomalacia. Vitamin D deficiency is a universal problem, and biocultural variables such as nutritional availability, residence, weather, and occupation directly correlate. This research focuses on the impact of vitamin D deficiency in the recent historic past and how biocultural variables influence the likelihood of industrial athletes being vitamin D deficient. Posthumous examination enables macroscopic inspection that may provide greater detail than a CT/MRI.

**Methods**: Known skeletal remains were macroscopically analyzed for osteomalacia and residual rickets. Relevant personal information for the individuals was recorded from death certificates. Death certificate data allow tracking of individuals through the US Census. Residences and occupations were the two key biocultural variables explored in this study. Historic weather data such as temperature, UV index, and zenith angle were extracted for states settled and surrounding the Mason-Dixon line after 1820. Weather data were averaged by state and season. Data were analyzed using ANOVA and PCA to give a quantitative basis for understanding vitamin D deficiency in relation to the potential to synthesize adequate vitamin D in each state.

**Results**: Preliminary ANOVA results show that weather variables varied significantly by state (p < 0.05; F < F crit). Multivariate PCA indicates that northern states negatively correlate with temperature and UV index while southern states positively correlate with temperature and UV index. State of residence may directly impact one’s vitamin D status if ultraviolet-B skin penetration is unimpeded.

**Conclusions**: Northern versus southern states have significantly different seasonal temperature, UV index, and zenith angles. Individuals living above the Mason-Dixon line may not have access to sufficient UVB radiation throughout the year, which may increase their likelihood of developing osteomalacia. These individuals would need to supplement vitamin D through edibles like mushrooms and oily fish, though these would have been available only to laborers living in certain geographic locations. Luxury items shipped inland would not have been obtainable by common laborers, potentially increasing their chances of osteomalacia.

**Acknowledgments**: Dr. Jo Appleby, Dr. Richard Thomas, and Dr. Greg Popovich.

WVHEPC DSR Opportunity Grant DSR.20.25

